# Albumin Administration in Acute Ischemic Stroke: Safety Analysis of the ALIAS Part 2 Multicenter Trial

**DOI:** 10.1371/journal.pone.0131390

**Published:** 2015-09-01

**Authors:** Michael D. Hill, Renee H. Martin, Yuko Y. Palesch, Claudia S. Moy, Diego Tamariz, Karla J. Ryckborst, Elizabeth B. Jones, David Weisman, Creed Pettigrew, Myron D. Ginsberg

**Affiliations:** 1 Departments of Clinical Neurosciences, Medicine, Radiology and Community Health Sciences, Hotchkiss Brain Institute, Cumming School of Medicine, University of Calgary, Calgary, AB, Canada; 2 Department of Public Health Sciences, Medical University of South Carolina, Charleston, South Carolina, United States of America; 3 Office of Clinical Research, National Institute of Neurological Disorders and Stroke, National Institutes of Health, Bethesda, Maryland, United States of America; 4 Department of Neurology, University of Miami Miller School of Medicine, Miami, Florida, United States of America; 5 Department of Clinical Neurosciences, Cumming School of Medicine, University of Calgary, Calgary, AB, Canada; 6 Department of Emergency Medicine, University of Texas Health Science Center at Houston, Houston, Texas, United States of America; 7 Abington Neurological Associates, Abington, Pennsylvania, United States of America; 8 Department of Neurology and Sanders-Brown Center on Aging, University of Kentucky, Lexington, Kentuck, United States of America; University of Regensburg, GERMANY

## Abstract

**Background:**

Albumin treatment of ischemic stroke was associated with cardiopulmonary adverse events in previous studies and a low incidence of intracranial hemorrhage. We sought to describe the neurological and cardiopulmonary adverse events in the ALIAS Part 2 Multicenter Trial.

**Methods:**

Ischemic stroke patients, aged 18–83 and a baseline NIHSS ≥ 6, were randomized to treatment with ALB or saline control within 5 hours of stroke onset. Neurological adverse events included symptomatic intracranial hemorrhage, hemicraniectomy, neurological deterioration and neurological death. Cardiopulmonary adverse events included pulmonary edema/congestive heart failure, acute coronary syndromes, atrial fibrillation, pneumonia and pulmonary thromboembolism.

**Results:**

Among 830 patients, neurological and cardiopulmonary adverse events were not differentially associated with poor outcome between ALB and saline control subjects. The rate of symptomatic intracranial hemorrhage in the first 24h was low overall (2.9%, 24/830) but more common in the ALB treated subjects (RR = 2.4, CI_95_ 1.01–5.8). The rate of pulmonary edema/CHF in the first 48h was 7.9% (59/830) and was more common among ALB treated subjects (RR = 10.7, CI_95_ 4.3–26.6); this complication was expected and was satisfactorily managed with mandated diuretic administration and intravenous fluid guidelines. Troponin elevations in the first 48h were common, occurring without ECG change or cardiac symptoms in 52 subjects (12.5%).

**Conclusions:**

ALB therapy was associated with an increase in symptomatic ICH and pulmonary edema/congestive heart failure but this did not affect final outcomes. Troponin elevation occurs routinely in the first 48 hours after acute ischemic stroke.

**Trial Registration:**

ClincalTrials.gov NCT00235495

## Introduction

High-dose human albumin was shown in pre-clinical rodent stroke models to be a highly neuroprotective agent.[1, 2] Albumin reduces infarction volume and cerebral edema, and improves behavioral function, with a therapeutic window of efficacy of at least 4 hours after stroke onset.[1, 3] Albumin normalizes the apparent diffusion coefficient within the residual infarct, relieves obstructions in postischemic cortical venules[4], and improves microvascular hemodynamics distal to an arterial thrombosis.[5–7]

On the basis of these and other preclinical findings, we hypothesized that high-dose albumin administration would improve neurological and functional outcome in patients with acute ischemic stroke. In the Albumin in Acute Stroke (ALIAS) Pilot Clinical Trial, we first established the dosing regimen and defined a 12–14% risk of mild-moderate pulmonary edema or congestive heart failure as an expected adverse event of high-dose albumin therapy.[8] The ALIAS Part 1 Trial then tested the hypothesis that 2g/kg of 25% human albumin infused intravenously within 5 hours of ischemic stroke onset would improve outcome measured at 90 days.[9] Enrollment in the Part 1 Trial was halted after 434 patients due to increased mortality in the treatment arm.[8, 10] In ALIAS Part 1, congestive heart failure and pulmonary edema were predicted safety events based upon the Pilot Trial and physiological principles of 25% albumin (ALB) therapy. However, in both the ALIAS Pilot Trial and in ALIAS Part 1, when congestive heart failure (CHF) or pulmonary edema occurred it was generally easily managed with diuretic therapy. These data did not show convincingly that CHF or pulmonary edema were directly associated with early increased mortality, but there were more observed cardiopulmonary adverse events in the ALB group.[11] After an unblinded analysis of the Part 1 safety data, the trial was restarted as ALIAS Part 2 with stringent new inclusion and exclusion criteria and safety monitoring.[12] The Part 2 Trial was stopped early because of futility on the recommendation of the Data Safety and Monitoring Board (DSMB) after 841 (of a planned N = 1100) subjects had been enrolled.[13]

Stroke is associated with secondary and immediate changes in cardiac function. Changes on electrocardiogram, such as T-wave inversion, are commonly associated with hemorrhagic forms of stroke, but may also be seen with ischemic stroke.[14] Troponin elevations in the first hours after ischemic stroke have been documented in up to 20% of patients in recent hospital-based cohorts.[15, 16] This relationship is observed more commonly among patients with hypertension, chronic renal disease, atrial fibrillation, congestive heart failure, and coronary artery disease, but elevated troponins are only variably associated with stroke outcome.[17–19] Similarly, troponin elevations have been documented after carotid artery stenting and are a marker of poorer long-term prognosis.[20] After intracerebral hemorrhage, troponin elevations and minor cardiac events have been documented.[21] Typically, the degree of troponin elevation is small and not associated with either electrocardiographic changes or clinical symptoms such as congestive heart failure.

We report a detailed analysis of the safety results of the ALIAS Part 2 Trial in an attempt to further understand the neutral results of the trial, to explore brain-heart interactions in acute stroke and to examine the role of colloid administration in the acute treatment of stroke.

## Methods

Informed consent for enrolment into the trial was provided by all participants or their legally authorized surrogate decision-maker. The following institutions' IRB (or Research Ethics Board) approved the trial protocol: Arizona—University of Arizona Medical Center—South Campus, Tucson, AZ Arizona—University of Arizona Medical Center, Tucson, AZ; Atlantic Neuroscience Institute, Overlook Hospital Baylor College of Medicine; Buffalo General Medical Center; Centre de Sante et de Service Sociaux de Chicoutimi Centre de recherche de hopital Charles LeMoyne; Chaim Sheba Medical Center at Tel-Hashomer; Cincinnati—Bethesda North Hospital, Cincinnati, OH; Cincinnati—Christ Hospital, Cincinnati, OH; Cincinnati—Good Samaritan Hospital Cincinnati—Mercy Health Fairfield Hospital, Fairfield, OH; Cincinnati—St. Elizabeth Hospital, Florence, KY; Cincinnati—St. Elizabeth Medical Center South, Edgewood, KY; Cincinnati—University Hospital, Cincinnati, OH; Duke University Medical Center; Emory—Emory University Hospital, Atlanta, GA; Emory—Grady Memorial Hospital, Atlanta, GA; Foothills Hospital, University of Calgary; Grey Nuns Community Hospital; HFHS—Henry Ford Hospital, Detroit, MI; Hadassah Medical Organization, Hadassah; University Hospital HealthEast Care System St. Josephs Hospital Helsinki; University Central Hospital Intercoastal Neurology-Intercoastal Medical Research Center Jackson Memorial Hospital, University of Miami; John Muir Medical Ctr-Concord John Muir Medical Ctr-Walnut Creek; Kentucky—University of Kentucky Hospital, Lexington, KY; London Health Sciences Centre-University Hospital; Loyola University Medical Center; Maryland—University of Maryland Medical Center, Baltimore, MD; Maryland—Upper Chesapeake Medical Center, Bel Air, MD; Mayo Clinic Hospital, Phoenix, AZ; Mercy Hospital of Buffalo; MetroHealth Medical Center Minnesota—Fairview Southdale Hospital, Edina, MN; Minnesota—Hennepin County Medical Center, Minneapolis, MN; Minnesota—University of Minnesota Medical Center Fairview, Minneapolis, MN; NYP—NYP Columbia University Medical Center, New York, NY; NYP—New York Methodist Hospital, Brooklyn, NY; NYP—Winthrop University Hospital, Mineola, NY; Neuroscience Research Institute at Florida Hospital Orlando; OHSU—Providence Portland Medical Center; OHSU—Providence St. Vincent Medical Center; OHSU Legacy Emmanuel Hospital; Ohio State University Medical Center; Oregon Health and Science University, Portland; Queen Elizabeth II Health Science Centre; Rambam Health Care Campus; Royal Inland Hospital, Kamloops, BC; Sacred Heart Medical Center Saint Louis University; Seton Medical Center; Soroka Medical Center; Stanford—El Camino Hospital, Mountain View, CA; Stanford—O'Connor Hospital, San Jose, CA; Stanford—Stanford University Medical Center, Palo Alto, CA; Tampere University Hospital Tel-Aviv; Sourasky Medical Center; Temple—Hahnemann University Hospital, Philadelphia, PA; Temple—Penn State Hershey

Medical Center, Hershey, PA; Temple—Temple University Hospital, Philadelphia, PA; Texas—Memorial Hermann Texas Medical Center, Houston, TX; The Ottawa Hospital Thunder Bay Regional Health Sciences Centre; Trillium Health Centre; UCLA-Santa Monica; UCSF—California Pacific Medical Center Davies Campus, San Francisco, CA; UCSF—California Pacific Medical Center Pacific Campus, San Francisco, CA; UCSF—San Francisco General Hospital, San Francisco, CA; UCSF—UCSF Medical Center, San Francisco, CA; UPenn—Abington Memorial Hospital, Abington, PA; UPenn—Christiana Hospital, Newark, DE; University of Alberta; University of Florida/Shands Jacksonville; University of North Carolina at Chapel Hill; University of Pennsylvania Medical Center; University of Pittsburgh Medical Center; University of Toronto, St. Michaels Hospital; VCU—VCU Medical Center, Richmond, VA; Vancouver General Hospital Stroke Program; Villages Research Group Wake Med Health and Hospitals; Wayne—Detroit Receiving Hospital, Detroit, MI; Wayne—Sinai-Grace Hospital, Detroit, MI; Wayne—William Beaumont Hospital, Royal Oak, MI; Wisconsin—Froedtert Memorial Lutheran Hospital, Milwaukee, WI; Yale University School of Medicine, New Haven, CT.

The Albumin in Acute Stroke (ALIAS) Part 2 Trial was a placebo-controlled, double-blind, phase 3 multicenter clinical trial with the primary objective to ascertain whether a weight-adjusted intravenous infusion of 25% albumin solution begun within 5 hours of stroke onset would increase the proportion of subjects with a favorable outcome at 90 days from randomization, compared to subjects treated with a similar volume of isotonic saline, over and above the current best standard of care.[9, 13] A favorable primary outcome was defined as *either* a modified Rankin Scale (mRS) score of 0 or 1 *or* a National Institutes of Health Stroke Scale (NIHSS) score of 0 or 1, *or both*. A centralised web-based randomisation process assigned subjects 1:1 to either albumin or saline via a minimisation-plus-biased-coin algorithm that accounted for the current status of treatment group balance within and across sites. We used multiple methods to ensure the effectiveness of blinding including blinded treatment kits, opaque IV kit covering, and 90-day outcome assessment by a blinded observer.

Adult patients aged 18 through 83 with ischemic stroke and baseline NIHSS score ≥ 6 were randomized and treated within 5 hours of stroke onset. Age 83 years was a data-driven upper limit based upon the ALIAS Part 1 study.[22] Subjects were treated intravenously with 25% human albumin (2 g per kg estimated body weight) infused over 2 hours. Control subjects received a comparable volume of isotonic saline. Vital signs were monitored frequently. The following data were monitored and collected: serum chemistry, ECG and intravenous fluid intake at 24 and 48 hours, brain CT or MRI at 24 hours, neurological and cardiac status including NIHSS at 24 and 48 hours and at 7 days or discharge, whichever came first. Patients with elevated baseline troponin levels or baseline abnormal ECG findings suggesting cardiac ischemia were excluded per protocol. Patients at risk for CHF episode or exacerbation were also excluded—those with CHF in the last 6 months, severe aortic or mitral stenosis, cardiac surgery in the last 6 months, acute myocardial infarction (AMI) in the last 3 months, signs or symptoms of current AMI, any physical exam finding suggestive of CHF, hemodynamically significant arrhythmias, evidence of CHF on chest X-ray (if obtained), creatinine > 2.0 mg/dL or elevated baseline troponin (>0.1 μg/L).

Intravenous fluid management and diuretic administration were mandated. Subjects were not to receive total intravenous fluids in excess of 4200 ml during the first 48 hours, including the volumes of study drug and thrombolytic agent (if used). For subjects exceeding this amount, site investigators were required to provide justifications, which were centrally adjudicated as to clinical reasonableness. Subjects were also required to receive a single dose of furosemide 20 mg IV (or an equivalent loop diuretic), between 12 and 24 hours after study-drug administration. Physicians withholding diuretics were asked to provide a written justification. Antiplatelet therapy was recommended in all subjects within 48 hours of their stroke. Blood pressure was managed according to the local standard of care.

Eligible patients received intravenous tPA within standard time windows according to local guidelines. If available at the site and clinically routine, endovascular treatment (intra-arterial tPA and/or endovascular thrombectomy) was permitted within the trial protocol.

Safety event reports were reviewed internally by the study team blinded to treatment allocation for completeness. All serious adverse events were adjudicated for unexpectedness and relatedness to the study treatment by the site PI and by two external safety monitors who remained blinded to treatment allocation. Adverse events were subsequently reviewed by the unblinded DSMB statistician on a monthly basis and by the trial’s DSMB, initially on a quarterly basis and then every six months. This heightened degree of safety monitoring was implemented because of the results of ALIAS Part 1. ECGs were adjudicated both at the site level and centrally and troponin levels were re-measured at 24–48 hours. Brain imaging was reviewed centrally by a 2-reader panel consisting of a neuroradiologist and neurologist. Scans (primarily CT but some MRI) were scored for baseline stroke severity using the ASPECTS score, and the 24-hour scans for hemorrhage and infarct size.

We defined common stroke-related expected adverse events per protocol. Symptomatic intracranial hemorrhage was defined as a new intracranial hemorrhage causally associated with deterioration in neurological status as determined by the treating physician. It was considered related to acute thrombolytic treatment if it occurred in the first 24 hours after treatment. Pulmonary edema or congestive heart failure was defined as any evidence of pulmonary compromise associated with physical examination signs of fluid overload. We defined pulmonary edema/congestive heart failure at five levels: 1) asymptomatic—subject requires no additional therapy; pulmonary congestion noted on chest X-ray only, 2) mild—subject requires and responds to a single dose of diuretic (20mg intravenous furosemide or equivalent) and requires supplemental oxygen by nasal prongs only, 3) moderate—subject requires more than a single dose of diuretic but responds and requires supplemental oxygen by nasal prongs only, 4) severe—the subject requires treatment with multiple doses of a diuretic, supplemental oxygen

via face mask, or additional problems arise such as cardiac arrhythmias or a requirement for cardiac telemetry, 5) life-threatening—the subject requires intubation or non-invasive BiPAP/CPAP to manage significant hypoxemia. Levels 3–5 were automatically reported as SAEs. Troponin elevation was defined as any troponin level greater than 0.1 μg/L.

Prior to the commencement of the ALIAS Part 2 Trial, we assessed the Virtual International Stroke Trials Archive (VISTA) data set[23] to examine for evidence of congestive heart failure among stroke patients in previous stroke trials. The VISTA data were accessed in June 2006, at which time 5 placebo-controlled trials had adequate reportable information on CHF as an adverse event. Three trials used a saline placebo and 2 used a non-saline placebo. In these trials, the dose of saline or vehicle used was ≤ 500 ml/day in contrast to the ALIAS dose which was up to 750ml of study drug/saline over 2h. We assessed for the occurrence of CHF and evaluated predictors of CHF from the existing data.

### Role of funding source

The trial was funded by the U.S. National Institutes of Health/National Institute of Neurological Disorders and Stroke. A representative of NINDS (CSM) participated in study design, management and interpretation. Baxter Healthcare Corporation provided funds to extend the trial to Finland but played no role in the design, execution, or interpretation of the study.

## Statistical Methods

The safety sample (n = 830) was defined a priori and consisted of all subjects who received at least 20% of the calculated dose of study drug. This differed from the intention-to-treat (ITT) sample by 11 patients. Data are reported using standard descriptive statistics. Adjusted estimates of effect size were produced using generalized linear models log link to directly calculate the rate ratios. In the analysis of the VISTA data, we used multiple logistic regression and effect size estimates are presented as odds ratios. Each test was conducted using two-sided alpha = 0.05. We made no adjustment for multiplicity of testing. All analyses presented are hypothesis-generating.

## Results

841 subjects from U.S. (69 sites), Canada (13 sites), Finland (2 sites), and Israel (5 sites) were randomized to the trial from February 2009 to September 2012. Baseline characteristics were similar between treatment and control arms (Table 1). The median doses were 632 ml (IQR 181ml) albumin, and 640ml (IQR 176ml) 0.9% saline, infused over 2 hours.

**Table 1:** Baseline and Treatment Characteristics of the Subjects in Safety Sample.

	Albumin (N = 416)	Saline Control (N = 414)
Demographics		
Age (mean, SD)	63.5 (12.9)	64.9 (12.9)
Sex, male (%, n)	52.9% (220)	56.0% (232)
Caucasian^†^ (%,n)	74.5% (310)	72.8% (302)
Black^†^ (%, n)	16.1% (67)	20.3% (83)
Asian^†^ (%, n)	6.0% (25)	4.8% (20)
Medical History		
Hypertension	71.9% (299)	70.5% (292)
Atrial fibrillation	18.8% (78)	18.6% (77)
Past congestive heart failure	4.1% (17)	6.3% (26)
Past myocardial infarction	10.6% (44)	12.1% (50)
Past stroke	20.9% (87)	18.6% (77)
Past transient ischemic attack	12.3% (51)	11.8% (49)
Diabetes mellitus	18.5% (77)	22.5% (93)
Hyperlipidemia	46.6% (194)	51.0% (211)
Peripheral vascular disease	5.0% (21)	6.0% (25)
Clinical Factors		
NIHSS score^‡^ (median, iqr)	10 (9)	11 (9)
ASPECTS^§^ > 7 (%, n/N)	77.4% (319/412)	78.0% (323/410)
OCSP** clinical stroke type		
TACS	24.3% (101)	23.4% (97)
PACS	55.3% (230)	56.0% (232)
LACS	12.0% (50)	11.1% (46)
POCS	8.4% (35)	9.4% (39)
Thrombolysis	83.2% (346)	86.0% (356)
Intravenous tPA* only	66.6% (277)	69.8% (289)
Intravenous tPA* plus any endovascular procedure	16.6% (69)	16.2% (67)
Any endovascular procedure only	5.8% (24)	2.9% (12)
No thrombolysis	11.1% (46)	11.1% (46)
Systolic BP^¶^, mm Hg (mean, SD)	155 (28)	157 (30)
Glucose, mmol/L (mean, SD)	7.1 (2.5)	7.7 (3.7)
Hemoglobin, g/L (mean, SD)	139 (17)	140 (18)
Creatinine, μmol/L (mean, SD)	86.3 (23.1)	90.3 (26.9)
ECG^¶^ at baseline shows normal sinus rhythm (%,n)	72.3% (297)	72.9% (296)
Process Measures		
Stroke onset to initiation of study drug infusion (min) [median, iqr]	200 (82)	198 (75)
Stroke onset to initiation of intravenous tPA (min)[median, iqr]	126 (71)	131 (68)
Initiation of IV tPA to initiation of study drug infusion (min) [median, iqr]	60 (31)	60 (32)
Post-treatment–Fluids and Cardiac Status		
Total IV fluids administered within 48 hours of randomization (ml) (mean (SD); [min, max])	3284 (1669); [416,15006]	3249 (1629);[432, 11235]
ECG^¶^ at 24 hours shows normal sinus rhythm (%, n)	70.3% (275)	78.3% (313)

* tPA = tissue plasminogen activator

** OCSP = Oxfordshire Community Stroke Project stroke classification (TACS = total anterior circulation syndrome; PACS = partial anterior circulation syndrome; LACS = lacunar syndrome; POCS = posterior circulation syndrome)

**¶**BP = blood pressure; IV = intravenous; ECG = electrocardiogram

† Race and ethnic group were self-reported.

‡ The National Institutes of Health Stroke Scale (NIHSS) is a 42-point scale that quantifies neurological deficits in 11 categories, with 0 indicating normal function without deficits, and higher scores indicating greater severities of deficit.

§ The Alberta Stroke Program Early Computed Tomography Score (ASPECTS) uses computed tomography to assess 10 regions of the brain; a score of 1 indicates a normal region and 0 indicates a region showing signs of ischemia. Total scores range from 10 (no evidence of early ischemia) to 0 (all 10 regions of the affected hemisphere show early ischemic changes).

Major neurological adverse events occurring in the trial were intracranial hemorrhage, hemicraniectomy, neurological deterioration, and neurological death (Table 2). The rate of symptomatic intracranial hemorrhage was low (2.9%, 24/830) but more common in the ALB-treated subjects (RR = 2.4, CI_95_ 1.01–5.8). Symptomatic ICH occurred in only 1 subject in the non-thrombolysis group. The increase in symptomatic hemorrhage was paralleled by imaging analysis with PH-2 type hemorrhage occurring more commonly in the ALB group (RR = 2.8, CI_95_ 1.01–7.7). Asymptomatic hemorrhage and neurological deterioration were not more common in the ALB group. Hemicraniectomy was uncommon but was numerically more common in the ALB group. Symptomatic ICH was not associated with increased death. Neurological adverse events did not show significant impact on overall primary outcome at 90 days (Table 3).

**Table 2:** Neurological adverse events.

Outcome	Albumin(N = 416)%(n)	Saline Control(N = 414)%(n)	Risk Ratio (CI_95_)
Symptomatic ICH^1^ ANY	6.7 (28)	3.1 (13)	2.1 (1.1–4.1)
Symptomatic ICH^1^ within 24 hours	4.1 (17)	1.7 (7)	2.4 (1.0–5.8)
Asymptomatic ICH^1^ ANY	11.1 (46)	8.7 (36)	1.3 (0.8–1.9)
Asymptomatic ICH^1^ within 24 hours	6.5 (27)	5.6 (23)	1.2 (0.7–2.0)
ICH by Type^2^			
HI-1	12.5 (51)	15.3 (62)	0.8 (0.6–1.2)
HI-2	3.2 (13)	3.5 (14)	0.9 (0.4–1.9)
PH-1	2.0 (8)	1.2 (5)	1.6 (0.5–4.8)
PH-2	3.4 (14)	1.2 (5)	2.8 (1.0–7.7)
rPH	1.2 (5)	1.0 (4)	1.2 (0.3–4.6)
Neurological Deterioration within 48 hours	11.3 (47)	9.7 (40)	1.2 (0.8–1.7)
Hemicraniectomy within 7 days	2.9 (12)	1.5 (6)	2.0 (0.8–5.3)
Neurological Death within 7 days	3.1 (13)	3.1 (13)	1.0 (0.5–2.1)
Neurological Death within 30 days	5.1 (21)	6.0 (25)	0.8 (0.5–1.5)
Neurological Death within 90 days	5.3 (22)	6.3 (26)	0.8 (0.5–1.5)

^1^ICH = intracranial hemorrhage

^2^HI1 = hemorrhagic infarction type 1; HI2 = hemorrhagic infarction type 2; PH1 = parenchymal hematoma type 1; PH2 = parenchymal hematoma type2; rPH = remote parenchymal hematoma

Note: There are 18 subjects without a 24 hour centrally read CT such that the denominator for the Albumin group is 407 and for the Saline group is 405.

**Table 3:** Clinical Outcomes by Adverse Event.

	Primary OutcomeUnadjusted	Primary OutcomeAdjusted*	Death at 90 DaysUnadjusted	Death at 90 DaysAdjusted*
Pulmonary edema/CHF^1^ ANY	1.3 (0.5–2.9)	1.1 (0.5–2.5)	1.0 (0.8–1.3)	1.0 (0.7–1.3)
Pulmonary edema/CHF^1^ within 48 hours	0.8 (0.2–2.5)	0.6 (0.1–1.7)	—-	—-
Acute coronary syndromes ANY	0.9 (0.2–2.9)	0.9 (0.2–2.8)	1.0 (0.6–1.5)	1.0 (0.7–1.3)
Acute coronary syndromes Within 48 hours	1.2 (0.1–8.4)	0.6 (0.08–4.1)	1.1 (0.5–2.0)	1.0 (0.6–1.5)
Troponin leak ANY^1^	0.8 (0.3–1.8)	0.7 (0.3–1.6)	1.0 (0.7–1.3)	1.0 (0.7–1.2)
Troponin leak Within 48 hours	0.7 (0.3–1.5)	0.6 (0.3–1.4)	1.0 (0.7–1.3)	1.0 (0.7–1.3)
Atrial fibrillation ANY	***	***	1.1 (0.9–1.3)	1.0 (0.8–1.2)
Atrial fibrillation within 48 hours	2.4 (0.7–7.4)	2.1 (0.7–6.3)	1.0 (0.8–1.2)	1.0 (0.7–1.3)
Pneumonia ANY	1.7 (0.3–8.1)	1.8 (0.3–8.6)	1.0 (0.7–1.3)	1.0 (0.8–1.2)
Pneumonia within 7 days	2.2 (0.2–18.6)	2.3 (0.2–18.7)	1.1 (0.8–1.5)	1.0 (0.8–1.3)
Shortness of breath ANY	2.0 (0.5–7.5)	1.3 (0.3–4.7)	—-	—-
Shortness of breath within 7 days	—-	—-	—-	—-
Pneumonia or shortness of breath ANY	1.8 (0.6–5.3)	1.8 (0.6–5.3)	1.0 (0.8–1.2)	1.0 (0.8–1.2)
Pneumonia or shortness of breath within 7 days	4.1 (0.5–30.7)	3.9 (0.5–29.3)	1.1 (0.8–1.4)	1.0 (0.8–1.2)
Pulmonary embolus ANY	—-	—-	—-	—-
Pulmonary embolus within 7 days	—-	—-	—-	—-
Symptomatic ICH^2^ ANY	0.5 (0.1–2.0)	0.4 (0.09–1.7)	0.8 (0.5–1.3)	0.9 (0.7–1.2)
Symptomatic ICH^2^ within 24 hours	0.2 (0.02–1.9)	0.2 (0.02–1.9)	0.9 (0.4–2.0)	1.0 (0.6–1.4)
Asymptomatic ICH^2^ ANY	1.0 (0.5–2.0)	1.1 (0.5–2.1)	***	0.9 (0.6–1.3)
Asymptomatic ICH^2^ within 24 hours	1.1 (0.4–2.8)	1.0 (0.4–2.4)	—-	—-
Central Imaging ICH^2^ by Type				
HI-1^3^	—-	—-	1.0 (0.8–1.2)	1.0 (0.8–1.1)
HI-2^3^	0.7 (0.1–3.6)	0.7 (0.1–3.3)	0.9 (0.6–1.2)	0.9 (0.6–1.3)
PH-1^3^	1.3 (0.1–10.5)	1.0 (0.1–8.1)	1.5 (0.6–3.1)	1.1 (0.6–1.9)
PH-2^3^	0.4 (0.02–4.7)	0.4 (0.03–5.2)	1.6 (0.5–5.0)	1.2 (0.7–1.9)
rPH^3^	1.6 (0.2–11.9)	2.0 (0.2–13.8)	—-	—-
Neurological deterioration within 48 hours	0.9 (0.05–13.2)	0.8 (0.05–12.5)	1.0 (0.7–1.4)	1.0 (0.8–1.2)
Hemicraniectomy within 7 days	—-	—-	0.9 (0.5–1.5)	0.9 (0.6–1.4)

^1^CHF = congestive heart failure

^2^ICH = intracranial hemorrhage

^3^HI1 = hemorrhagic infarction type 1; HI2 = hemorrhagic infarction type 2; PH1 = parenchymal hematoma type 1; PH2 = parenchymal hematoma type2; rPH = remote parenchymal hematoma–according to the ECASS-3 classification of hemorrhage[28]

*Adjusted for age, sex, baseline NIHSS score

Cardiopulmonary adverse events were more common in the ALB arm (Table 4). Any congestive heart failure or pulmonary edema in the first 48 hours was 10-fold more likely. The absolute rate (13.0%) was identical to that observed in the ALIAS Pilot Trial (13.4%) and in ALIAS Part 1 (12.1%).[8, 22] In the saline group, congestive heart failure or pulmonary edema occurred in 1.2% of patients. As in the prior ALB trials, CHF or pulmonary edema was generally easily managed with diuretics without the need for more invasive measures such as intubation or intensive care unit admission. We also observed greater proportions of atrial fibrillation and troponin elevation within 48 hours. Troponin elevation without ECG change or cardiac symptoms occurred in 52 subjects (12.5%), of whom 21 developed pulmonary edema or CHF. Acute coronary syndromes occurred infrequently and were associated with troponin elevation. Symptomatic ICH was also associated with a greater occurrence of troponin elevation, atrial fibrillation, pneumonia, and pulmonary embolus but not acute coronary syndromes (S1 Table).

**Table 4:** Cardiopulmonary adverse events.

Outcome	Albumin (N = 416) %(n)	Saline Control (N = 414) %(n)	Risk Ratio (CI_95_)	Prolonged hospital stay or operative intervention due to AE %(n/N)
Pulmonary edema/CHF* ANY	14.4 (60)	4.6 (19)	3.1 (1.9–5.2)	18% (14/79)
Pulmonary edema/CHF* within 48 hours	13.0 (54)	1.2 (5)	10.7 (4.3–26.6)	19% (11/59)
Acute coronary syndromes ANY	4.1 (17)	2.2 (9)	1.9 (0.8–4.2)	46% (12/26)
Acute coronary syndromes Within 48 hours	2.4 (10)	1.0 (4)	2.5 (0.8–7.9)	21% (3/14)
Troponin leak ANY	12.5 (52)	4.6 (19)	2.7 (1.6–4.5)	17% (12/71)
Troponin leak within 48 hours	12.3 (51)	3.9 (16)	3.2 (1.8–5.5)	16% (11/67)
Atrial fibrillation ANY	12.0 (50)	8.5 (35)	1.4 (0.9–2.1)	8% (7/85)
Atrial fibrillation within 48 hours	7.7 (32)	4.6 (19)	1.7 (0.9–2.9)	7% (3/41)
Pneumonia ANY	11.1 (46)	7.5 (31)	1.5 (0.9–2.3)	23% (18/77)
Pneumonia within 7 days	8.2 (34)	4.6 (19)	1.8 (1.0–3.1)	23% (12/53)
Shortness of breath ANY	2.9 (12)	1.9 (8)	1.5 (0.6–3.6)	25% (4/20)
Shortness of breath within 7 days	2.2 (9)	0.7 (3)	3.0 (0.8–10.9)	17% (2/12)
Pneumonia or shortness of breath ANY	13.7 (57)	9.2 (38)	1.5 (1.0–2.2)	23% (22/95)
Pneumonia or shortness of breath within 7 days	10.3 (43)	5.3 (22)	1.9 (1.2–3.2)	21% (14/65)
Pulmonary embolus ANY	1.7 (7)	1.2 (5)	1.4 (0.4–4.4)	33% (4/12)
Pulmonary embolus within 7 days	0.5 (2)	0.0 (0)	∞	50% (1/2)

*CHF = congestive heart failure

Cardiopulmonary adverse events were associated with a substantial need for intensive care admission, prolongation of hospital stay and/or operative intervention (Table 4). This was governed by the adverse event and was not differentially distributed by ALB vs. saline control treatment. No cardiopulmonary adverse events were convincingly associated with the primary outcome or with increased death (Table 3). Symptomatic ICH was numerically associated with poorer outcome, and the direction of effect of cardiopulmonary adverse effects was similarly towards a poorer outcome, but the confidence intervals were broad.

In the VISTA data set of 4,484 patients, median age was 72 (IQR 16) and 47.5% were female. (S2 Table). The majority of patients suffered ischemic stroke (89.5%) with a smaller proportion of ICH (9.4%) and other/non-ischemic (1.1%) final diagnoses. All patients had an onset-to-treatment time of within 12 hours and had been randomized to and received placebo or control. A total of 2,794 (62%) of patients received a saline control. In 895 patients who received saline control, the amount received was 120 ml acutely with standard-of-care intravenous fluids over the next 5 days; in the remainder the total dose is not known. In the non-saline control group (n = 1690, 38%), all patients received 250 ml loading over 30 minutes followed by 250 ml over 3.5 hours and a daily dose of 150 ml for 5 days. An episode of any congestive heart failure occurred in 52 (1.1% CI_95_ 0.9–1.5) patients. Of these 52, data on the timing of CHF were available in 34, and among these 34, 26 (76%) occurred during the first week after enrolment.

Multivariable analysis using the complete VISTA data set showed that age (OR 1.1 per year CI_95_ 1.01–1.1), hypertension (OR 3.6, CI_95_ 1.5–8.5) and saline administration (OR 5.4, CI_95_ 2.1–13.7) were predictive of CHF. Only about half the population had available baseli**n**e NIHSS scores available. In a model using patients with NIHSS data, multivariable analysis of this population showed that stroke severity defined by NIHSS (OR 1.1 per point increase, CI_95_ 1.01–1.10), age (OR 1.1 per year, CI_95_ 1.01–1.1) and saline treatment (OR 10.3, CI_95_ 3.9–27.3) were predictors of CHF. No significant interactions were observed in either model.

## Discussion

Some adverse events, neurological and cardiopulmonary, were more common in the ALB group compared to saline control. None of these adverse events was substantively associated with the primary outcome at 90 days in the ALIAS Part 2 Trial.

The increase in symptomatic ICH, although small, was entirely unexpected. A priori, we had hypothesized the ALB would be protective against ICH because of its potential role as a microcirculation reperfusion agent, blood-brain barrier protectant and anti-oxidant.[24] The increase may be associated with effects on the coagulation system. Hydroxyl-ethyl starch and albumin are associated with a mild coagulopathy, possibly related to hemodilution of coagulation factors and by binding of calcium.[25, 26] Albumin causes smaller ex-vivo clot formation and less stable clots.[26, 27] Thus, it is plausible that high-dose albumin is a mild antithrombotic agent.

Pulmonary edema/congestive heart failure was an expected consequence of ALB therapy and occurred at the same absolute rate as predicted by the ALIAS Pilot Trial and ALIAS Part 1 Trial. Albumin administration most likely resulted directly in intravascular volume expansion, increased pre-load and increased myocardial wall-stress. In predisposed patients, possibly those with pre-existing coronary artery disease or reduced cardiac function, this resulted in an increased incidence of cardiopulmonary adverse events. However, the large majority of occurrences of fluid overload manifesting as congestive heart failure and pulmonary edema were easily managed with diuretics and fluid restriction in the first 48 hours.

In addition, symptomatic ICH was associated with troponin elevations. Because we excluded patients with baseline troponin elevations [1.2% (10/830) had baseline troponin elevation > 0.1 μg/L], we likely selected a group of stroke patients at lowest risk for cardiac complications. Prior studies have suggested that patients with elevated troponin levels at baseline have a higher prevalence of co-morbid cardiac conditions, most commonly atrial fibrillation. Nevertheless, the increase in troponin that occurred in association with ICH is concordant with other studies showing a link between stroke and sub-clinical cardiac ischemia. We did not observe a relative increase in the frequency of ICH over the course of the trial, but the increased occurrence of ICH in the ALB group is a component cause of the overall increase in cardiopulmonary adverse events in the trial.

If we assume that infusion of an average of 640 ml of saline over 2 hours can be considered a standard treatment and examine the control group, then the occurrence of cardiopulmonary events in ischemic stroke patients is not trivial. Between 1 and 5% of patients have a cardiopulmonary outcome in the first 48 hours and nearly double that over 90 days. The analysis of the VISTA data set adds further credence to the observation that CHF is not a rare event after stroke. Further, we present the intriguing possibility that treatment with saline is not necessarily an inactive placebo. In the VISTA data set, we provide evidence that saline administration was associated with the development of CHF as an adverse event. Nevertheless, it is clear that the oncotic effect of 25% albumin resulted in a greater degree of fluid overload compared to saline alone. It remains possible that adverse events counterbalanced any potential treatment effect, such that the overall effect of ALB treatment was neutral.

Overall, cardiopulmonary events are relatively common among ischemic stroke patients. Our study is likely to have underestimated the common population rate of such events because we specifically excluded patients with high-risk cardiac status. The ALIAS-2 Trial adds to the existing literature on the brain-heart interaction in the acute phase of illness.

## Supporting Information

S1 ALIAS Enrolling Site Ethics Board List (XLSX)

S1 CONSORT Checklist CONSORT Trial checklist. (PDF)

S1 Protocol ALIAS Protocol. (PDF)

S1 Table Adverse Events among patients with sICH vs. non-ICH patients. (DOCX)

S2 Table VISTA data population. (DOCX)
