# Supplementary material for: Albumin Administration in Acute Ischemic Stroke: Safety Analysis of the ALIAS Part 2 Multicenter Trial
Source: PLoS One. 2015 Sep 1;10(9):e0131390. doi: 10.1371/journal.pone.0131390 (PMC4556660; doi:10.1371/journal.pone.0131390)
Supplement: S1 Protocol — (PDF) [file pone.0131390.s003.pdf]

*(VERSION 4.01: September 22, 2008)*

## Clinical Study Protocol

# Albumin in Acute Stroke (ALIAS) Trial-Part 2:

## A Phase III Randomized Multicenter Clinical Trial of High-Dose Human Albumin Therapy for Neuroprotection in Acute Ischemic Stroke


**Study Chair:**

Myron D. Ginsberg, M.D.
Peritz Scheinberg Professor of Neurology
University of Miami School of Medicine

**Canadian Co-PI**

Michael D. Hill, M.D., M.Sc., FRCPC
Associate Professor, Calgary Stroke Program

**Biostatistics Co-PI**

Yuko Y. Palesch, Ph.D.
Professor of Biostatistics
Medical University of South Carolina



**Supported by:**

**The National Institute of Neurological Disorders and Stroke (NINDS)**

**U01 NS40406**
**U01 NS054630**

**Sponsor of IND:**

**Myron D. Ginsberg, M.D.**
**BB-IND #: 12817**

# TABLE OF CONTENTS

Page

ABBREVIATIONS ..... iii

PROTOCOL SYNOPSIS..... v

TRIAL ADMINISTRATIVE ORGANIZATION ..... 1

1. STUDY OBJECTIVES..... 4
   - 1.1 Primary Objective ..... 4
   - 1.2 Secondary Objectives ..... 4

2. BACKGROUND ..... 4
   - 2.1 Rationale ..... 4
   - 2.2 Supporting Clinical Data ..... 8
   - 2.3 Summary of Background and Rationale..... 13

3. STUDY DESIGN ..... 14

4. SELECTION AND ENROLLMENT OF SUBJECTS..... 14
   - 4.1 Inclusion Criteria ..... 14
   - 4.2 Exclusion Criteria ..... 15
   - 4.3 Study Enrollment Procedures ..... 16

5. STUDY INTERVENTIONS ..... 21
   - 5.1 Interventions, Administration, and Duration ..... 21
   - 5.2 Handling of Study Interventions ..... 22
   - 5.3 Concomitant Interventions ..... 23

6. CLINICAL AND LABORATORY EVALUATIONS ..... 27
   - 6.1 Data-Collection Schedule ..... 27
   - 6.2 Timing of Evaluations..... 27
   - 6.3 Off-Intervention Requirements ..... 29

7. CLINICAL MANAGEMENT OF ADVERSE EXPERIENCES ..... 29
   - 7.1 Complications of ALB Therapy ..... 29
   - 7.2 Complications of Thrombolytic Therapy..... 30

8. ADVERSE EVENTS REPORTING ....31

8.1 Definition of Adverse Events....31
8.2 Formal Definitions of Selected Adverse Events....32
8.3 Definition of Intracranial Hemorrhage (ICH)....35
8.4 Reporting of Adverse Events and Serious Adverse Events....36
8.5 Review and Expedited Reporting of Serious Adverse Events....36
8.6 Follow Up Reporting of Serious Adverse Events....37
8.7 Additional Safety Review....37
8.8 Monitoring of Specific Adverse Events....39

9. CRITERIA FOR INTERVENTION DISCONTINUATION....39

10. STATISTICAL CONSIDERATIONS ....40

10.1 Overview....40
10.2 Outcomes ....40
10.3 Sample Size and Accrual ....41
10.4 Data Analyses ....41
10.5 Analysis Samples....43
10.6 Handling Missing Data....44
10.7 Data Monitoring....44

11. DATA COLLECTION AND MANAGEMENT OVERVIEW ....47

12. HUMAN SUBJECTS ....48

12.1 Institutional Review Board (IRB)/Research Ethics Board (REB) Review and Informed Consent ....48
12.2 Subject Confidentiality ....48
12.3 Study Modification/Discontinuation ....48
12.4 Site Monitoring....49

13. PUBLICATION AND PRESENTATION POLICY....50

14. ANCILLARY STUDIES POLICY....51

15. DATA-SHARING PLAN....51

16. REFERENCES ....52

## ABBREVIATIONS

| | |
|---|---|
| ACCP | American College of Chest Physicians |
| AE | Adverse Event |
| ALB | Human Albumin |
| AMMs | ALIAS Medical Monitors |
| ASA | Acetylsalicylic Acid |
| ASPECTS | Alberta Stroke Programme Early CT Score |
| BI | Barthel Index |
| CBC | Complete Blood Count |
| CHF | Congestive Heart Failure |
| CRFs | Case Report Forms |
| CCC | Canadian Coordinating Center (at the University of Calgary) |
| CSC | Canadian Stroke Consortium |
| CT | Computed Tomography |
| CXR | Chest X-ray |
| DCU | Data Coordination Unit (at the Medical University of South Carolina) |
| DHHS-SSC | Department of Health and Human Services Supply Services Center |
| DSMB | Data and Safety Monitoring Board |
| EKG | Electrocardiogram |
| EMT | Emergency Medical Transportation |
| ED | Emergency Department (Emergency Room) |
| EuroQol | A standardized assessment instrument (developed by the EuroQol Group) that provides a simple descriptive measure of health outcome |
| FDA | Food and Drug Administration |
| HIPAA | Health Insurance Portability and Accountability Act |
| IA | Intra-arterial |
| ICH | Intracranial Hemorrhage |
| ICH-GCP | International Conference on Harmonization Good Clinical Practice |
| ICP | Intracranial Pressure |
| ICU | Intensive Care Unit |
| INR | International Normalized Ratio |
| IRB | Institutional Review Board |
| ITT | Intent to Treat |
| IV | Intravenous |
| JVP | Jugular Venous Pulse |
| MI | Myocardial Infarction |
| MRI | Magnetic Resonance Imaging |
| MoP | Manual of Procedures |
| mRS | Modified Rankin Scale |
| MSM | Medical Safety Monitor |
| MUSC | Medical University of South Carolina |
| NIHSS | National Institutes of Health Stroke Scale |
| NINDS | National Institute of Neurological Disorders and Stroke |
| PIPED | Personal Information and Portable Electronic Documents Act |
| QVSFS | Questionnaire to Validate Stroke-Free Status |

| | |
|---|---|
| REB | Research Ethics Board |
| SAE | Serious Adverse Event |
| SAH | Subarachnoid Hemorrhage |
| SCO | Study Chair Office |
| SDH | Subdural Hematoma |
| SSQOL | Stroke-Specific Quality of Life |
| tPA | Tissue Plasminogen Activator |
| TCD | Transcranial Doppler |

# ALIAS TRIAL PART 2 PROTOCOL SYNOPSIS

| | |
|---|---|
| **Protocol Title** | Albumin in Acute Stroke Trial: A Phase III Randomized Multicenter Clinical Trial of High-Dose Human Albumin Therapy for Neuroprotection in Acute Ischemic Stroke |
| **Acronym** | ALIAS |
| **Clinical Trial Phase** | Phase III |
| **Study Sites** | • University of Miami (Study Chair Site and Fiscal Management Office)<br>• DCU at MUSC (Data and Project Management and Statistics Center)<br>• University of Calgary (Canadian Coordinating Center)<br>• Approximately 100 clinical centers in US, including sites from the Neurological Emergencies Treatment Trials (NETT) Network, Canada, and possibly other countries |
| **Study Period** | Planned enrollment period – 3 years<br>Planned duration of the study – 4 years |
| **Study Population** | Acute ischemic stroke patients. |
| **Primary Study Objective** | To ascertain whether high-dose human albumin (ALB) therapy confers neuroprotection in acute ischemic stroke over and above best standard of care. (Specifically, to determine whether ALB therapy increases the proportion of acute ischemic stroke patients with favorable outcome compared to placebo therapy at 3 months from randomization.) |
| **Secondary Study Objectives** | To evaluate:<br>• overall clinical outcome (as assessed by the global statistical test of NIHSS, mRS, and BI scores) at 3 months post-randomization.<br>• overall clinical outcome as assessed by the full scale of the modified Rankin scale.<br>• neurological outcome as assessed by NIHSS score at 3 months post-randomization.<br>• functional outcome as assessed by mRS and BI at 3 months post-randomization.<br>• quality-of-life as assessed by EuroQol at 3 months and 1 year post-randomization, and by Stroke-Specific Quality of Life (SSQOL) instruments at 3 months post-randomization.<br>• robustness of ALB therapy as measured by a favorable |

| | |
|---|---|
| | outcome of mRS of 0 or 1at one year post-randomization.<br>• incidence of recurrent ischemic stroke within 1 month, 3 months and 1 year post-randomization, as assessed by Questionnaire to Validate Stroke-Free Status (QVSFS).<br>• mortality within 3 months and 1 year post-randomization.<br>• incidence of symptomatic ICH within 24 (± 6) hours of randomization.<br>• cognition measured at 3 months by Trailmaking A and B. |
| **Study Design** | The ALIAS Trial consists of a multicenter, randomized, double-blind, parallel group, two-arm Phase III trial of ALB therapy in patients with acute ischemic stroke. |
| **Sample Size** | Approximately 1100 subjects are randomized in a 1:1 ratio to IV treatment of either ALB or placebo saline solution. |
| **Inclusion Criteria** | • Acute ischemic stroke<br>• Age 18 years through 83 years (have not had their $84^{th}$ birthday).<br>• NIHSS score of 6 or greater as assessed immediately prior to thrombolysis treatment if the patient is eligible for thrombolysis or immediately prior to randomization for patients not eligible for thrombolysis.<br>• Initiation of ALB/placebo within 5 hours of stroke onset, and within 60 minutes of the start of the thrombolysis infusion, if IV thrombolysis is given.<br>• Signed and dated informed consent has been obtained. |
| **Exclusion Criteria** | • Episode/exacerbation of congestive heart failure (CHF) from any cause in the last 6 months. (An episode of congestive heart failure is any heart failure that required a change in medication, change in diet or hospitalization.)<br>• Known valvular heart disease with CHF in the last 6 months.<br>• Known (or in the Investigator's clinical judgment) existence of severe aortic stenosis or mitral stenosis.<br>• Cardiac surgery involving thoracotomy (e.g., coronary artery bypass graft (CABG), valve replacement surgery) in the last 6 months.<br>• Acute myocardial infarction in the last 6 months.<br>• Signs or symptoms of acute myocardial infarction, including EKG findings, on admission.<br>• Elevated serum troponin level on admission (> 0.1 mcg/L)<br>• Suspicion of aortic dissection on admission.<br>• Acute arrhythmia (including any tachy- or bradycardia) *with* |

| | |
|---|---|
| | hemodynamic instability on admission (systolic blood pressure < 100 mmHg).<br>• Findings on physical examination of *any* of the following: (1) jugular venous distention (JVP > 4 cm above the sternal angle); (2) 3rd heart sound; (3) resting tachycardia (heart rate > 100/min) attributable to congestive heart failure; (4) abnormal hepatojugular reflux; (5) lower extremity pitting edema attributable to congestive heart failure; (6) bilateral rales; and/or (7) if a chest x-ray is performed, definite evidence of pulmonary edema, bilateral pleural effusion, or pulmonary vascular redistribution<br>• Current acute or chronic lung disease requiring supplemental chronic or intermittent oxygen therapy.<br>• Historical modified Rankin Scale (mRS) $\geq$2. Patients who live in a nursing home or who are not fully independent for activities of daily living (toileting, dressing, eating, cooking and preparing meals, etc.), immediately prior to the stroke are not eligible for the trial.<br>• In-patient stroke. Patients with stroke occurring as a complication of hospitalization for another condition, or as a complication of a procedure.<br>• Profound dehydration.<br>• Fever, defined as core body temperature > 37.5°C (99.5°F).<br>• Serum creatinine > 2.0 mg/dL or 180 μmol/L.<br>• Severe chronic anemia (hemoglobin < 7.5 g/dL or 75g/L).<br>• Evidence of intracranial hemorrhage (intracerebral hematoma, intraventricular hemorrhage, subarachnoid hemorrhage (SAH), epidural hemorrhage, acute *or* chronic subdural hematoma (SDH)) on the baseline CT or MRI scan.<br>• History of or known allergy to albumin.<br>• History of or known allergy to natural rubber latex.<br>• Pregnancy, breastfeeding or positive pregnancy test. (Women of childbearing age must have a negative pregnancy test prior to study drug administration.)<br>• Concurrent participation in any other therapeutic clinical trial.<br>• Evidence of any other major life-threatening or serious medical condition that would prevent completion of the study protocol, impair the assessment of outcome, or in which ALB therapy would be contraindicated or might cause harm to the subject. |
| **Study Intervention and Follow-up** | Upon randomization, each subject receives infusion of either ALB or isotonic saline solution over the course of 2 hours. The |

| | |
|---|---|
| | total dose of 2.0 g/kg is based on the subject's estimated weight at the time of randomization and must not exceed 750 ml. The study drug infusion must start within 60 minutes of the start of the thrombolysis infusion (if thrombolysis is given*) and within 60 minutes of randomization for the non-thrombolysis stratum It must be administered through a dedicated IV line.<br>[* In subjects fully eligible for the ALIAS Trial pending receipt of serum troponin level, an interval of up to 90 minutes between start of thrombolysis or randomization is permitted.]<br>Each subject is monitored closely throughout his/her hospitalization for the qualifying stroke and is followed for one year from randomization. The primary efficacy outcome is assessed at 3 months from randomization at a clinic visit. Each subject is contacted by telephone for brief (<30 minutes) clinical and quality-of-life assessments at 1, 6, 9 and 12 months from randomization. |
| **Primary Outcome Measure** | The primary outcome is the favorable outcome, defined as *either* an NIH Stroke Scale (NIHSS) score of 0 or 1, *or* a modified Rankin Score (mRS) of 0 or 1, *or* both, measured at 3 months from randomization. |
| **Statistical Analysis for Primary Outcome Measure** | The primary hypothesis of the ALIAS Trial is tested using the generalized linear model with log link function, with adjustment for thrombolysis stratum and baseline NIHSS score. Subsequently, secondary analyses are conducted adjusting for pertinent factors, such as clinical site, age, and NIHSS score at baseline. |

# TRIAL ADMINISTRATIVE ORGANIZATION

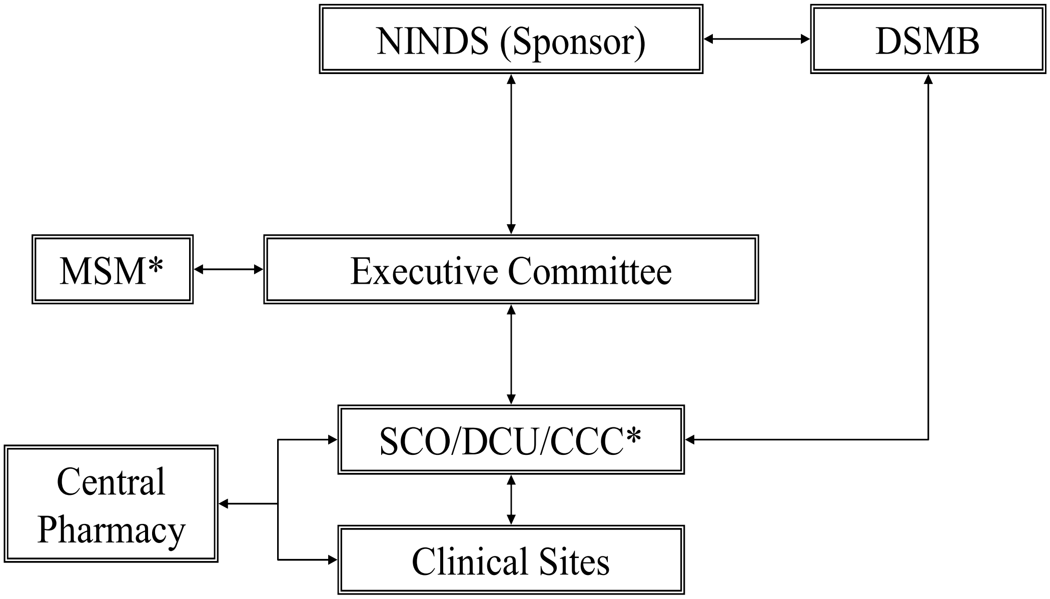


* SCO= Study Chair Office; DCU=Data Coordination Unit; CCC=Canadian Coordinating Center; MSM=Medical Safety Monitor

## Executive Committee

The Executive Committee consists of the Study Chair, the Clinical Project Coordinator, and the Financial Manager at the University of Miami; the Director of the DCU, the Data Manager, the Project Manager, and the Project Management Assistant (Ex Officio) at MUSC; the Director of the CCC and the Study Coordinator at the University of Calgary; and a NINDS appointed liaison. The Committee is a working group responsible for the development and amendment of the study documents (e.g., Protocol, Case Report Forms, and Manual of Operations); collection, review and oversight of dissemination of SAE occurrences and other important events pertinent to the study; and communication among all components of the study participants (e.g., SCO, DCU, CCC, clinical sites, MSMs, and NINDS). In addition, the Study Chair and the Director of the CCC serve as internal medical monitors (ALIAS Medical Monitors - AMMs).

**Study Chair Office (SCO)**

The Study Chair Office, housed in the Department of Neurology at the University of Miami Miller School of Medicine, provides overall scientific coordination and fiscal management of the ALIAS Trial and is responsible for preparing progress reports and grant renewal applications. The SCO comprises the ALIAS Trial's Principal Investigator, the Clinical Project Coordinator, and the ALIAS Trial's Financial Manager. The Principal Investigator (PI) provides overall leadership to the entire ALIAS Trial to ensure its successful implementation. He visits all U.S. clinical sites on a periodic basis and collaborates with the DCU in organizing all necessary meetings. As the Sponsor of the Investigational New Drug (IND) application, he ensures that the Trial is conducted according to Good Clinical Practice (GCP) guidelines and FDA and NIH regulations. The Clinical Project Coordinator assists the PI in day-to-day implementation of the Trial and serves as a major contact person for investigators and study coordinators at U.S. sites. The Financial Manager, together with the PI, is responsible for the budgetary management of NIH Grant U01-NS40406, which funds the Miami SCO, the CCC in Calgary, and all U.S. and Canadian clinical sites. These responsibilities include preparation of consortium agreements and subcontracts, handling of invoices and purchase orders, and directing disbursement of funds. The Financial Manager interacts closely with the DCU.

**Data Coordination Unit (DCU)**

The Data Coordination Unit (DCU) is housed in the Department of Biostatistics, Bioinformatics and Epidemiology ($DB^2E$) at MUSC in Charleston, SC. The responsibilities of the DCU include data processing and management of data obtained at all study sites (U.S. and Canadian), project management, coordination and communication of Trial activities (e.g., meeting and teleconference arrangements), statistical analyses, and generation and distribution of progress reports, reports to the DSMB, and newsletters. The DCU enlists assistance from the Canadian Coordinating Center (CCC) in its liaison with Health Canada.

**Canadian Coordinating Center (CCC)**

The Canadian Coordinating Center, which is located in the Department of Clinical Neurosciences at the University of Calgary / Foothills Medical Centre, is responsible for the coordination and oversight of the investigators and study coordinators at the Canadian sites regarding Canadian regulatory affairs. The Canadian Coordinating Center will also function as the central review site for all neuro-imaging and EKGs.

**Medical Safety Monitors (MSMs)**

The MSMs are experienced stroke neurologists not affiliated with any of the institutions participating in the ALIAS Trial. The MSMs' responsibilities are to review all SAEs and determine whether they are possibly related to the study drug administration, and to adjudicate adverse outcome events (e.g., recurrent stroke, MI, symptomatic ICH). Originally, the MSMs are blinded with regard to treat-

ment assignment of SAE cases; however, if the DSMB requests that they be unblinded, their communications, if any, with members of the ALIAS Executive Committee will be conducted to preserve the blinded nature of the Executive Committee members.

## Data and Safety Monitoring Board (DSMB)

The Data and Safety Monitoring Board (DSMB) is appointed by the NINDS Director and managed by the NINDS Clinical Trials group. Its overarching responsibility is the oversight of safety of the Trial participants. They review reports on SAEs, request additional data/information, if necessary, and must be cognizant of external new information regarding the safety of ALB treatment. Upon review of periodic data, they advise the NINDS regarding continuation of the Trial.

# 1. STUDY OBJECTIVES

## 1.1 Primary Objective

The primary aim is to ascertain whether high-dose ALB therapy confers neuroprotection in acute ischemic stroke over and above best standard of care. Specifically, we wish to determine whether ALB therapy increases the proportion of acute ischemic stroke patients with a favorable outcome compared to placebo therapy. Favorable outcome is defined as *either* an NIH Stroke Scale (NIHSS) score of 0 or 1, *or* a modified Rankin Score (mRS) of 0 or 1, *or* both, measured at 3 months from randomization.

## 1.2 Secondary Objectives

Secondary aims are to compare ALB therapy to placebo with respect to the following:

- overall clinical outcome (as assessed by the global statistical test of NIHSS, mRS, and Barthel Index scores) at 3 months post-randomization.
- overall clinical outcome as assessed by the full scale of the modified Rankin scale.
- neurological outcome as assessed by NIHSS score at 3 months post-randomization.
- functional outcome as assessed by mRS and by Barthel Index at 3 months post randomization.
- quality-of-life as assessed by EuroQol at 3 months and 1 year post-randomization, and by Stroke-Specific Quality of Life (SSQOL) instruments at 3 months post-randomization.
- robustness of ALB therapy as measured by a favorable outcome of mRS of 0 or 1 at one year post-randomization.
- incidence of recurrent ischemic stroke within 1 month, 3 months and 1 year post-randomization, as assessed by Questionnaire to Validate Stroke-Free Status (QVSFS).
- incidence of symptomatic ICH in the thrombolysis stratum within 24 ($\pm$6) hours of randomization.
- mortality within 3 months and within 1 year post-randomization.
- cognition measured at 3 months by Trailmaking A and B.

# 2. BACKGROUND

## 2.1 Rationale

### 2.1.1 The Unmet Need for Neuroprotective Stroke Therapies

In the field of clinical stroke management, there is a compelling, unmet need for safe and effective neuroprotective strategies to limit brain injury, facilitate brain repair, and improve functional outcome. Stroke is the third leading cause of death in North America and the chief cause of chronic disability: more than 750,000 individuals continue to suffer an acute stroke each year [112], and there are as many as 4.8 million chronic stroke survivors [82,101]. The economic impact on our health care system for acute and chronic stroke care is estimated to exceed $50 billion annually, and the overall burden of chronic disability is inestimable.

### 2.1.2 Neurotherapeutics for Ischemic Stroke

Multiple pathways and cascades of electrophysiological, biochemical, and molecular events interact to cause the death of brain cells, and many potential neuroprotective strategies have been explored (for representative reviews, see [20,28,30,35,48,57,86,94,117]). Importantly, a large number of experimental studies have now conclusively established that, by intervening promptly, it is possible to achieve substantial protection of ischemically threatened brain tissue. Intravenous tissue plasminogen activator (tPA) is beneficial in hyperacute ischemic stroke [71] but, unfortunately, is being applied to only 2-3% of overall patients owing to both practical logistics as well as reluctant physician acceptance due to lingering concerns over the complication of intracranial hemorrhage. Many pharmacological strategies to protect the acutely ischemic brain have shown efficacy in preclinical studies in animals [35], but their translation to the clinic has proven challenging [4,31,39,57,66,95]. To date, clinical neuroprotection trials have only rarely satisfied all of the following criteria: 1) use of agents that showed robust efficacy in relevant preclinical models; 2) use of agents with benign side-effect profiles; 3) use of clinical doses corresponding to those shown to be effective in animal studies; 4) initiation of treatment within the rather narrow therapeutic window required for neuroprotection; and 5) robust clinical-trial design (i.e., adequately powered for clinically reasonable effect-size, use of appropriate clinical-outcome instruments and statistical methods) [37,39].

### 2.1.3 High-Grade Neuroprotection with Human Albumin Therapy – Preclinical Studies

An ideal neuroprotective agent would be one that 1) exhibits proven efficacy; 2) carries minimal risk of adverse effects; 3) is acceptable both to medical personnel and to patients and their families; and 4) can be administered without the need for complicated laboratory studies or sophisticated delivery systems. In extensive studies (both preclinical and clinical) conducted over the past several years, human albumin therapy has emerged as a highly promising agent of this type. We have shown that moderate- to high-dose human albumin therapy is highly neuroprotective in animal models of both temporary [12,13,17] and permanent [65] focal cerebral ischemia; as well as in global cerebral ischemia [16] and traumatic brain injury [11]. In ischemia, albumin (dose, 1.25 g/kg i.v.) diminished total infarct volume by two-thirds and reduced brain edema by three-quarters or more, with a therapeutic window of efficacy extending to four hours [13]. In a comprehensive meta-analysis of our focal ischemia data, albumin-treated rats exhibited ~80% reductions in mean cortical infarct volume. Recently, we have shown that albumin treatment also improves neurobehavioral outcome in a rat model of acute intracerebral hematoma [19]. Albumin acts via multiple mechanisms, which include the amelioration of brain swelling [12,13,17]; the improvement of blood flow to critically perfused brain regions [50]; the reduction of postischemic thrombosis and blood-element adhesion within the brain's microvasculature [14]; and the mobilization and supply of important free fatty acids to the postischemic brain [81].

### 2.1.4 The Multiple Actions of the Human Albumin Molecule

The reasons for albumin's high neuroprotective efficacy, in our view, are explicable by the multiple attributes of this unique protein. Human serum albumin is the most abundant circulating plasma protein and has a prolonged persistence in the body. With a degradative rate of 3.7% per day, the life of the average endogenous albumin molecule is 27 days, with ~15,000 passages through the circulation [76]. Albumin subserves multiple crucial roles in normal homeostasis: 1) the maintenance of plasma colloid osmotic pressure; 2) the transport of fatty acids [26,107]); and 3) the transfer of cholesterol between lipoproteins and cells [118]. In addition, albumin binds many metabolites and is responsible for the majority of drug binding in the plasma [55, 56]. Albumin possesses many other important actions as well:

**Antioxidant effects:** Albumin constitutes a major antioxidant defense against oxidizing agents generated both by endogenous processes (such as neutrophil myeloperoxidase) and by exogenous mediators (e.g., phenolic dietary compounds) [43,44,49,96,110]. Indeed, plasma proteins, chiefly albumin, appear to account for up to three-fourths of the total radical-trapping antioxidant activity of plasma – fully 10-20 times greater than the effect attributable to vitamin E alone [111]. At least three mechanisms account for albumin's potent antioxidant action: 1) its reactive cysteine-34 thiol moiety; 2) its ability to bind redox-active transition metals, in particular copper ions, thereby inhibiting copper ion-dependent lipid peroxidation and formation of the highly reactive hydroxyl radical species [29,43]; and 3) its ability to bind amphipathic species such as fatty acids and heme, which may participate in injurious redox reactions [60]. As albumin is present in relatively high concentrations in both plasma and interstitial fluid, it is strategically situated to scavenge oxygen radicals, to bind to free fatty acids and metal ions, and to interrupt the damaging oxidative process of lipid peroxidation [29]. Recently, the N-terminal tetrapeptide of albumin, DAHK (Asp-Ala-His-Lys), has been shown to constitute a tight binding site for $Cu^{2+}$ ions [10]; both human albumin and its N-terminal tetrapeptide block oxidant-induced neuronal death in cortical cell cultures [41].

**Endothelial actions**: Albumin also exerts direct effects on vascular endothelium. By binding to the endothelial glycocalyx, albumin maintains the normal permeability of microvessel walls and, by its transcytosis across endothelium, it serves as a carrier for various small molecules [45, 88]. Microvascular endothelial cells express several specific albumin binding sites on their surface [34, 87, 88]. The binding of albumin probes to the endothelial cell surface appears to mediate their transcytosis or endocytosis [89]. Certain ligands such as fatty acids may increase albumin-binding to these receptors and, hence, facilitate its passage across endothelial membranes [33]. Albumin exerts complex influences on erythrocyte aggregation, increasing low-shear viscosity but decreasing erythrocyte sedimentation under no-flow conditions [79]. Recent work suggests that albumin may be a factor mediating the effect of blood coagulation on vascular tone and capillary permeability. Serum albumin reacts with nitric oxide to form a stable S-nitrosothiol that has endothelium-derived relaxing factor-like properties [54]. Albumin has also been shown to be a specific inhibitor of endothelial-cell apoptosis [119]. Evidence from studies in many organ systems supports albumin's intravascular actions: Thus, albumin markedly attenuates shock/resuscitation lung injury by reducing transpulmonary protein flux and diminishing bronchoalveolar neutrophil extravasation [78]. Albumin also inhibits the binding of activated neutrophils to bovine aortic endothelial cells in response to inflammatory stimuli [60].

**Metabolic effects:** Albumin exerts major effects on astrocytes. When applied to cortical astrocytes in culture, albumin elicits intercellular calcium waves that can be inhibited by gap-junction

blockers [70]. Albumin is also an effective mitogen for astrocytes [69], suggesting that it may be responsible for stimulating glial scar formation in pathological states in which it is able to cross a permeable blood-brain barrier into the brain. Lactate originating in glial cells appears to be an important energy substrate for recovery of synaptic function after hypoxia/ischemia [91-93] as well as during neuronal activation [90]. Albumin is a major regulator of the enzyme pyruvate dehydrogenase in astrocytes, capable of more than doubling the flux of glucose and lactate [103]. This takes on relevance in that pyruvate dehydrogenase is inhibited by ischemia, and this inhibition promotes substrate limitation, decreasing electron flow into the mitochondrial electron-transport chain [18]. As the entry of serum albumin into the brain is enhanced under pathological conditions (see Progress Report/Preliminary Studies), it is possible that this action of albumin could be responsible for sustaining neuronal metabolism under pathological conditions by increasing the export of pyruvate to neurons for metabolism via the Krebs cycle [108].

**Hemodilution – experimental studies:** While exogenous albumin produces hemodilution, we consider it very unlikely that albumin-neuroprotection is mediated by hemodilution alone: The traditional view that albumin might act solely via its oncotic, hemodiluting action, in our opinion, ignores the large body of evidence, reviewed above, that albumin is in fact a unique complex molecule with multiple salutary physiochemical properties. Our own preclinical studies support the view that these multifunctional aspects of the albumin molecule are probably integral to its striking neuroprotective effect. Hemodilution may, however, also contribute in some degree to albumin-neuroprotection. Hemodilution has been used in at least 15 experimental studies of focal cerebral ischemia in which histopathological neuroprotection was assessed [8,22-25,58,61,64,67,73,75,102,109,115,116]. Interpretation of these diverse results is limited by the variety of hemodiluents used, widely differing methods, and, often, a lack of modern-day rigor. Despite these shortcomings, these studies tend to support a beneficial effect of hemodilution, particularly in temporary rather than permanent vascular occlusion models, and with colloid agents administered in high concentrations close to the onset of the ischemic event (e.g., [23,58,75,109,116]. While human serum albumin was employed as a hemodiluent in eight of these studies [8],[22,23,25,58,64,67,102] high-concentration albumin (20-25%) was assessed in only a single, non-rigorous report [64].

**Hemodilution – clinical studies:** Prior to our own studies, the neuroprotective efficacy of albumin therapy for ischemic stroke had been studied in only one small controlled clinical trial, in which a suggestion of efficacy was present for a subgroup [38]. Several other controlled clinical trials have assessed non-albumin forms of hemodilution for acute ischemic stroke [3,9,40,46,51,84,85,99], but these trials are of limited relevance in that artificial hemodiluents lack the multiple beneficial mechanisms conferred by the albumin molecule (reviewed above).

**Conventional neurological uses of albumin - prevention of vasospasm:** Large quantities of albumin are commonly administered over prolonged time periods in patients with aneurysmal subarachnoid hemorrhage (SAH) following surgical clipping of the aneurysm, in order to prevent the delayed ischemia secondary to vasospasm [62,63,68,100].. For example, at the Massachusetts General Hospital, neurological intensivists commonly administer 5% albumin, 250 ml every 4 h, for 1 or 2 weeks if early vasospasm is detected by transcranial Doppler (Walter Koroshetz, M.D., personal communication); this dose amounts to > 1 g albumin per kg body weight per day. Incipient congestive failure, should it emerge, is routinely managed successfully. These doses resemble those that were used and well-tolerated in patients with cirrhosis and spontaneous bacterial peritonitis [98].

**Conventional neurological uses of albumin - treatment of brain edema:** Albumin has also been used in moderate-to-high doses to combat brain edema. In subjects with brain contusion, 25% albumin was administered so as to maintain elevated oncotic pressure for 2 weeks; albumin therapy

safely and effectively reduced contusional edema [105]. In another study, patients with putaminal hemorrhage treated with 12.5-25 g per day of albumin for 2 weeks showed reductions of cerebral edema and improved outcome [106]. Thus, in marked contrast to crystalloid-hemodilution, which worsens brain edema and infarction [47, 58], studies of high-dose albumin therapy have consistently suggested beneficial effects in reducing cerebral edema.

## 2.2 Supporting Clinical Data: Phase I Dose-Escalation and Safety Trial of Albumin In Subjects with Acute Ischemic Stroke (NS 40406)

### 2.2.1 Overview

This Phase I clinical trial, initiated in August, 2001, was an open-label dose-finding study of intravenous 25% human serum albumin (ALB) in subjects with acute ischemic cerebral infarction. Its objectives were the following:

- First, *to establish the safety* of administering moderate-dose ALB intravenously (in a multiple-tier, dose-escalation design) to subjects with acute ischemic cerebral infarction; and
- Secondly, to gain experience in implementing standardized measures for assessing neurological deficit, cardiovascular status, and neurological outcome as a prelude to future Phase II-III trials of this promising agent.

The trial was structured as a two-center, open-label, non-randomized dose-finding trial to evaluate the safety, and to work out the logistical details, of administering ALB intravenously to subjects with acute ischemic stroke of 16 hours' duration or less; and to implement standardized procedures for monitoring cardiovascular function and for assessing neurological outcome in these subjects. As this trial was envisioned as a prelude to multicenter randomized Phase II and III trials of this therapy, subjects were followed for three months after hospital discharge for the assessment of their NIH Stroke Scale, Barthel Index and Rankin Scale. Two clinical study sites participated: (1) The University of Miami Jackson Memorial Hospital, and 2) the University of Calgary Foothills Medical Centre. A unique advantage of the Calgary site is its access to many hyperacute stroke subjects admitted 0-3 hours after stroke onset. Data management and statistical analyses was coordinated centrally by the Data Coordination Unit at the Medical University of South Carolina.

The primary intent of the study was to evaluate the safety of escalating intravenous ALB doses and infusion rates and to determine the maximum tolerated dose (MTD) of ALB in subjects with acute ischemic stroke. The MTD was considered to be the highest dose level at which, in the view of the study's internal-plus-external Safety Evaluation Committee (SEC), ALB therapy is tolerated without the occurrence of unacceptable cardiac or other serious adverse events. The dose tiers evaluated were: (1) 0.34 g ALB per kg body weight; (2) 0.68 g/kg; (3) 1.03 g/kg; (4) 1.37 g/kg; (5) 1.71 g/kg; and (6) 2.05 g/kg. These doses were chosen based upon our extensive preclinical data showing that ALB doses of 1.25, 2.00, and 2.50 g/kg were highly neuroprotective in a rat model focal cerebral ischemia produced by middle cerebral artery occlusion. [12, 13, 17]. Two subject subgroups were represented in the trial:

1) Subjects admitted within the 0-3 hour window after stroke onset who received IV thrombolysis as well as ALB.
2) Subjects admitted within 16 hours of stroke onset who received ALB but not thrombolysis (either because they entered outside of the 0-3 hour thrombolysis window, or because they failed to satisfy one of the other thrombolysis exclusion criteria.)

In the initial dose-escalation design, separate cohorts of ~6 thrombolysis and ~6 non- thrombolysis subjects were accrued at each dose-level, and the SEC was asked to adjudicate the thrombolysis and non- thrombolysis cohorts of each dose-tier separately and to render separate decisions. As the trial progressed, however, it became evident that the thrombolysis and non- thrombolysis cohorts did not differ in the proportion of adverse events encountered. After Tier IV, the design was altered eliminating the stratification of enrollment by thrombolysis use.

### 2.2.2 Results (See publications [128,129])

**Enrollment:** A total of 82 subjects were enrolled into 6 ALB dose-tiers. Enrollment of the last subject into the final dose-tier, Tier VI, occurred on May 1, 2005.

**Demographics:** The following demographics describe the 82 subjects enrolled in Tiers I through VI:

| | |
|---|---|
| Mean age (± SD) | 65.2 ± 14.9 years; range: 25-88 years |
| Gender | Male, 43 (52%); Female, 39 (48%) |
| tPA use | tPA, 42 (51%); Non-tPA, 40 (49%) |
| Ethnic origin | Non-Hispanic Caucasian, 60 (73%) |
| | Hispanic, 9 (11%) |
| | African-American, 9 (11%) |
| | Native American, 1 (1%) |
| | Asian, 1 (1%) |
| | Other, 2 (2%) |

**Safety Considerations -- Non-Serious Adverse Events:** The only adverse events bearing a relationship to ALB therapy (comprising "unlikely", "possible", "probable", or "definite") were those related to intravascular volume expansion: i.e., shortness of breath and frank pulmonary edema / congestive heart failure in the hours or days following ALB administration. At Dose Tiers I and II, these adverse events were only rarely observed. At Dose Tier III, the cardiologists on the SEC suspected that we were beginning to enter a dose range which produced evidence of mild pulmonary congestion, at least in elderly subjects with altered ventricular compliance or pulmonary systolic hypertension. For this reason, we proposed to the DSMB that the protocol be modified to require chest x-rays at both 24 hours and 48 hours after ALB administration, and the DSMB approved this modification. Furthermore, we proposed to measure brain natriuretic peptide (BNP) to compare levels prior to ALB therapy, and 24 hours after therapy, The BNP levels may be sensitive to incipient cardiac failure related to ALB administration. After 12 subjects were recruited at Dose Tier IV, a second set of 6 subjects was requested by the DSMB. At Dose Tier IV, out of a total of 18 subjects, a consensus of SEC members noted chest x-ray evidence of pulmonary / CHF in 4 subjects. In another 3 subjects, similar changes were noted by 1 or 2 of the 6 SEC members. Of the 18 subjects in Dose Tier IV, clinical evidence of pulmonary edema was noted by SEC members in 5 subjects. In 3 of these 18 subjects, SEC members noted the use of diuretics.

Twelve subjects were studied in Dose Tier V. Of these, the SEC noted that very mild clinical symptoms of pulmonary congestion/edema occurred in 2 subjects, one of whom also had chest x-ray changes. Prophylactic furosemide was administered in 4 of these 12 subjects.

Twelve subjects were enrolled in the final dose-tier, Tier VI (2.05 g/kg). One subject developed clinical and chest x-ray signs of mild pulmonary edema, which was easily managed with 2

doses of furosemide. None of the other 11 Tier-VI subjects received furosemide. SEC members noted that four subjects of Tier VI showed mild interstitial edema on chest x-ray without clinical findings.

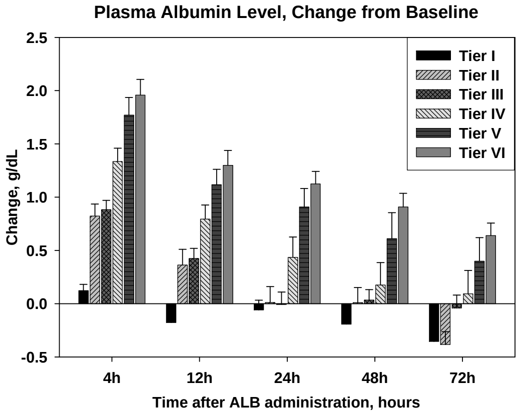


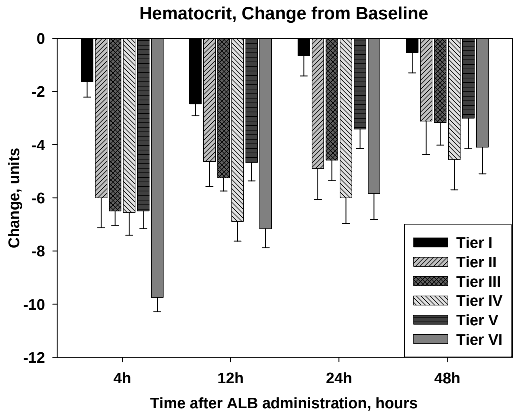


**<u>Physiological Aspects:</u>** Overall, ALB administration was well tolerated without important alterations of these variables, except for plasma albumin level itself, and hematocrit (See figures above, which present data from Tiers I-VI.)

The plasma albumin levels prior to ALB administration averaged 3.8 g/dl. ALB infusion led to a dose-related increase in plasma albumin levels, most marked at 4 hours post-infusion and declining to pre-infusion levels by 48-72 hours. In Dose Tier VI, the increase at 4 hours averaged 2.0 g/dl above baseline. Correspondingly, ALB administration produced a dose-dependent hemodilution, maximal at 4-12 hours but still present at 48h post-infusion. In Tier VI, the decrease in hematocrit at 4 hours averaged 9.8 points (23%) below the pre-infusion values.

Plasma BNP, was measured in subjects of Tiers IV, V, and VI prior to ALB infusion (n=40) and 24 hours later (n=38). Elevated BNP levels are thought to reflect increased cardiac filling pressure

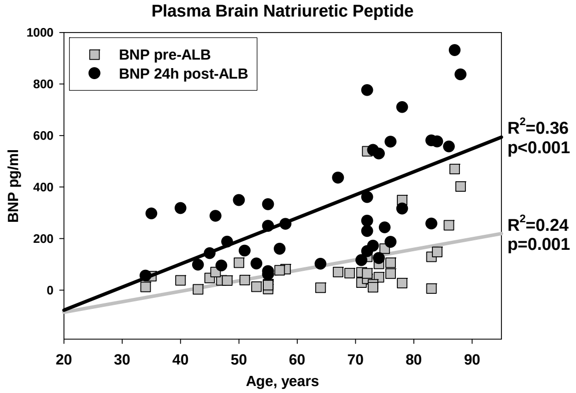


[80]. In all subjects, BNP levels rose post-ALB infusion, and subjects' age was highly correlated with BNP, both at baseline and post-ALB infusion (See figure above). However, there was no correlation between BNP (either pre- or post-ALB infusion) and initial stroke severity (NIHSS score). Interestingly, there was also *no correlation* between the extent of BNP increase and the presence or absence of cardiac adverse events. The extent of increase in plasma BNP levels post-ALB did not differ between subjects with vs. without cardiac adverse events.

ALB infusion was well tolerated at all Dose Tiers, and no evidence of ALB-induced blood-pressure increases was observed, even at the highest dose-tiers. In the **Figures ⇒**, systolic BP data are presented for the ALB dose-tiers IV - VI (units of x-axis, minutes; red line = linear regression fit).

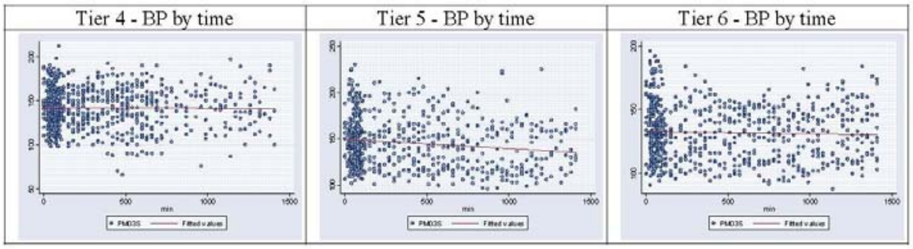


**<u>Efficacy Considerations:</u>** As this is not an efficacy trial, no conclusions as to efficacy can be drawn from this data-set. However, it is noteworthy that, at Dose Tiers IV through VI, we exceeded the per-kilogram ALB dose that in our pre-clinical studies was highly neuroprotective in focal ischemia [36].

Evaluation of our data for efficacy is complicated by the fact that, not unexpectedly, the thrombolysis cohort received ALB administration significantly *earlier* than the non-thrombolysis cohort. This is critically important because pre-clinical evidence suggested that any neuroprotective effect of ALB therapy would diminish to zero if ALB were administered more than 5 hours from stroke onset. In this pilot trial, subjects in the thrombolysis and non-thrombolysis cohorts received ALB at 6.5 ± 3.0h (mean ± SD) and 9.1 ± 3.3h after stroke onset, respectively. The latter time-range is very likely outside of the therapeutic window for ALB. This notwithstanding, we interrogated the database for suggestions of a dose-response relationship.

The figures below compare initial and final NIHSS scores in the subjects of the trial. The "final" NIHSS scores were at 3 months for subjects who had completed entire study; or at 1 month, if that point had been reached; or the last available NIHSS score for those subjects who died. For the

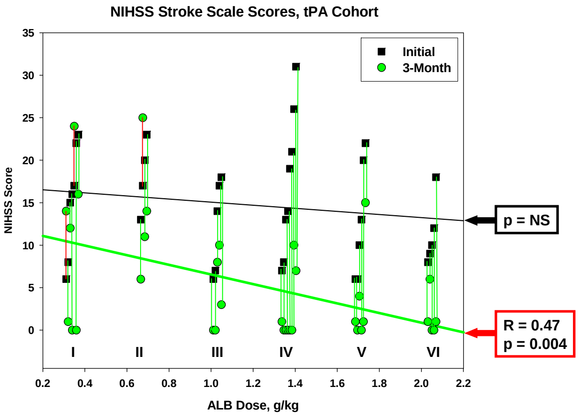


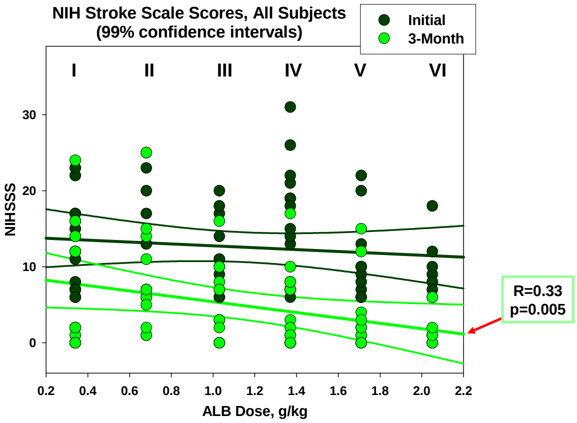


thrombolysis cohort (left panel), linear regression analysis revealed a highly significant improvement in the final NIHSS score at higher ALB dose-tiers (R=0.47, p=0.004). For the combined all-subjects dataset (right panel), there was a similar trend (R=0.33, p=0.005). The minimal suggestively effective ALB dose observed here (~1.2 g/kg and above) agrees well with the minimal ALB dose that was needed to achieve robust neuroprotection in our preclinical studies (~1.2 g/kg) [13].

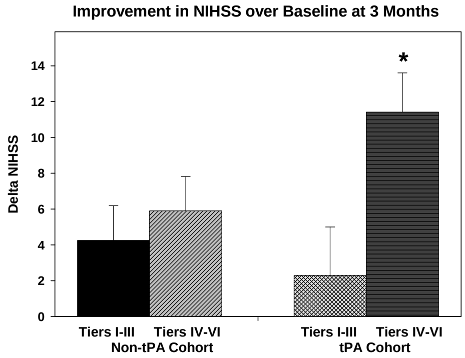


The figure to the left shows the effect of increasing ALB dose-tier on improvement of NIHSS score (final minus baseline score) for the thrombolysis and non-thrombolysis cohorts. In the thrombolysis cohort, comparison of low-dose ALB (pooled Tiers I - III) with the highest, putatively effective ALB doses (pooled Tiers IV - VI) revealed a significant effect of the higher ALB doses on long-term NIHSS score improvement over baseline compared to the lower-dose groups (p=0.015).

In this analysis, favorable outcome was defined as NIHSS score of 0-1, <u>*or*</u> modified Rankin score (mRS) of 0-1, <u>*or both*</u>, at 3 months (or at 1 month, if 3-month data were not yet available).

We chose this composite outcome because neuroprotection experimentally is defined by the volume of brain tissue salvage. In humans, this is best measured clinically on the NIHSS score. However, outcome preferences among patients indicate that a majority of stroke victims or potential stroke victims most value a complete functional recovery. As a corollary, a majority of people would prefer death than severe disability [21,83,97]. A complete functional recovery is best reflected by a mRS score of 0-1.

We compared the dose-tier IV-VI subjects of the present trial (ALB, 1.37-2.05 g/kg; N=40), with outcome data derived from the NINDS tPA Trial, Part 2 [71], using those subjects with initial NIHSSS of 6 or greater (N=146 in thrombolysis cohort, N=156 in non- thrombolysis cohort). (Subjects in the NINDS tPA Trial, of course, received no ALB.) Generalized linear model with logit link [10] analysis yielded the following relative risks (RRs):

| | | | mRS ≤ 1 | | NIHSS ≤ 1 | | Good Outcome | | | | | |
|---|---|---|---|---|---|---|---|---|---|---|---|---|
| | | **N** | **N** | **%** | **N** | **%** | **N** | **%** | **RR** | **95% CI** | **Adj. RR**** | **95% CI** |
| **No tPA** | **NINDS** | 156 | 35 | 22% | 27 | 17% | 41 | 26% | | | | |
| | **Tiers IV-VI** | 20 | 8 | 40% | 7 | 35% | 9 | 45% | 1.90 | 1.14, 3.17 | 1.20 | 0.69, 2.10 |
| **tPA** | **NINDS** | 146 | 50 | 34% | 40 | 27% | 54 | 36% | | | | |
| | **Tiers IV-VI** | 22 | 15 | 68% | 14 | 63% | 16 | 72% | 1.97 | 1.41, 2.74 | 1.67 | 1.14, 2.45 |
| **All** | **NINDS** | **302** | **85** | **28%** | **67** | **22%** | **95** | **31%** | | | | |
| | **Tiers IV-VI** | **42** | **23** | **54%** | **21** | **50%** | **25** | **59%** | **1.95*** | **1.47, 2.57** | | |

* Adjusted for tPA
** Adjusted for tPA, ALB x tPA interaction, baseline NIHSS score, and age

These comparisons against historical controls (1) suggest that ALB therapy at dose-tiers IV-VI is highly effective in improving outcome; and (2) confirm that thrombolysis is also effective.

We also compared the Dose Tiers IV-VI with Dose Tiers I-III (0.34-1.03 g/kg) in a similar manner, with the presumption that the latter group represents a control group since those early dose-tiers are assumed to be non-therapeutic levels of ALB. The results are as follows:

| | | N | mRS ≤ 1 | | NIHSS ≤ 1 | | Good Outcome | | | | | |
|---|---|---|---|---|---|---|---|---|---|---|---|---|
| | | | N | % | N | % | N | % | RR | 95% CI | Adj. RR** | 95% CI |
| **No tPA** | **Tiers I-III** | 20 | 7 | 35% | 6 | 30% | 9 | 45% | | | | |
| | **Tiers IV-VI** | 20 | 8 | 40% | 7 | 35% | 10 | 45% | 1.11 | 0.58, 2.14 | 1.31 | 0.72, 2.39 |
| **tPA** | **Tiers I-III** | 20 | 5 | 26% | 5 | 25% | 5 | 25% | | | | |
| | **Tiers IV-VI** | 22 | 15 | 68% | 15 | 68% | 16 | 72% | 2.91 | 1.31, 6.49 | 2.75 | 1.32, 5.73 |
| **All** | **Tiers I-III** | **40** | **12** | **30%** | **11** | **27%** | **14** | **35%** | | | | |
| | **Tiers IV-VI** | **42** | **24** | **57%** | **23** | **54%** | **26** | **61%** | **1.81*** | **1.11, 2.94** | | |

* Adjusted for tPA
** Adjusted for tPA, ALB x tPA interaction, baseline NIHSS score, age, and time from symptom onset to ALB treatment

Although the ALB effect remains highly significant in the thrombolysis cohort, caution is advised in its interpretation due to the wide confidence intervals on the RRs arising from the much smaller sample sizes for this analysis than in the previous comparison. In these analyses, the RRs and the absolute risk differences between the thrombolysis and non-thrombolysis cohorts are suggestive of an interaction effect between ALB and thrombolysis.

## 2.3 Summary of Background and Rationale

To summarize, the multifunctional nature of the albumin molecule renders it uniquely suited as a neuroprotective agent for acute cerebral ischemia. Our preliminary studies support this assertion and provide a strong rationale for the proposed clinical trial in human ischemic stroke. The rationale for a Phase III randomized controlled trial of human albumin (ALB) is the following:

- Apart from thrombolytic therapy, no pharmacological neuroprotective strategies are currently available for the reduction of brain damage after acute ischemic stroke.
- Extensive preclinical investigations in experimental models of focal and global cerebral ischemia and traumatic brain injury have convincingly shown that the prompt intravenous administration of moderate-to-high doses of ALB confers consistent and substantial neuroprotection. These studies have shown that the therapeutic window for robust neuroprotection in focal ischemia extends to at least 4 hours after stroke onset, and that a partial therapeutic effect is evident when ALB is administered at 5 hours [13].
- Our NIH-funded Phase I Dose-Escalation and Safety Trial of Albumin (hereafter referred to as Albumin Phase I Trial) has completed recruitment of all 6 pre-specified ALB dose-tiers and has convincingly demonstrated the feasibility of administering high-dose ALB without the development of dose-limiting cardiovascular or neurological adverse events. Significantly, the

higher dose-tiers fall well within the per-kg dose-range of 1.25-2.50 g/kg shown in preclinical studies to confer high-grade neuroprotection [13]. The ALIAS Trial affords the unique opportunity to apply a preclinically highly efficacious neuroprotective strategy to the treatment of acute ischemic stroke using both a dose and timing that very closely replicate the experimental setting in which efficacy was demonstrated.

- Clinical studies of moderate-to-high dose ALB therapy in cerebral contusion and hemorrhage have shown a consistent efficacy in reducing cerebral edema, and pre-clinical work in our lab suggests that ALB is also neuroprotective in intracerebral hematoma [15]. Moderate-to-high-dose ALB is routinely used in subjects to combat vasospasm following aneurysmal clipping for subarachnoid hemorrhage. These doses are well tolerated, and incipient congestive heart failure, if present, is readily managed.
- The rationale of the ALIAS Trial is further supported by the fact that ALB is a unique protein molecule that acts via a multifunctional spectrum of properties that, in our view, are integral to its neuroprotective action. These include its prolonged persistence in the circulation; its capacity to bind fatty acids, metal ions, metabolites, and exogenous drugs and agents; its marked ability to antagonize oxygen radical formation and lipid peroxidation; its salutary effects on vascular endothelium; and its beneficial metabolic influence on astrocytic function.
- ALB is an inexpensive therapy, costing approximately $900 per 600 ml dose (150 g ALB) --the expected dose for a subject weighing 87.5 kg. It is given as a single infusion and has had uncommon and simple side effects in the Albumin Phase I Trial. (By comparison, a single vial of thrombolysis costs over $2,000.) Because we are searching for a similar magnitude of effect of thrombolysis (10% effect size) and because the safety profile of ALB in general and in acute stroke is so favorable, we assume that ALB is very likely to be a highly cost-effective drug.

## 3. STUDY DESIGN

The ALIAS Trial consists of a multicenter, randomized, double-blind, parallel two-arm Phase III trial to assess whether IV ALB therapy confers neuroprotection in acute ischemic stroke over and above the standard of care among patients with acute ischemic stroke. Eligible subjects are randomized 1:1 to either ALB or saline and the primary outcome is assessed at the 3-month clinic visit. Subjects are followed for 12 months from randomization via telephone contact at 1, 6, 9 and 12 months to determine the durability of the effect of ALB therapy. Subjects may receive thrombolytic therapy and are expected to receive such therapy in a timely fashion according to the standard of care. Randomization is stratified according to whether or not the subject receives thrombolytic therapy.

## 4. SELECTION AND ENROLLMENT OF SUBJECTS

### 4.1 Inclusion Criteria

1. Acute ischemic stroke.
2. Age 18 years through 83 years (i.e., have not had their 84$^{th}$ birthday).

3. NIH Stroke Scale (NIHSS) score of 6 or greater as assessed immediately prior to thrombolysis treatment if patient is eligible for thrombolysis or immediately prior to randomization for patients not eligible for thrombolysis.

Initiation of ALB/placebo can begin within 5 hours of stroke onset, and within 60 minutes of the start of the thrombolysis infusion, if IV thrombolysis is given. [An exception is permitted in the case of subjects judged to be fully eligible for the ALIAS Trial pending confirmation of normal serum troponin level; in such a situation, an interval of up to 90 minutes between start of thrombolysis or randomization is permitted to allow for receipt of the serum troponin lab report.]
(The time of stroke onset is defined as the time at which the subject or observer first noted the onset of neurological abnormality. In the event that stroke symptoms were first noticed on awakening or were not witnessed, the time of onset is the last time the subject was observed to be normal or without stroke symptoms.)

4. Signed and dated informed consent has been obtained.

## 4.2 Exclusion Criteria

1. Episode/exacerbation of congestive heart failure (CHF) from any cause in the last 6 months. (An episode of congestive heart failure is any heart failure that required a change in medication, diet or hospitalization.)
2. Known valvular heart disease with CHF in the last 6 months.
3. Known (or in the Investigator's judgment) existence of severe aortic stenosis or mitral stenosis
4. Cardiac surgery involving thoracotomy (e.g., coronary artery bypass graft (CABG), valve replacement surgery) in the last 6 months.
5. Acute myocardial infarction in the last 6 months.
6. Signs or symptoms of acute myocardial infarction, including EKG findings, on admission.
7. Elevated serum troponin level on admission > 0.1 mcg/L.
8. Suspicion of aortic dissection on admission.
9. Acute arrhythmia (including any tachy- or bradycardia) *with* hemodynamic instability on admission (systolic blood pressure < 100 mmHg).
10. Findings on physical examination of *any* of the following: (1) jugular venous distention (JVP > 4 cm above the sternal angle); (2) 3rd heart sound; (3) resting tachycardia (heart rate > 100/min) attributable to congestive heart failure; (4) abnormal hepatojugular reflux; (5) lower extremity pitting edema attributable to congestive heart failure; (6) bilateral rales; and/or (7) if a chest x-ray is performed, definite evidence of pulmonary edema, bilateral pleural effusion, or pulmonary vascular redistribution.
11. Current acute or chronic lung disease requiring supplemental chronic or intermittent oxygen therapy.
12. Historical modified Rankin Scale (mRS) $\geq$2. Patients who live in a nursing home or who are not fully independent for activities of daily living (toileting, dressing, eating, cooking and preparing meals, etc.) immediately prior to the stroke are not eligible for the trial.
13. Patients with stroke occurring as a complication of hospitalization for another condition, or as a complication of a procedure.
14. Profound dehydration
15. Fever, defined as core body temperature > 37.5°C (99.5°F).
16. Serum creatinine > 2.0 mg/dL or 180 µmol/L.
17. Severe chronic anemia (hemoglobin < 7.5 g/dL or 75g/L).

18. Evidence of intracranial hemorrhage (intracerebral hematoma, intraventricular hemorrhage, subarachnoid hemorrhage (SAH), epidural hemorrhage, acute *or* chronic subdural hematoma (SDH)) on the baseline CT or MRI scan.
19. History of or known allergy to albumin.
20. History of or known allergy to natural rubber latex.
21. Pregnancy, breastfeeding or positive pregnancy test. (Women of childbearing age must have a negative pregnancy test prior to study drug administration.)
22. Concurrent participation in any other therapeutic clinical trial.
23. Evidence of any other major life-threatening or serious medical condition that would prevent completion of the study protocol, impair the assessment of outcome, or in which ALB therapy would be contraindicated or might cause harm to the subject.

For patients receiving IV thrombolysis, investigators also should follow appropriate IV thrombolysis exclusion criteria according to current guidelines (e.g., American Stroke Association, Canadian Stroke Strategy) in their clinical assessment of potential patients for the trial. A detailed list of the criteria is included in the MoP.

## 4.3 Study Enrollment Procedures

### 4.3.1 Screening of Potential Subjects

The Study Schema below outlines the screening and enrollment process. Study screening, informed consent and randomization procedures should not precede standard-of-care procedures for the patient. The administration of thrombolysis, if determined to be appropriate for the patient, should proceed without delay.

It is expected that greater than 95% of the recruitment will occur in the ED. Neurology residents at each clinical site should be made aware of the ALIAS Trial. It is the responsibility of the local study site Principal Investigator (PI) to ensure that ALL staff who could potentially be involved with the treatment of an ALIAS subject, including triage and emergency room nursing staff and physicians, receive in-service training about the study. Local paramedic and EMT services also should be informed about the study.

All potential stroke subjects are identified by a triage physician or nurse in the ED, who examines the subjects within minutes of their arrival. If an acute stroke is suspected, a neurology resident or fellow (or the attending staff neurologist) is immediately called to the ED and evaluates the subject within the next few minutes. A Stroke Fellow (PGY 5) or staff physician, available within approximately 10 minutes, immediately alerts the Stroke Service attending physician. Potentially eligible subjects are then reviewed by the Stroke Team and the Study Coordinator.

All sites are expected to be capable of mounting an acute stroke response, which includes the potential for thrombolytic therapy.

### 4.3.2 Screening/Baseline Evaluations

**Clinical Examination:** All potential subjects undergo a stroke neurological history and examination and a brief general history and examination focusing on the cardiorespiratory system. Prior medication use is documented. The time of stroke onset is determined by the stroke response

team. A standardized history ensures that the subject fulfills inclusion and exclusion criteria and establishes a baseline stroke risk factor profile. Subjects undergo a standardized cardiac examination. This includes, but is not limited to, assessment of blood pressure, pulse, bedside estimation of the jugular venous pulse (JVP), evaluation of the hepatojugular reflux, auscultation of the heart for the presence of a third heart sound (S3), evaluation of a standard 12-lead EKG tracing, and evaluation of a chest X-ray, if one is done. The NIHSS measured immediately before administration of IV or IA thrombolysis (whichever comes first) for those subjects being thrombolysed, and immediately before randomization for the non-thrombolysis subjects, is considered the baseline NIHSS score. This is the assessment that is entered into WebDCU™ for the Baseline visit.

As a part of the initial clinical examination, all patients are assessed for potential eligibility for participation in the study. A Screening Log is maintained at each site. The Screening Log includes all patients who meet the following three requirements whether or not they ultimately participate in the study. The three criteria are: 1) 18-83 years of age; 2) clinical evidence of ischemic stroke; and, 3) were actively screened by the clinical site's ALIAS stroke team. Maintaining a Screening Log at each site helps to reduce any perception of selection bias.

**Imaging:** Subjects undergo a baseline neuroimaging procedure (CT or MRI) to rule out intracranial hemorrhage. The baseline CT scan should be performed using a standard stroke algorithm. A MRA and PWI are not a prerequisite of the ALIAS Trial. Save for the presence of hemorrhage, there are no exclusion criteria for the ALIAS Trial based upon the baseline CT or MRI appearance. The study is based upon the clinical diagnosis of acute ischemic stroke. Subjects who are treated with thrombolytic therapies should be treated according the local standard of care, which may require attention to the degree of ischemia shown on the baseline imaging. Baseline CT/MRI scans are evaluated both locally by the investigator at the time of assessment, and centrally at the University of Calgary at a later time.

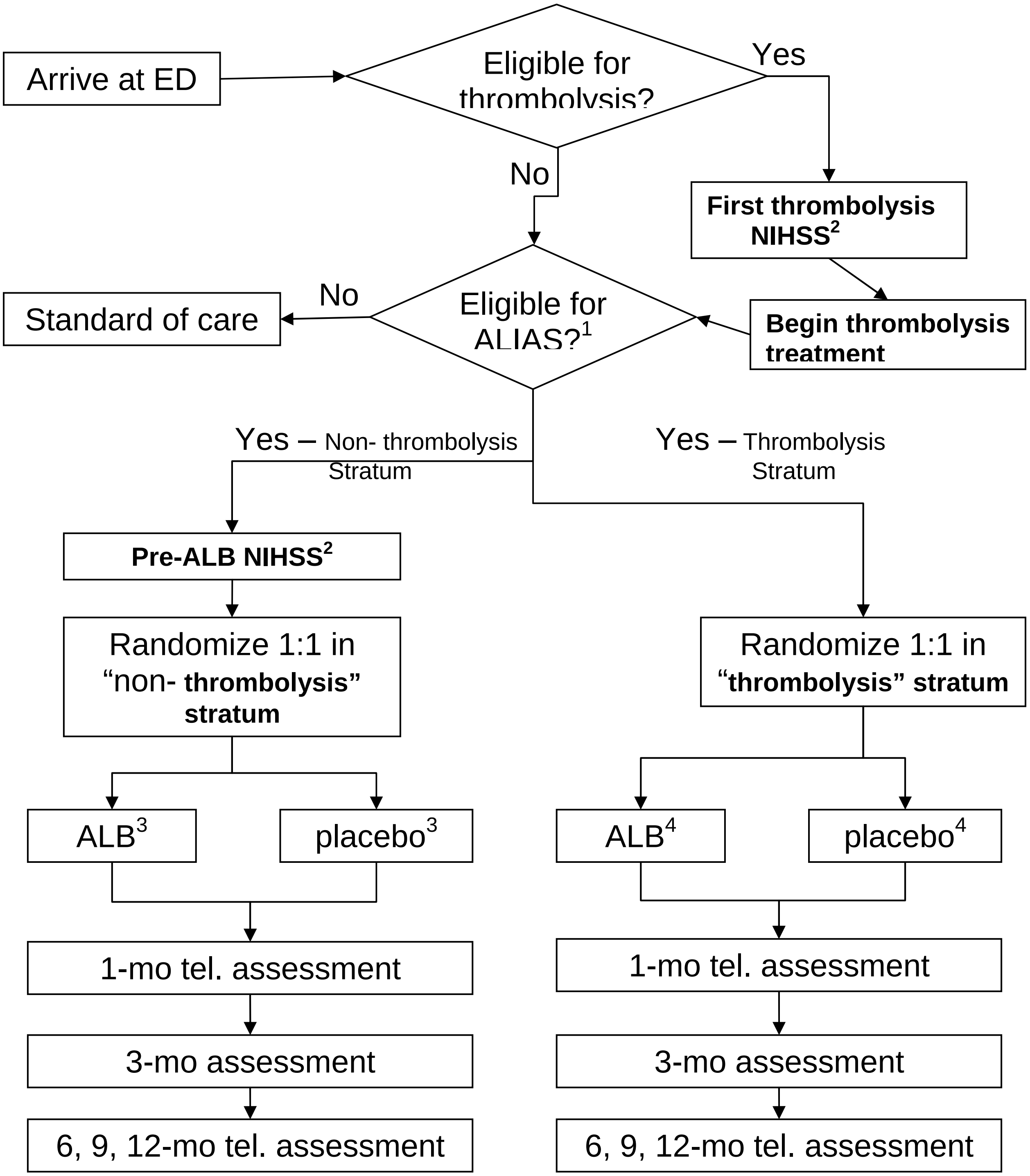


[1] Including informed consent

[2] Baseline NIHSS for the respective strata
[3] Study tx must begin within 60 minutes of randomization
[4] Study tx must begin within 60 minutes of IV thrombolysis tx initiation

**Laboratory and Other Procedures:** All subjects must have an electrocardiogram and routine blood work including white blood cell count (WBC), serum creatinine, International Normalized Ratio (INR) and baseline serum troponin. A chest X-ray is not required; however, if one is done and it shows evidence of pulmonary edema, bilateral pleural effusion, or pulmonary vascular redistribution, the patient must be excluded. This process is the same as a routine hyperacute assessment of subjects for thrombolysis therapy. The plasma creatinine level must be reviewed *prior to* randomizing the subject as it is needed to determine eligibility (see Section 4.2). The following pre-treatment tests should be ordered.

- Hemoglobin, hematocrit, white blood cell count, and platelet count
- Creatinine, potassium, sodium, chloride, bicarbonate, and glucose
- Activated partial thromboplastin time; prothrombin time (INR)
- Troponin levels. (Levels must not be elevated)
- 12-lead electrocardiogram
- Brain CT or MRI scan
- Pregnancy test for women of childbearing age.

**Informed Consent:** Upon confirmation of a patient's eligibility based on standard clinical management for patients presenting with symptoms of acute stroke, consent is obtained by either the clinical site PI or by individuals approved by him/her and whose names and copies of curricula vitae have been submitted to the DCU. In accordance with US FDA regulations (21 CFR 50) and ICH-GCP Consolidated Guidelines (Federal Register, May 9, 1997, Vol. 62, Number 90), it is the investigator's responsibility to ensure that witnessed informed consent is obtained from the patient or the patient's legally authorized representative before the patient may be enrolled in an investigational study. Before signing the consent form, the patient or the patient's legally authorized representative must have been given an adequate explanation of the purpose, methods, risks, potential benefits and patient responsibilities of the study. The consent form must be an up-to-date document that has been approved by the clinical site's IRB/REB. A signed and dated informed consent is required prior to randomization. A sample informed consent form is provided in the MoP.

Obtaining informed consent for participation in the ALIAS Trial must not delay IV thrombolysis treatment and must never interfere with a patient receiving the standard of care for that clinical site. In the case of a patient who is eligible to receive IV thrombolysis, the patient should receive IV thrombolysis if that is the standard of care at that site. If that patient is eligible for the trial, the site principal investigator, or his/her designee, must obtain informed consent after initiating IV thrombolysis and before proceeding with enrollment in the trial.

### 4.3.3 Treatment Assignment

Due to the emergency nature of randomization in acute stroke clinical trials, treatment assignments must take place in an expeditious manner that also ensures even distribution in the treatment groups. For the ALIAS Trial, the centralized randomization process, via the study website, uses a stratified (by thrombolysis and clinical site) biased coin approach. The following "Step Forward" web-based randomization is enabled because ALIAS is a blinded study. Subjects are randomized in a 1:1 ratio to treatment with albumin (ALB) or placebo solution.

Prior to enrollment at each site, the appropriate treatment kits for each of the 2 strata are designated with a "Next" flag. When a subject is enrolled into the study, the site staff goes to the stock of study drug kits and takes the drug kit with the "Next" flag on it, for the appropriate stratum. Within 8

hours of subject randomization, the Study Coordinator, or designee, must enter the randomization information (including the treatment kit identification number) into the ALIAS Trial website. Upon submission of that information, the computer immediately assesses the treatment imbalance within and across the clinical sites, and generates and informs the Study Coordinator/Study Drug Recipient online which treatment kit to use for the next subject that becomes eligible. The Study Coordinator/Study Drug Recipient then "flags" that kit as the one to use for the next eligible subject in that stratum. Because this is a blinded study, the clinical site staff will not know whether the next kit is ALB or placebo.

When the next eligible subject at the clinical site is identified, the Study Coordinator, or designee, selects the kit for that stratum which is flagged based on the designation made earlier by the computer. Hence, after each subject is randomized, the treatment assignment is made for the subsequent subject (within that stratum) once the current subject's randomization data are entered into the ALIAS randomization website.

The Study Coordinator, or other personnel, retrieves the flagged study drug kit (500 ml and 250 ml vials containing either ALB or saline-placebo as well as the tubing for the IV administration). The study kit should not be opened until everything is in place to administer the drug (including signed informed consent, IV lines, a final decision on thrombolysis, receipt of serum troponin result, etc.). Once the study kit is opened, the subject is randomized into the trial and must be followed through the entire 12-month follow-up period, unless the subject dies or withdraws from the study. A floor nurse or other personnel not involved with the trial proceeds with the study drug administration in the ED, the ICU, or in the Stroke Unit, as appropriate to the respective clinical sites, and depending upon the subject's clinical status.

The label with the hidden treatment identification (ALB or placebo) is attached to the 500 ml vial of every study treatment kit. Upon opening the kit, the unadulterated label must be affixed to the subject's Randomization CRF, which is to be kept in his/her study folder at the clinical site.

A subject is considered to be in the ALIAS Trial when he or she is randomized, and the time of randomization is when the subject's treatment kit is unsealed. This is why it is important not to open a kit prematurely.

### 4.3.4 Emergency and Accidental Unblinding

Unblinding should almost never be necessary. However, it may be required on rare occasions for unanticipated safety reasons. Before unblinding of a particular subject, the AMMs (Drs. Ginsberg and Hill) should be consulted if possible. However, in case of an emergency need for unblinding of a particular subject, the clinical site PI or his/her designee can scratch the cover material off the randomization label that should be in the subject's study folder. Site monitors ensure that these labels are properly stored (with the respective subject's study folder) and remain untouched unless a life-threatening situation occurs such that unblinding becomes necessary.

Should either an accidental or deliberate unblinding event occur, the clinical site individual who was unblinded personally must call the ALIAS Trial emergency phone number to report the event to an AMM, who will maintain a log of these unblinding events. The incident should not be discussed with other clinical site personnel.

# 5. STUDY INTERVENTIONS

## 5.1 Interventions, Administration, and Duration

The weight of the subject is obtained by self-report from the subject, from the person accompanying him/her to the ED, or is estimated by the treating physician. Subjects are not weighed in the acute phase in order to prevent delay in therapy, but they should be weighed sometime between 24 to 48 hours from randomization. Both the estimated and actual weights are recorded. Subjects receive weight-adjusted ALB or placebo infusion at a constant rate over 2 hours (+/- 15min.).

All subjects must start their ALB/placebo infusion within 60 minutes of the start of thrombolysis (usually the IV thrombolysis infusion) for thrombolysis subjects and within 60 minutes of randomization (unsealing of the study drug kit) for the non-thrombolysis subjects. [An exception is permitted in the case of subjects judged to be fully eligible for the ALIAS Trial pending confirmation of normal serum troponin level; in such a situation, an interval of up to 90 minutes between start of thrombolysis or randomization is permitted to allow for receipt of the serum troponin lab report.] The ALB/placebo infusion must not delay standard of care thrombolytic therapy. Planned acute use of IA thrombolysis or acute endovascular intervention (e.g., stenting, angioplasty, thrombus retrieval device use/mechanical thrombectomy) is permitted if treatment will: (1) begin within 5 hours of symptom onset; and, (2) be completed within 7 hours of symptom onset. ALB/placebo must be administered through a dedicated IV line.

The placebo solution consists of isotonic saline solution. As 25% human albumin is a yellowish-brown liquid and the saline-placebo solution is clear, masking cartons and opaque tubing or other masking materials (such as opaque sheaths) are used to maintain blinding. ALB and saline-placebo are packaged into identical alphanumerically encoded IV delivery systems consisting of masked bottles and IV administration sets chosen to obscure their identity. To further maintain blinding, a staff nurse not involved in the ALIAS Trial will hang the IV study drug/placebo and eliminate dead-space fluid from the IV line prior to starting the infusion. Similarly, this individual, or another staff nurse not involved in the ALIAS Trial, also will remove the study drug/placebo materials after the infusion and discard them after dose verification. Two individuals not associated with the ALIAS Trial must complete the Dose Verification Form. One of the individuals enters the dose to be given and signs and dates the form; the second individual enters the amount of material remaining in the vial(s) after the infusion. That person also signs and dates his/her entry, which is on the same form as the dose entry. The independent clinical research associate (monitor) will review the Dose Verification Form to check the subject identification number and the appropriate administration of the study drug/placebo dose.

Bottles of ALB/placebo are 500ml and 250ml in size, and each subject has one bottle of each size assigned to him/her. Some subjects require only the 500ml bottle, but for heavier subjects, the second bottle may be needed. The 500 ml bottle is ALWAYS administered first. Subjects randomized to the ALB arm of the Trial receive a dose of 2.0 g/kg body weight of 25% human albumin (Baxter); this corresponds to a volume of 8 ml/kg body weight. In any event, the maximum ALB volume to be administered is 750 ml (187.5g) per subject, irrespective of a weight greater than 206 lbs (93.75 kg). Subjects randomized to the isotonic saline placebo arm of the Trial receive 8 ml/kg of placebo solution, not to exceed 750 ml. These solutions are delivered by constant intravenous infusion over a period of 2 hours (+/- 15 mins.). Although there is a 2 hour infusion time, the priority is that the full dose of the ALB or placebo be administered to the subject.

Subjects are monitored with vital signs (heart rate, blood pressure, respiratory rate, $O_2$ saturation) and neurological vital signs (level of consciousness, any change in neurological status from baseline) at a minimum of four times daily (q6h) during the entire acute hospitalization. Plasma albumin levels should not be measured within 48 hours of treatment. Respiratory distress, tachycardia, drop in arterial oxygen saturation ($SaO_2$, by pulse oximetry) by 5% or more, or a greater than 20% spontaneous (i.e., not drug-induced or expected) change in mean arterial pressure shall trigger immediate review by the physician on-call.

## 5.2 Handling of Study Interventions

### 5.2.1 Availability and Manufacture

Baxter Healthcare Corporation (Baxter BioScience, Westlake Village, CA) provides the 25% ALB and isotonic saline-placebo solution. It prepares 25% human albumin in accordance with its customary manufacturing and quality-control procedures. The solutions are supplied in one 500 ml and one 250 ml bottle for each subject since the maximum per subject dose is 750 ml. Saline-placebo will be packaged in bottles of the same sizes. In addition, Baxter also furnishes two (2) IV administration sets for each of the study drug kits.

### 5.2.2 Packaging and Blinding

Blinding of study drugs for the ALIAS Trial requires that the containers for ALB and saline-placebo be identical and indistinguishable and that the product identity must not be revealed while flowing through the administration set. Baxter Healthcare quality-control procedures necessitate the use of clear bottles for visual inspection. Once inspected at Baxter and shipped to DHHS-SSC (see below), these bottles are enclosed in masked cartons. The IV administration sets consists of opaque tubing, similar to the material employed in the SAFE Study, or masking sheaths to use over clear tubing. These tubing sets permit IV delivery of ALB or saline-placebo by gravity-feed. Additionally, the opaque tubing sets incorporate a compressible reservoir that permits the rate of delivery to be increased, if needed, by manual compression.

### 5.2.3 Labeling, Preparation of Kits, and Distribution to Clinical Sites

The Program Support Center, Supply Service Center, Department of Health and Human Services (DHHS-SSC), Perry Point, MD, has committed to serve as the drug distribution center for the ALIAS Trial. Periodically during the ALIAS Trial period, DHHS-SSC receives the study materials from Baxter Healthcare – i.e., the 500 ml and 250 ml vials of 25% human albumin and matching saline-placebo, as well as blinded IV administration sets and blinding materials (masked cartons for the bottles).

DHHS-SSC then places the vials within the blinding cartons and seals them. DHHS-SSC adheres a double-blind label to the blinding boxes (or, masked cartons). Both the 500 ml and 250 ml boxes in a set have identical labels. Additionally, the emergency unblinding label (described earlier in section 4.3.4) is attached to the 500 ml box. The set of two boxed vials is then packaged by the DHHS-SSC into a kit along with the necessary materials for the administration of the study drug or placebo. Each kit is labeled identically to the two vials inside the kit. These study-drug kits are then

distributed according to a pre-specified schedule to the clinical sites in the United States and Canada; and, at a later time possibly other countries.

.

The treatment kit identification numbers and treatment assignments are generated at the DCU. The DHHS-SSC coordinates its activities with the DCU and enters inventory and shipping data into the WebDCU™, allowing the DCU staff to track study drug inventory status at DHHS-SSC.

### 5.2.4 Storage and Disposition

Since the ALIAS Trial Part 2 is projected to run over 5 years, and we have adopted a conservative nominal shelf-life for ALB at 2 years, multiple manufacturing runs and deliveries are required. Based on the estimated number of subjects to be recruited in each year of Part 2 of the Trial, Baxter Healthcare manufactures 300 or more study-units of 25% ALB per year, in Years 01, 02, 03, 04, and 05 if necessary, and provides corresponding units of isotonic saline solution.

Both ALB and isotonic saline solution are stable at room temperature. Thus, the Study Coordinator, or other site designated personnel, at each clinical site receives the study drug kits in small shipments (approximately four to eight kits per site depending on the activity of the site). The study drug kits should be stored in a secured cabinet at room temperature. The DCU maintains and continuously updates the database of subjects recruited at each site, and it instructs DHHS-SSC to provide additional kits to the respective site as its recruitment progresses.

Any materials remaining in the opened kit after treatment of subjects can be destroyed and/or discarded by the site in accordance with their standard clinical practices, provided that the site has entered the required information on the Dose Verification Form (see Section 5.1). Should the site be unable to have the Dose Verification Form completed, the site must retain the study drug/placebo vials until inspected by an independent clinical research associate. Those kits that have not been opened and are not useable (e.g., beyond the expiration date) or remain at the end of the Trial will be tracked by WebDCU™. Kits that expire during the course of the trial should be returned UNOPENED to the DHHS-SSC according to directions that will be sent by the DCU. At the end of the trial, the DCU will send instructions for handling these kits. In most situations, the site will be instructed to destroy the kits according to the established policies at that site.

## 5.3 Concomitant Interventions

### 5.3.1 For Both Thrombolysis and Non-Thrombolysis Subjects

According to the principle of the ALIAS Trial as a large simple trial of ALB compared to placebo over and above the best standard of care, few restrictions on concomitant care are made. However, one exception is that fluid management procedures and diuretic use mandates must be followed.

**Mandatory**

**Fluid Management:** Subjects shall receive no additional intravenous fluid during infusion of study drug with the exception of standard IV thrombolysis. IV thrombolysis should be given, if

appropriate, through a dedicated IV catheter. After completion of study drug infusion (2 hour +/- 15 min.), the subject may receive no more than 75 cc/h of IV fluid for the next 46 hours. Normal saline (0.9% NS) is the preferred IV solution since glucose may promote worsening of ischemic brain injury. The total intravenous fluid received in the first 48 hours of care should not exceed 4200ml (~600 ml study drug + 75ml/h 0.9% NS + 90 ml thrombolysis = 4140 ml). Note that obese or large subjects >100 kg may receive 4290 ml according the same formula. Strict ins (IV fluids only) and outs should be measured for the first 48 hours of a subject's care. There may be situations where additional fluid is required because of a subject's condition (e.g., shock or hemorrhage). Any exceptions made to the fluid administration mandate must be documented in the CRF.

**Diuretics:** In general, stroke patients should be treated aiming for euvolemia. One potential complication of ALB infusion is volume overload with subsequent pulmonary congestion. Diuretics may be used in the acute phase to promote diuresis to allow subjects to better compensate for an episode of pulmonary congestion. Loop diuretics such as furosemide are preferred because they act quickly and can be administered intravenously. A single dose of furosemide, 20 mg IV, (or an equivalent loop diuretic such as ethacrynic acid) should be administered as a routine standard-of-care between 12 and 24 hours after the start of study drug administration. If the treating physician chooses not follow this treatment guideline, the reason for withholding diuretic must be documented in the case report form. From the time of treatment through 24 hours, all subjects are evaluated for evidence of respiratory compromise due to congestive heart failure.

**Recommended Best Care:** Over the first 7 days of in-patient care, subjects' cardio-respiratory status should be reviewed with particular care. It is over this time period that ALB will redistribute in the body and exert an oncotic effect. Acute stroke subjects in the ALIAS Trial require a level of scrutiny that neurologists may not be familiar with. Close continued attention to the fluid balance, and cardiac status is required. Treating neurologists should have a low threshold to promote diuresis during the acute hospital stay.

**Antiplatelet/anticoagulant therapy:** Antiplatelet therapy should be administered to all subjects within 48 hours of their stroke. Aspirin (ASA, acetylsalicylic acid), 81-325 mg daily (low dose) is the preferred drug. Subjects who are naïve to ASA may be loaded with 160 mg ASA on the first day of their dosing. Subjects who receive IV thrombolysis should not be treated with ASA until 24 hours after thrombolysis therapy, and after a 24-hour neuroimaging procedure shows no evidence of intracranial hemorrhage. Subjects who are hypersensitive to ASA may receive clopidogrel. Combined ASA and dipyridamole (Aggrenox™) is an acceptable alternative to ASA or clopidogrel. Ticlopidine is not an acceptable alternative to ASA because of known hematologic toxicity.

**Anticoagulation:** Anticoagulation with heparin is discouraged. There is no general evidence that heparin is useful in the acute management of stroke subjects for any stroke type. Low-dose subcutaneous unfractionated heparin or low molecular weight heparin may be given after 24 hours, to prevent deep venous thrombosis/pulmonary thromboembolism.

**Blood pressure reduction:** Blood pressure may be treated according to the local standards of the clinical sites and the standards for thrombolysis treatment. According to the principle of this Trial, whose goal is to assess ALB therapy over and above standard-of-care, no restrictions on blood

pressure are in place prior to ALB/placebo therapy. Blood pressure and blood pressure treatments in the acute phase must be recorded.

**Glucose-lowering therapy (insulin):** Currently, there are no completed controlled trials to suggest that lowering glucose in the acute phase results in improvements in clinical outcome among acute stroke subjects. However, subjects with myocardial infarction and critically ill subjects may benefit from aggressive glucose control [77]. Elevated glucose is associated with a higher rate of symptomatic intracranial hemorrhage after thrombolysis therapy [27,104]. Risk factors for hyperglycemia at stroke onset include diabetes mellitus and insular infarction [53]. Plasma glucose and glucose treatments must be recorded.

**Adjuvant transcranial Doppler (TCD) monitoring:** A recent phase II study has suggested that 2 MHz transcranial Doppler ultrasound monitoring of the middle cerebral artery is associated with a higher rate of recanalization and early clinical recovery when combined with thrombolysis treatment [5-7]. Because TCD use is not widespread, there are no restrictions on TCD in this Trial. TCD monitoring/treatment is recorded on the CRF.

### 5.3.2. For Thrombolysis Subjects

**Intravenous (IV) Thrombolytic Therapy:** All subjects who qualify for IV thrombolysis therapy may and should be treated according to the standard of care with IV thrombolysis. In the United States and Canada, IV thrombolysis is approved for use within 3 hours of stroke onset for selected stroke patients. Most guidelines adhere to the study protocol used in the NINDS tPA Stroke Trial, and we recommend that local guidelines be followed.

Recent evidence suggests that IV thrombolysis provides benefit overall to patients out to 4.5 hours from stroke onset and confirms that there is a continuous decline in the magnitude of benefit over time [42]. Criteria for selecting which patients benefit most in any time window are not apparent. Based upon experimental evidence, ALB is most likely to be effective in the reperfusion setting. Patients are not excluded from enrollment in the ALIAS Trial if they are treated with IV thrombolysis beyond the 3-hour window according to the local standards of practice. Because IV thrombolysis use is only approved for use within a 3-hour window by the regulatory agencies in North America, we recommend careful discussion with patients and families, with documentation of consent for treatment beyond the 3-hour window recorded in the medical record.

**Intra-arterial (IA) Thrombolytic Therapy:** IA use of thrombolysis therapy for acute ischemic stroke is not an approved therapy. In general, we discourage the use of IA therapy in the ALIAS Trial because it is not approved by regulatory agencies. While promising and probably safe in selected centers on the basis of the PROACT-II, IMS-I, IMS-II, MERCI and Multi-MERCI studies, it remains unproven that this therapy is better than intravenous thrombolysis alone for acute ischemic stroke patients. Nevertheless, this treatment is offered to patients in the 3- to 5-hour window in selected clinical centers. Additionally, ALB therapy in animal models is most effective in the focal ischemia/reperfusion paradigm, and Albumin Phase I Trial data in humans suggest a dose-response effect in the IV thrombolysis stratum. There are good theoretical reasons to believe that ALB is most effective immediately after reperfusion. Therefore, planned IA therapy at sites where such therapy is considered to be the local standard of care is not considered a contraindication to enrollment in the ALIAS Trial, but the albumin/placebo therapy must be able to be started within 5 hours from symptom

onset and completed within 7 hours from symptom onset. This latter standard is based upon the IMS-I study, where safety of IV+IA thrombolysis was reported [2].

Additionally, we recognize that the acute stroke subject is a dynamic entity, and decision-making in this setting may result in some subjects' being offered IA therapy on a compassionate basis after the subject has been randomized to the ALIAS Trial. Any use of additional IA therapy must be recorded. It is anticipated that this might occur in a very small proportion of subjects because the time window for the treatment is narrow. Moreover, few centers are equipped to offer such therapy. We anticipate that this will not affect our analysis because randomization of subjects should allow for an equal number of IA-treated cases in each of the placebo and ALB treatment arms. Statistical analyses are conducted under the intent-to-treat principle.

One final concern with thrombolysis or device therapy is the stratification of randomization into the Trial by thrombolysis use. Subjects for whom acute IA therapy or device therapy is planned are considered as a part of the thrombolysis stratum. Such subjects are not allowed to receive ALB or placebo until therapy with thrombolysis or with the device has begun. This approach should prevent crossovers between strata. However, rescue IA therapy in the non-thrombolysis stratum may result in crossovers. This is expected to be an extremely infrequent event, if it occurs at all.

# 6. CLINICAL AND LABORATORY EVALUATIONS

## 6.1 Data-Collection Schedule

**ALIAS Part 2 Schedule of Assessments**

| | Phase 1 | Phase 2 | | | | Phase 3 | Phase 4 | Phase 5 | Phase 6 | Phase 7 | Phase 8 | Phase 9 |
|---|---|---|---|---|---|---|---|---|---|---|---|---|
| **Form Name** | **Baseline** | **12 Hrs +/-3h** | **18 Hrs +/-3h** | **24 Hrs +/-6h** | **48 Hrs +/-6h** | **7 Day +/-6h or Discharge*** | **Month 1 +/-7d** | **Month 3 +/-14d** | **Month 6 +/-14d** | **Month 9 +/-14d** | **Month 12 +/-14d** | **End of Study** |
| Screening Log | x | | | | | | | | | | | |
| ALIAS Subject Registration | x | | | | | | | | | | | |
| Subject Eligibility Assessment | x | | | | | | | | | | | |
| OCSP Classification | x | | | | | | | | | | | |
| Stroke Onset / ED Arrival | x | | | | | | | | | | | |
| Medical History | x | | | | | | | | | | | |
| EKG Exam | x | | | x | | | | | | | | |
| EKG Exam - Central Reader | x | | | x | | | | | | | | |
| Baseline Vital Signs | x | | | | | | | | | | | |
| Prior Meds | x | | | | | | | | | | | |
| NIHSS | x | | | x | x | x | | x | | | | |
| CT/MRI Scan | x | | | x | | | | | | | | |
| CT/MRI Scan - Central Reader | x | | | x | | | | | | | | |
| Baseline Laboratory Values | x | | | | | | | | | | | |
| Cardiac Examination | x | | | x | x | x | | | | | | |
| Drug / Therapy Adminstration | | | | x | | | | | | | | |
| Serum Chemistry | | | | x | x | | | | | | | |
| Concomitant Acute Therapies | | (x) | (x) | x | | | | | | | | |
| Weight, Fluid Monitoring & Diuretic Use | | (x) | (x) | (x) | x | | | | | | | |
| Adverse Events | | (x) | (x) | (x) | x | x | x | x | x | x | x | |
| Vital Signs | | (x) | (x) | (x) | (x) | x | | | | | | |
| Hospital Discharge Summary | | | | | | x | | | | | | |
| Concomitant Medications | | (x) | (x) | (x) | (x) | x | x | x | | | | |
| Concomitant Investigations and Procedures | | (x) | (x) | (x) | (x) | x | x | x | | | | |
| Modified Rankin Scale | | | | | | | x | x | x | x | x | |
| Follow-up | | | | | | | x | x | x | x | x | |
| Barthel Index | | | | | | | | x | | | x | |
| EuroQol | | | | | | | | x | | | x | |
| QVSFS | | | | | | | | x | x | x | x | |
| SSQOL v2 | | | | | | | | x | | | | |
| Blindedness Questionnaire-Participant | | | | | | | | x | | | | |
| Blindedness Questionnaire-Investigator | | | | | | | | x | | | | |
| Stroke Etiology | | | | | | x | | | | | | |
| Trailmaking A & B | | | | | | | | x | | | | |
| End of Study | | | | | | | | | | | | x |

* 7 Day or Discharge, whichever comes first

The NIHSS measured immediately before administration of IV or IA thrombolysis (whichever comes first) for those patients being thrombolysed, and immediately before randomization for the non-thrombolysis patients, is considered the baseline NIHSS score

(x) indicates data is collected during this visit but data entered at a later visit.

Vital signs will be collected every 6 hours during the acute hospitalization.

## 6.2 Timing of Evaluations

### 6.2.1 Pre-Randomization Evaluations

These evaluations occur prior to the subject receiving any study interventions. Acute ischemic stroke subjects are best managed on a dedicated stroke unit. Where possible, all subjects should be so

managed. Close attention to fluid balance aiming for euvolemia, prevention of aspiration pneumonia by careful assessment of swallowing function, prevention of deep venous thrombosis and pulmonary thromboembolism, control and investigation of fever, treatment of hyperglycemia and management of blood pressure, and strategies for stroke prevention are critical features of stroke care. Early mobilization and involvement of rehabilitation staff on Day 2 is essential.

Where geographic stroke units are unavailable, a detailed stroke pathway should be followed. Such tools maintain the standard of care by ensuring that routine treatments and investigations are undertaken promptly. Resources for stroke care maps and pathways are available through the American Stroke Association's Get with the Guidelines Program.

### 6.2.2 Evaluations During Hospitalization Following ALB/Placebo Administration

Vital signs are monitored at a minimum of four times daily (q6h) during the entire acute hospitalization. Serum chemistry is collected at 24 (± 6 hours) and 48 hours (± 6 hours). All subjects must have a serum troponin drawn as part of the 24-hour (more frequently if clinically indicated) and 48-hour collections. Fluid balance (IV intake, output) data will be collected at 24 hours (± 6 hours) and 48 hours (± 6 hours). All subjects undergo a follow-up CT scan or brain MRI (including a minimum of diffusion-weighted imaging (DWI), gradient-echo (GRE), FLAIR, and intracranial MR angiography (MRA)) at 24 hours (± 6 hours) from the time of randomization. All subjects also undergo a clinical assessment of neurological and cardiological status at 24 hours (± 6 hours), 48 hours (± 6 hours), and 7 days (± 6 hours) or discharge, whichever comes first. This includes a NIHSS evaluation and standardized cardiac assessment. An EKG should be completed once during the 24-48 hour period. Both the baseline and 24-48h EKG must be reported centrally for review.

### 6.2.3 Intervention Discontinuation Evaluations

ALB infusion may be prematurely terminated if the subject exhibits an anaphylactic reaction or other adverse event thought to be related to ALB. Acute reactions to albumin therapy are rare and no such events occurred during the Albumin Phase 1 study. It is remotely possible that a transfusion reaction consisting of hypotension, urticaria and or laryngospasm in combination or as isolated symptoms may occur. Management of such events should be undertaken according to the best standard of care. Use of antihistamines, fluid and pressor agents and ACLS airway management may be required.

When a subject has been treated with thrombolysis, careful distinction should be made between reactions to thrombolysis and reactions to ALB. It is known that a small proportion (~1%) of stroke patients may suffer acute orolingual angioedema after thrombolysis treatment. This reaction occurs more commonly when patients are taking concurrent angiotensin converting enzyme inhibitors and when infarction involves the insula. (See Section 7.2) Rarely, subjects treated with thrombolysis may develop sudden isolated hypotension, possibly related to release of bradykinin induced by thrombolysis. Management is with volume replacement. Treatment with ALB in this setting may be protective. A call to the ALIAS Medical Monitor in this setting may be helpful.

### 6.2.4 Post-Intervention Evaluations

Subjects are followed for 1 year (12 months) from randomization. At 3 months* (± 14 days) post-randomization, each subject is required to come to the clinic for the determination of clinical outcome of the qualifying stroke – NIHSS, mRS, and BI. Outcomes are determined by a clinical investigator at the site who is certified to administer NIHSS and mRS, **AND** who is blinded to the subject's admission treatment assignment, in-hospital course, and imaging data.

Subjects are also followed by telephone contact at 1 month* (± 7 days), 6 months* (± 14 days), 9 months* (± 14 days), and 12 months* (± 14 days) post-randomization.

At all contacts, data are collected on the subject's current vital status, medical status, living arrangement, mRS, and occurrence of AEs. In addition, all subjects complete the EuroQol [1] at 3 months and 12 months, the Questionnaire to Validate a Stroke-Free Status (QVSFS) [52] at 3, 6, 9, and 12 months, the Stroke-Specific Quality of Life (SS-QOL) [113,114] instruments at 3 months, and Trailmaking A and B at 3 months [125,126,127].

All efforts should be made to ascertain at least the vital status of subjects who are lost to follow-up prior to his/her 12-month follow-up date.

**[*In this Protocol, "3 months" shall be defined as 90 days; "1 month" as 30 days; "6 months" as 180 days; "9 months" as 270 days; and, "12 months" as 365 days.]**

### 6.2.5 Final Evaluations

At the 12-month anniversary date (± 14 days) of each subject's randomization date, the study coordinator, or other designated personnel, contacts the subject via telephone to evaluate mRS, EuroQol, QVSFS, follow-up status, new SAEs and unresolved previously reported SAEs since the last contact, and to complete the end-of-study CRF. If a subject dies prior to that date, the end of study/death CRFs must be completed. The study coordinator, or other designated personnel, should get as much information as possible regarding the date, time, and circumstances of the subject's death.

### 6.3 Off-Intervention Requirements

All subjects are followed using the intent-to-treat principle. Thus, regardless of whether or not a subject has completed the study intervention or received the study drug, all follow-up procedures will be performed according to the standard schedule. The best standard of care applies to all subjects.

## 7. CLINICAL MANAGEMENT OF ADVERSE EXPERIENCES

### 7.1 Complications of ALB Therapy

The major concern with ALB therapy is volume overload. ALB has oncotic properties and therefore draws sodium and water into the intravascular space. Subsequently ALB redistributes in total body water. Administration of ALB at higher doses in the Phase I Trial resulted in a 1.5 to 2 g/dL (15-20 g/L) increase in plasma albumin levels at 4 hours post-administration. The overall incidence of clinical signs of CHF was approximately 13%, but in each case it was mild and the subjects responded to a maximum of 2 doses of furosemide.

Other potential cardiac complications of ALB therapy include atrial arrhythmias (e.g., atrial fibrillation/flutter) and possible myocardial ischemia. Volume infusion may promote atrial wall stress due to stretching, which could lead to atrial arrhythmias. A similar mechanism may occur, causing ischemia. Myocardial wall stress is associated with greater metabolic demand, which may not be available in a subject with coronary stenoses, as many stroke subjects may have. Further, the vast majority of stroke patients are chronic hypertensives, and such patients already tend to have reduced myocardial compliance.

Note that myocardial distress may occur, as shown by evidence of slightly elevated troponins, in up to 20% of ischemic stroke subjects, even without evidence of a true acute coronary syndrome. Thus, it is important to make a clear distinction between subjects who have troponin elevation due to an acute coronary syndrome and troponin elevation without acute coronary syndrome. The latter may be caused by the stroke and is presumed to arise by the same mechanism as seen in acute sub-arachnoid hemorrhage. Stroke may cause a catecholamine surge, possibly due to involvement of the insula, resulting in subendomyocardial damage.

## 7.2 Complications of Thrombolytic Therapy

**Intracranial hemorrhage (ICH):** ICH is a known complication of thrombolytic therapy. Symptomatic ICH was observed in one subject in the Albumin Phase I Trial. Among subjects who are treated with IV thrombolysis, symptomatic ICH occurs in about 6% of subjects. Thrombolytic-related ICH generally occurs in the first 6 hours after thrombolytic therapy. The acute management of intracranial hemorrhage consists of the following:

- Immediate discontinuation of thrombolysis.
- Consider administration of cryoprecipitate, fresh frozen plasma, and platelets; there is no proof that this empiric therapy results in an altered outcome.
- Subjects who concomitantly receive cryoprecipitate, fresh frozen plasma, platelets and ALB may be at particular risk of developing volume overload. These subjects should be managed, as needed, with additional furosemide to prevent the development of acute respiratory failure due to pulmonary edema.

**Systemic hemorrhage:** Systemic hemorrhage is an uncommon complication of thrombolytic therapy, occurring in less than 0.5% of patients. ALB administration was not shown to be a predictor of systemic hemorrhage in the Albumin Phase I Trial. Management is similar to ICH, and precautions against volume overload should be taken as discussed in Section 7.1.

**Orolingual angioedema:** Orolingual angioedema is an uncommon complication of thrombolytic therapy, occurring in 1% or less of subjects treated with thrombolysis. There is no known relationship between ALB therapy and angioedema. Risk factors for angioedema include the premorbid use of ACE inhibitor, β-blockers, and frontoinsular infarction [59]. Management of orolingual angioedema varies by severity. Most angioedema is mild and does not require treatment. Management with antihistamines (50mg IV diphenydramine and 50mg IV rantitidine) may suffice. For more severe cases, 50mg IV hydrocortisone may be administered. Racemic epinephrine is not advised with thrombolysis because of the potential risk of inducing intracerebral hemorrhage. Early consultation with anesthesia for airway management should be considered for more severe cases

**Acute hypotension:** Acute hypotension is an uncommon occurrence with thrombolytic therapy, occurring in 0.4% of patients. It may be related to release of bradykinin. This effect was not observed in the Albumin Phase I Trial. Treatment is generally successful with simple volume expansion, and, therefore, ALB may be protective [123]. In more severe cases, use of inotropic agents (e.g., Dobutamine) may be appropriate.

## 8. ADVERSE EVENTS REPORTING

### 8.1 Definition of Adverse Events

An ***adverse event*** is defined as any untoward event or complication that was not previously identified, or that occurs with greater frequency or severity than previously reported, which occurs during the protocol intervention or during the follow-up period, whether or not considered related to the protocol intervention. Abnormal laboratory findings considered by the reporting physician to be clinically significant are included as adverse events. The investigator, on the basis of his or her clinical judgment and the following definitions, determines the relationship of the adverse event to the protocol intervention as one of the following:

Definite: Causal relationship is certain (i.e., the temporal relationship between drug exposure and the adverse event onset/course is reasonable; there is a clinically compatible response to dechallenge; other causes have been eliminated; and the event must be definitive pharmacologically or phenomenologically, using a satisfactory rechallenge procedure if necessary).

Probable: High degree of certainty for causal relationship (i.e., the temporal relationship between drug exposure and the adverse event onset/course is reasonable; there is a clinically compatible response to dechallenge [rechallenge is not required]; and other causes have been eliminated or are unlikely).

Possible: Causal relationship is uncertain (i.e., the temporal relationship between drug exposure and the adverse event onset/course is reasonable or unknown; dechallenge/rechallenge information is either unknown or equivocal; and while other potential causes may or may not exist, a causal relationship to the study drug does not appear probable).

Unlikely: Not reasonably related, although a causal relationship cannot be ruled out (i.e., while the temporal relationship between drug exposure and the adverse event onset/course does not preclude causality, there is a clear alternate cause that is more likely to have caused the adverse event than the study drug).

Not related: No possible relationship (i.e., the temporal relationship between drug exposure and the adverse event onset/course is unreasonable or incompatible; or a causal relationship to study drug is implausible).

Adverse events are further graded as mild, moderate, severe, life-threatening, or fatal. Adverse events that are non-serious must be followed through the 3-month follow up visit. Only serious adverse events are reported beyond the 3-month follow up visit.

An ***unexpected adverse event*** is any event for which the specificity or severity is not consistent with the current investigator brochure; or, an adverse event for an individual subject that may be adjudicated to be "unexpected" in view of the severity, the timing, and/or the medical context in which it occurs. The study site PI should take all factors carefully into account in deciding whether he/she believes that the event is expected or unexpected.

A ***Serious Adverse Event (SAE)*** is one that results in any of the following outcomes:

- death due to any cause;
- a life-threatening adverse experience (i.e., the subject was at immediate risk of death from the event as it occurred);
- in-patient hospitalization or prolongation of existing hospitalization. (Hospitalizations scheduled before enrollment for an elective procedure or treatment of a pre-existing condition that has not worsened during participation in the study is not considered a serious adverse event);
- a persistent or significant disability/incapacity (i.e., a substantial disruption of one's ability to conduct normal life functions);
- a congenital anomaly/birth defect;
- an important medical event that may not result in death, be life-threatening, or require hospitalization, but may jeopardize the subject and may require medical or surgical intervention to prevent one of the outcomes listed in this definition. Any new diagnosis of cancer (made after study enrollment) is considered an important medical event.

For the ALIAS Trial**, severe, or life-threatening** pulmonary edema or CHF (as defined in Section 8.2) occurring within 48 (± 6) hours of randomization is considered a SAE. All serious adverse events must be followed for the duration of the 12-month trial or until resolution, whichever comes first.

## 8.2 Formal Definitions of Selected Adverse Events

**Acute Congestive Left Heart Failure/Pulmonary Edema.** Acute CHF is defined as pulmonary edema occurring within 48 (± 6) hours of randomization. Pulmonary edema is characterized by all of the following: (a) supplemental oxygen requirement or an increase in oxygen requirement with associated fall in $SaO_2$; (b) elevated respiratory rate and tachycardia; (c) physical examination evidence of pulmonary congestion including elevated jugular venous pressure, presence of hepatojugular reflux, pulmonary auscultatory rales or wheezing.

Although a CXR is not required for diagnosis, if one is done and shows radiological evidence of pulmonary edema it will support the diagnosis.

Acute CHF/pulmonary edema is further classified as:

(i) Asymptomatic – the subject requires no additional therapy. Pulmonary congestion may be only shown on CXR in this case.

(ii) Mild – the subject requires and responds to the single mandated dose of a diuretic and requires supplemental oxygen via nasal prongs only. A single dose of diuretic shall be considered 20mg of intravenous furosemide.
(iii) Moderate – the subject requires more than the mandatory dose of a diuretic but responds and requires supplemental oxygen via nasal prongs only.
(iv) Severe – the subject requires treatment with multiple doses of a diuretic, supplemental oxygen via face mask, or additional problems arise such as cardiac arrhythmias and cardiac telemetry is required.
(v) Life-threatening – the subject requires intubation or non-invasive BiPAP/CPAP to manage significant hypoxemia.

**Acute coronary syndrome (MI).** Elevated **cardiac**-specific CK (total CK-MB mass), troponin-T, or troponin-I greater than the upper limit of normal (ULN) at the clinical site **plus** clinical symptoms or EKG evidence of myocardial injury (ST deviation ≥ 1 mm in 2 contiguous leads or new/previously undocumented Q waves). [120]

**Myocardial injury without acute coronary syndrome -- "enzyme leak".** Elevated cardiac-specific CK (total CK-MB mass), troponin-T, or troponin-I greater than the upper limit of normal (ULN) at the clinical site in the absence of clinical symptoms or EKG evidence of an acute coronary syndrome.

**Acute coronary syndrome - unstable angina.** Clinical symptoms (chest pain, dyspnea) or EKG evidence (ST depression or T wave inversion) of reduced myocardial flow without a significant elevation in cardiac enzymes. [120,121]

**Allergic reaction to ALB.** Type I allergic reaction (anaphylaxis) manifesting as hives, laryngospasm, bronchospasm, hypotension (distributive shock).

**Atrial fibrillation (AF).** Supraventricular tachyarrhythmia characterized by uncoordinated atrial activation with consequent deterioration of atrial mechanical function. [122] Described on the EKG by the replacement of consistent P waves by rapid oscillations or fibrillatory waves that vary in size, shape, and timing, associated with an irregular, frequently rapid ventricular response when atrioventricular (AV) conduction is intact.[122] Any atrial fibrillation occurring post-treatment is considered an adverse event.

**Atrial flutter.** A more organized arrhythmia than AF and is characterized by a saw-tooth pattern of regular atrial activation called flutter (*f*) waves on the EKG, particularly visible in leads II, III, and aVF (inferior limb lead on a 12-lead EKG), without an isoelectric baseline between deflections.[122] Any atrial flutter occurring post-treatment shall be considered an adverse event.

**Bowel pseudo-obstruction.** Failure of peristalsis of the large bowel with associated dilataton. May result in sepsis and toxic megacolon.

**Constipation.** Hard, infrequent bowel motions. May be functionally defined as no bowel motion for 5 or more days.

**Deep vein thrombosis (DVT) or pulmonary thrombo-embolism (PE).** DVT and PE are spectrums of the same disease. A DVT is a thrombotic occlusion of a large vein in the leg, pelvis, abdomen, or arm. A PE is a thrombotic occlusion of a pulmonary artery, most often due to a venous embolus from a DVT.

**Hemicraniectomy.** Removal of a large ovoid region of the skull performed with an associated duroplasty to allow infracted brain to swell without causing rostral-caudal deterioration and death.

**Sudden vascular death.** Deaths are categorized as sudden cardiovascular if they are unexpected, rapidly follow symptoms, and are attributable to a cardiovascular or unexplained cause. [122]

**Pulmonary hypertension.** (A) Measured (e.g., Swann-Ganz catheter) or estimated (echocardiography) right ventricular end-diastolic pressure greater than 10 mm Hg; or (B) Measured or estimated using echocardiography, a peak right ventricular systolic pressure > 25mm Hg.

**Renal failure.** Renal failure shall be defined as renal dysfunction requiring artificial renal replacement therapy (hemodialysis or peritoneal dialysis).

**Renal insufficiency.** Renal insufficiency shall be defined as renal dysfunction, shown by a 20% increase in serum creatinine from baseline, not requiring renal replacement therapy.

**Urinary tract infection (lower).** Bacterial infection of the bladder, often as a complication of Foley catheter placement. Should be suspected based upon the presence of white cells and/or nitrites in the urine and proven by culture.

**Respiratory failure.** Hypoxemia or hypercarboxemia requiring mechanical ventilation (either intubation or non-invasive with BiPAP/CPAP).

**Respiratory insufficiency.** Hypoxemia or hypercarboxemia that can be managed without mechanical ventilation (either intubation or non-invasively with BiPAP/CPAP).

**Acute treatment-emergent hypertension.** Elevated blood pressure occurring in the first 48 hours of 220 systolic or 120 mmHg diastolic, OR an increase of 20% in either systolic blood pressure or mean arterial pressure over baseline values.

**Increased intracranial pressure.** Raised ICP (> 25 cm $H_2O$) requiring interventional treatment (e.g., hemicraniectomy, placement of an external ventricular drain).

**Neurological deterioration.** Any increase in NIH stroke scale of 4 points or more, over the baseline value, occurring at any point between randomization and 48 hours.

**Stroke death.** Death from stroke (neurological death attributed to the stroke) within 7 days (± 6 hours) or during hospitalization for the qualifying stroke.

**Recurrent stroke**. A new stroke occurring in a new vascular territory, separated in time from the initial stroke event by 48 hours or more.

**Progression of stroke.** Worsening of the same stroke symptoms that defined the initial stroke event, involving deficits attributable to the same vascular territory and occurring within 48 hours of the original stroke event.

**Pneumonia.** The following criteria define pneumonia:
(i) hypoxemia ($S_aO_2 < 90\%$)
(ii) pulmonary air space disease defined by clinical examination and/or chest X-ray
(iii) fever (temperature > 38$^o$C)

Two of three are required. Cough, dyspnea, pleuritic chest pain and delirium are additional supportive symptoms/signs. Pneumonia is confirmed by culture of a single bacterial or viral organism or by clinical resolution of symptoms with antimicrobial treatment and absence of a good alternate diagnosis (e.g., pulmonary embolus, atelectasis, congestive heart failure).

**Hypotension.** Hypotension is defined as the syndrome of low blood pressure with SBP < 85 mmHg.

**Treatment-emergent hypotension.** Hypotension associated with clinical/radiographic/laboratory evidence of end-organ dysfunction. This could include, but is not limited to:
- delirium or reduced level of consciousness, or focal neurological signs indicating brain dysfunction
- symptoms and signs of an acute coronary syndrome indicating cardiac dysfunction
- significantly reduced urine output (< 15cc/h) indicating renal dysfunction
- significantly elevated liver enzymes indicating shock liver/hepatic dysfunction

*(NOTE: Extra care should be paid to blood pressure monitoring in subjects on ACE inhibitors.)*

**Hypercarbia**. Hypercarbia (in the context of respiratory failure/insufficiency) is defined as an arterial pCO2 > 45 mmHg.

## 8.3 Definition of Intracranial Hemorrhage (ICH)

ICH is any bleeding inside the cranial vault. Intracranial is further subdivided into **intracerebral hemorrhage** (bleeding into the parenchyma of brain), **intraventricular hemorrhage** (bleeding into the ventricular system), **subarachnoid hemorrhage** (bleeding into the subarachnoid space), **subdural hematoma** (bleeding into the subdural space), and **epidural hematoma** (bleeding into the epidural space). Any intracranial hemorrhage diagnosis and typing must be confirmed by neuroimaging. Intracranial hemorrhage is further classified as symptomatic or asymptomatic and by the Pessin criteria used in the CASES studies (PH-2, PH-1, HI-2, HI-1) [124].

**Treatment-related *symptomatic ICH*** refers to the occurrence of intracranial hemorrhage within 24 ± 6 hours of randomization, proven by neuroimaging (MRI or CT), and associated with a deterioration in neurological status. In the investigator's opinion, the hemorrhage must be thought to be the primary cause of the subject's deterioration. For example, a subject with a malignant MCA infarct and HI-2 (confluent petechial hemorrhage) would be deemed to have deteriorated from the malignant edema, rather than the HI-2 hemorrhage, and therefore, the hemorrhage would be classified as asymptomatic. It is expected that symptomatic ICH related to thrombolysis primarily consists of parenchymal hematoma hemorrhagic infarction types 1 and 2 (H1 or H2) OR subarachnoid hemorrhage OR intraventricular hemorrhage. In addition, any subject with a parenchymal hematoma types 1 or 2 (PH1 or PH2) on the 24 ± 6 hour CT scan and an increase in the NIHSS score (measured at 24 ± 6 hours) over the baseline of 4 or more points is considered to have symptomatic ICH even if the investigator does not judge the two events to be related. Any hemorrhage, however, that an investigator deems to be related is treated as symptomatic.

***Asymptomatic intracranial hemorrhage*** refers to the occurrence of intracranial hemorrhage within 24 (± 6) hours of randomization that is not associated with worsening in the subject's neurological status.

**Systemic hemorrhage.** Hemorrhage other than intracranial hemorrhage. Includes gastrointestinal hemorrhage, retroperitoneal hemorrhage, epistaxis, and other bleeding. Does not include minor bleeding from the intravenous injection sites, abrasions, decubitus ulcers, etc.

## 8.4 Reporting of Adverse Events and Serious Adverse Events

In order to assure prompt and complete reporting of adverse events or complications, all non-serious AEs occurring through the 3-month follow-up visit and all serious AEs occurring through the 12-month follow-up visit are recorded in the WebDCU™ system.

For each AE, the clinical site staff records the event in WebDCU™, providing relevant information such as the AE description, and seriousness. For SAEs, they also must provide the date of onset and resolution, severity, suspected relationship to stroke, suspected relationship to study drug, and suspected relationship to thrombolysis, and a narrative description of actions taken as a result of the SAE.

## 8.5 Review and Expedited Reporting of Serious Adverse Events

All serious AEs must be reported by the site into the WebDCU™ system within 24 hours of first knowledge of the SAE. Additionally, all current study data for that particular subject must be entered to allow for timely review by the MSMs. Upon entry of a serious AE, WebDCU™ triggers notification of the SAE to the Project Manager. The narrative section of the SAE report is reviewed by an AMM, and if the narrative is satisfactory the SAE is forwarded to the MSMs.

The MSMs conduct independent blinded reviews of all SAEs entered into WebDCU™. Should any medical reviewer need additional subject data to conduct his review, those data may be

accessed on the WebDCU™. The MSMs submit their opinions on whether the AE was a) serious, b) unexpected, and c) related to the study drug within 72 hours of notification of the SAE. The MSMs may contact the site for more information or discussion. If two of the three parties involved with the reporting and/or reviewing of the SAE (i.e., site investigator and either of the MSMs, both MSMs, or all three) believe the AE is serious, study drug-related (possibly, probably or definitely), and unexpected, the SAE is considered to require expedited reporting.

When it is confirmed that an event requires expedited reporting, the Project Manager contacts the site with instructions for completion of a MedWatch form, or other safety reporting forms. An electronic copy of the MedWatch form is sent through the WebDCU™ system and the site staff enters the required information. Following the site's completion of the MedWatch form, or other safety reporting form, DCU submits the MedWatch form, or other safety reporting forms, to the FDA. DCU also distributes this information to the DSMB (through the NINDS DSMB Liaison), Health Canada (through the Canadian Coordinating Center), and the clinical site PIs. Each clinical site PI is then responsible for reporting to its own IRB/REB.

## 8.6 Follow-Up Reporting of Serious Adverse Events

After the submission of the initial MedWatch report, or other safety report, the clinical site staff is responsible for obtaining any follow-up information about the SAE. All follow-up information should be actively sought by the clinical site and must be submitted to the DCU as soon as the information becomes available. The DCU distributes the follow-up MedWatch report, or other safety report, to all parties receiving the initial MedWatch report, or other safety report.

## 8.7 Additional Safety Review

A summary of all adverse events and a summary of all MedWatch reports, or other safety reports, submitted for the previous year will be included in the annual reports submitted to the FDA and Health Canada, and reports submitted to the DSMB. In addition, reports of all adverse events are submitted in aggregate to the DSMB on a regular basis.

On a monthly basis, the unblinded Study Statistician at the DCU submits a report of the partially unblinded safety data to the DSMB for review. Details of the safety monitoring guidelines are provided in Section 8.8, and Section 10.7.2.

# ALIAS Study SAE Reporting and Monitoring Flowchart

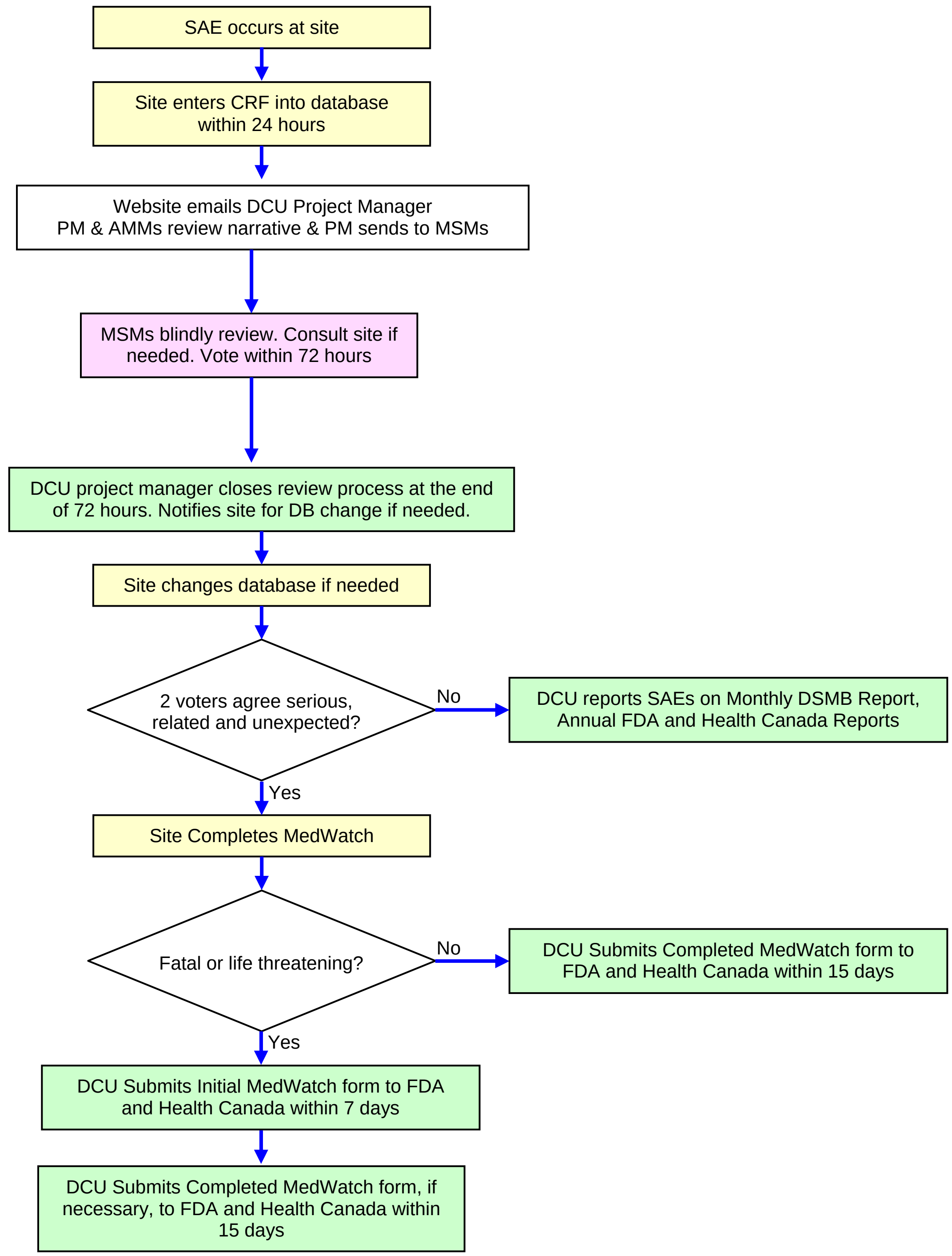

### 8.8 Monitoring of Specific Adverse Events

Incidences of the following specific adverse events are monitored by the Trial EC in a blinded manner and by the unblinded Trial personnel and the DSMB by treatment group throughout the study:

- Neurological deterioration as assessed by the investigator of an increase of 4 points or more on the NIHSS anytime within 48 (± 6) hours of randomization
- Neurological death from stroke during hospitalization or within 7 days (± 6 hours) of randomization, whichever is shorter
- Recurrent stroke within 30 (± 7) days of randomization
- Atrial fibrillation within 48 (± 6) hours of randomization
- Severe or life threatening pulmonary edema or CHF within 48 (± 6) hours of randomization
- Shortness of breath within 48 (± 6) hours of randomization
- Symptomatic ICH within 24 (± 6) hours of randomization
- Asymptomatic ICH as assessed by the CT scan at 24 (± 6) hours
- Death from any cause within 30 (± 7) days of randomization
- Death from any cause within 90 (± 14) days of randomization

For each Monthly Closed Safety Report to the DSMB, the partially blinded study statistician calculates the percentages (= (number of subjects with event / number of subjects at risk of event) x 100) within the treatment group and the relative risk (with 95% CI) of subjects with each of the specific events listed above for each of the two treatment groups (A vs B). In addition, using the Poisson distribution, the event rate per person time in each treatment group along with its 95% CI are provided. Non-overlap in the confidence intervals of ALB and placebo groups is flagged in the monthly report to the DSMB.

## 9. CRITERIA FOR INTERVENTION DISCONTINUATION

There are only three ways that a randomized subject may prematurely discontinue participation in the Trial prior to completing the 12-month follow-up assessment: (1) the subject dies; (2) the subject withdraws consent; or (3) the subject becomes lost to follow-up (LTFU). Every study subject has the right to withdraw voluntarily from the study at any time for any reason without prejudice to his/her future medical care by the physician or at the institution. For the occasional participant who withdraws consent, the date and reason for consent withdrawal must be documented.

All efforts should be made to contact the subject who fails to show up at the scheduled 3-month follow-up visits since the assessments made at this visit provide the primary outcome measure for the Trial. For the 1-, 6-, 9- and 12-month follow-up telephone contact, the Study Coordinator, or designee, must make at least five attempts over the course of two weeks. When all methods have been tried and have failed, the subject is coded as lost to follow-up in the End-of-Study CRF.

The procedure to be followed at the time of premature termination is: 1) to check for the development of adverse events; and 2) to complete the End-of-Study form with explanation of why the participant is withdrawing or has withdrawn. In case of death of a subject, additional data on the date and causes of death (underlying and contributing) must be collected.

# 10. STATISTICAL CONSIDERATIONS

## 10.1 Overview

The primary analysis for the Trial is to test the hypothesis of superiority of ALB therapy over placebo in acute ischemic stroke subjects, adjusting for the effects of thrombolysis and baseline stroke severity The study is a parallel two-arm design with concurrent controls where eligible patients are randomized in 1:1 ratio to either the ALB group or the saline group. The primary outcome for efficacy/futility is the proportion of subjects within each group with favorable outcome as defined as mRS score of 0 or 1 and/or NIHSS score of 0 or 1 at 3 months from randomization. Interim analyses for overwhelming efficacy and futility as well as for safety are incorporated into the Trial.

## 10.2 Outcomes

### 10.2.1 Primary Efficacy Outcome

The primary outcome measure is the favorable outcome defined as either NIHSS score of 0 or 1 and/or mRS score of 0 or 1 at 3 months from randomization. Interim analyses for futility and efficacy are based on the proportion of subjects with the favorable outcome.

The choice of this outcome measure is due to the putative effect of ALB as a neuroprotective agent. The conventional outcome measure of clinical outcome for acute ischemic stroke treatment is the functional outcome as measured by the mRS at 3 months from randomization or baseline. We concur that any treatment for acute ischemic stroke should yield good functional outcome as measured by mRS score of 0 or 1 and sometimes 2. However, alternatively, if ALB therapy is an effective neuroprotective agent, we would expect to see good neurological outcome as measured by the NIHSS score of 0 or 1. Therefore, we selected the composite outcome where either the good functional or good neurological outcome would qualify as evidence of a beneficial ALB treatment effect.

### 10.2.2 Secondary Outcomes

The following are outcome measures to be evaluated as supportive evidence (or lack thereof) of the ALB treatment effect: (1) mRS score at 3 months; (2) mRS score at 12 months; (3) NIHSS score at 24 hours; (4) NIHSS score at 3 months; (5) BI score at 3 months; (6) EuroQol scores at 3 months; (7) EuroQol scores at 12 months; (8) SSQOL at 3 months; (9) stroke-free status as assessed by QVSFS at 12 months; (10) ASPECTS score by CT scan at 24 (± 6) hours; (11) proportion of subjects who do not have symptomatic ICH within 24 (± 6) hours; and (12) cognition as assessed by Trailmaking A and B at 3 months. The specific treatment of each of these outcome measures is detailed in the Statistical Analysis Plan.

### 10.2.3 Safety Outcomes

Several specific adverse events are monitored throughout the study (see Section 8.8). However, death from any cause within 30 (± 7) days of randomization is evaluated statistically during

and at the end of the Trial. The details of the analysis are provided in Sections 10.4.3. We propose to use the 30-day mortality as the primary safety outcome because of the observed differences between the treatment groups during that period in the Part 1 of the ALIAS Trial. Furthermore, the causes of death during that period are suspected to be more related to the study treatment compared to deaths occurring post-30 days from randomization. Clearly, the seriousness of this event requires close monitoring; therefore, we plan to conduct frequent interim analyses of this outcome (see Section 10.7.2).

### 10.3 Sample Size and Accrual

The total sample size for the effect size of 10% absolute difference between the ALB and control groups in the proportion of subjects with favorable outcomes [or 25% increase in relative benefit (RB)] assuming the control group's proportion of 40%, and Type I and Type II error probabilities of 0.025 (one-sided) and 0.10, respectively, is 1,100. We assume a group sequential design with 3 interim analyses for overwhelming efficacy and concurrently, for futility based on O'Brien and Fleming [1] boundaries (after about ¼, ½, and ¾ of the subjects complete the 3-month follow-up assessments). See Section 10.7 for the details on interim analysis.

This sample size will allow testing of the effect of interaction between the study treatment and thrombolytic treatment. We will be able to detect an interaction effect of 20% absolute difference between the thrombolysis and non-thrombolysis strata in the magnitude of the treatment effect with 80% power at two-sided alpha of 0.10. Note that the interaction effect will be tested only upon completion of the study and not at each of the interim analyses.

The ITT principle will be applied to the analysis. With a total sample size of 1,100, if we observe no more than 5% lost-to-follow-up, we should have no less than approximately 87% power to detect a 10% treatment effect (i.e., the total sample size is inflated by a factor of 1.11[32]). Assuming that the control group's proportion of favorable outcome is 40%, a 10% increase in the proportion in the ALB group translates to a relative benefit ratio of 1.25.

### 10.4 Data Analyses

#### 10.4.1 Analysis of Primary Efficacy Outcome

The primary analysis for the Trial is to test the hypothesis of superiority of ALB therapy over placebo in acute ischemic stroke subjects, adjusting for the effects of thrombolysis and stroke severity. The analysis model for the primary efficacy outcome is:

$$E(Y) = \alpha + \beta_1 x_1 + \beta_2 x_2 + \beta_3 x_3 + \beta_4 x_1 x_2 , \qquad (1)$$

where Y= primary clinical outcome (0=unfavorable, 1=favorable), $\beta_i$ is the regression coefficient, $x_1$= treatment assignment (0=saline, 1=ALB), $x_2$= thrombolysis treatment (0=no, 1=yes), and $x_3$= baseline NIHSS score. The last term on the right hand side of the equation represents the study drug by thrombolysis interaction effect.

We will first test the interaction term. If it is not statistically significant at $\alpha$=0.10 or if it is statistically significant but the interaction is not qualitative (i.e., if study drug effect in both thrombolysis strata are in the same direction), then we will test:

$$\beta_1 = 0 \text{ vs } \beta_1 > 0,$$

using the model:

$$E(Y) = \alpha + \beta_1 x_1 + \beta_2 x_2 + \beta_3 x_3 \,. \quad (2)$$

Otherwise, the primary efficacy analysis will be based only on subjects who receive thrombolytic treatment using the model:

$$E(Y) = \alpha + \beta_1 x_1 + \beta_3 x_3. \quad (3)$$

For this test, we anticipate approximately 880 subjects in the thrombolysis stratum based on the observed recruitment ratio of 4 thrombolysis subjects to every one non-thrombolysis subject who were enrolled in the Part 1 of the Trial. If this ratio holds in Part 2, we will have insufficient sample size to test the non-thrombolysis group with adequate power; however, the data will be evaluated as exploratory analysis.

The primary analysis of the main efficacy outcome (whether the interaction is significant or not) is tested using the generalized linear model with log link function. It is tested at the one-sided alpha level of 0.025. PROC GENMOD procedure of SAS® v9 is used to obtain the test statistics and the results [130]. In addition, relative benefit ratio of ALB compared to saline and its 95% confidence interval are calculated.

As the primary analysis, all efficacy outcome measures will be analyzed under the ITT principle. Under this principle, the evaluable sample includes all subjects who are randomized, and each subject will be analyzed according to the treatment group to which they were randomly assigned and in the stratum to which they were classified at the time of randomization.

The primary analysis does not adjust for the clinical site effect due to the large number of sites (approximately 60-80). As a secondary analysis, we assess the ALB therapy effect adjusting for the effect of clinical sites. One such analysis is conducted with a generalized linear model containing treatment (ALB or placebo), thrombolysis, and baseline NIHSS as a fixed effect, clinical site as a random effect (due to the large number of sites), and treatment-by-site interaction effect as a random effect.

Other secondary analyses of the primary outcome at the end of the study include generalized linear model analysis adjusting for a variety of covariates that are deemed clinically or prognostically important (in addition to thrombolysis treatment and baseline NIHSS score). Prior to these analyses, univariate analyses of covariates are conducted to determine their inclusion in the multivariable models. Some of the variables suspected to be associated with the outcome measures are: age at baseline, African American race, serum glucose at baseline, systolic blood pressure at baseline, ASPECTS score from baseline CT scan, history of MI, and time from symptom onset to treatment. The usual verification of variable and model assumptions (e.g., normality and homoscedasticity) and goodness of fit assessments accompany each analysis.

Subgroup analyses are also planned, assuming sufficient numbers of subjects are enrolled in the subgroups. These analyses involving covariates are tested at the two-sided alpha level of 0.01 in order to control nominally for the inflation of Type I error. The details of these secondary analyses on the primary outcome measure are provided in the Statistical Analysis Plan.

### 10.4.2 Analysis of Secondary Efficacy Outcomes

In addition to the simple descriptive analysis of outcome measures, including correlation among them, a series of secondary efficacy outcomes are evaluated. The analyses for the secondary efficacy outcomes are conducted with SAS® Software System Version 9. Some outcome measures may have substantial departure from normal distribution, even after transformation, in which case nonparametric methods may be considered; however, with a sample size of approximately 1,100, we anticipate this to be rare problem. As these analysis results are treated as supportive evidence (or lack thereof) of the ALB treatment effect rather than conclusive evidence, the significance of each test is determined at the two-sided alpha level of 0.01 in order to control nominally for inflation of Type I error. The details of the secondary outcomes analyses are provided in the Statistical Analysis Plan.

### 10.4.3 Analysis of Safety Outcome

The Cox proportional hazards model is used to analyze time to death within 30 days from randomization. The analysis adjusts for the effects of thrombolysis treatment, age (continuous), and baseline NIHSS score (continuous). The sample size of 1,100 yields an anticipated maximum number of events of 170 based on the 3.5-year subject accrual time at, on average, 1 subject per day (which is the rate observed around the time of enrollment suspension of Part 1 of the Trial) under the Type I and II error probabilities of 0.01 and 0.10, respectively.

### 10.4.4 Meta Analysis

The effectiveness or lack thereof of ALB will be determined solely from the analysis of the ALIAS Trial Part 2 primary outcome data. Additionally, at the end of Part 2 of the trial, as exploratory analyses, data from Parts 1 and 2 of the Trial will be combined for meta analyses. The two approaches we will adopt are: (1) analysis of weighted summary statistics from the two cohorts/strata of Part 1 and Part 2 of the trial using the inverse normal method; and, (2) analysis of individual data from the three cohorts. For the latter approach, we will adjust for the study and cohort/strata effect when we combine all data, and adjust for the study effect when we analyze the subgroups of thrombolysis and non-thrombolysis subjects.

## 10.5 Analysis Samples

As the primary analysis, all futility and efficacy outcome measures specified in Sections 10.2.1 and 10.2.2 are analyzed under the ITT principle. For the outcomes measured at 3 months, the ITT sample includes all subjects who are randomized regardless of whether they actually received the study drug (ALB or placebo), and regardless of whether or not they had a 3-month outcome assessments. If they are missing, their data are imputed. See Section 10.6 for handling of missing data.

As secondary analyses, the 3-month outcome assessments may be analyzed excluding those who are lost-to-follow-up (LTFU) only if the proportion of LTFU is greater than 5%. If it is less than 5%, we anticipate that the analysis results with the excluded sample will differ minimally from the ITT sample. We also anticipate the possibility, albeit very small, of subjects receiving the incorrect treatment. This is considered a major protocol violation (PV). Nevertheless, as a tertiary analysis of

outcome measures, we will repeat the analysis using this "as treated" sample upon completion of the Trial.

Any randomized subjects who received at least 20% of their weight-based dose of the study drug are included in the safety analysis sample.

### 10.6 Handling of Missing Data

Under the ITT principle, all subjects who are randomized are included in the analysis. Therefore, missing data, especially in the outcome measures, can be problematic. For the primary outcome analysis, missing data are imputed by assuming the worst-case scenario. In other words, subjects with a missing score for both of the assessments (mRS and NIHSS) at 3 months are assigned an unfavorable outcome. Of course, missing data due to death are scored as unfavorable outcome. If a subject is missing one of the assessments and the observed outcome is unfavorable (i.e., NIHSS score $\geq 2$ or mRS score $\geq 2$), the subject is assigned an unfavorable outcome. On the other hand, if the subject is missing one of the assessments and the observed outcome is favorable (i.e., NIHSS score of 0 or 1 or mRS score of 0 or 1), the subject is assigned a favorable outcome, per the definition of the primary outcome.

As a secondary analysis approach, the primary efficacy outcome is imputed using other methods (e.g., last observation carried forward from the 1 month mRS and discharge NIHSS assessments; hot-decking; logistic regression), and sensitivity analysis is conducted. Similar imputation methods are employed for secondary outcomes that are binary. For measurements that are on a continuous scale (e.g., SSQOL), the multiple imputation method is employed. Missing covariate data are imputed using either the multiple imputation or regression method, if needed.

It is possible and probable that missing outcome data may not be missing at random, in which case the imputation methods to be employed may yield biased results. However, based on our previous experiences with clinical trials of acute stroke, we anticipate minimal lost-to-follow-up for the 3-month assessment (less than 5%) of the primary outcome. The concern, therefore, is on the outcomes assessed at 1 year. All efforts must be put forth to ensure near-complete follow-up, in particular with occurrences of deaths and recurrences of stroke events. A forum, such as the ALIAS Trial Newsletter and Trial website, is established by the Executive Committee to allow communications among the clinical sites to share what methods are most successful in minimizing lost-to-follow-up cases.

### 10.7 Data Monitoring

#### 10.7.1 Statistical Stopping Guidelines for Overwhelming Efficacy and Futility

Three interim analyses are planned for assessment for evidence of overwhelming efficacy as well as for futility. For overwhelming efficacy, we adopt the alpha spending function approach [59], and for futility, the beta spending function approach [74]. For both overwhelming efficacy and futility, O'Brien and Fleming-type stopping guidelines are used [72]. In other words, the difference between the ALB and placebo groups must be very large early on in the study to reject the null hypothesis for overwhelming efficacy, and similarly, the difference must be very small early on in the study to "reject" the alternative hypothesis to declare futility of observing statistical differences in the treatment effect between the two groups.

The tentative stopping criteria, calculated with EAST® 5 software (Cytel Software, 2006), based on the 3 planned interim analyses (or a total of 4 analyses) at equal intervals during the study are provided in Table 10.7.1. These values are updated using EAST® 5 software. Interim analyses for consideration of stopping the study for overwhelming efficacy or for futility are based solely on the analysis of the primary outcome adjusted for thrombolysis and baseline severity. The timing and frequency of interim analyses may be changed upon DSMB request, and the spending function approach allows for such flexibility.

Table 10.7.1. Stopping boundaries for overwhelming efficacy and futility based on one-sided $\alpha=0.025$

| Analysis | Reject $H_0$ (for overwhelming efficacy) if p-value is less than: | Reject $H_1$ (for futility) if p-value is greater than: |
|---|---|---|
| 1 | 0.0000 | 0.7940 |
| 2 | 0.0015 | 0.2710 |
| 3 | 0.0092 | 0.0805 |
| Final | 0.0220 | 0.0220 |

Each interim analysis is conducted ignoring the interaction effect between thrombolysis and study treatment. The rationale is: (1) we expect we will have insufficient power to detect any strong qualitative interaction effect at any interim analysis; and (2) in the absence of statistical evidence of strong qualitative interaction effect, we plan to use model (2) in Section 10.4.1 as the primary efficacy analysis. However, at each interim analysis, conditional power of the test for interaction effect given the data to date and current trend will be calculated for evaluation by the DSMB.

### 10.7.2 Statistical Stopping Guidelines for Safety

Because of concerns about excess deaths in one group over the other in Part 1 of the ALIAS Trial, we have established one set of statistical safety stopping guidelines based on mortality within 30 days from randomization as shown in Table 10.7.2.

Table 10.7.2 Suggested approximate stopping guidelines for safety.

| | | Maximum split in # of death between the two groups to yield LL of 99% CI on RR < 1.0 | | | |
|---|---|---|---|---|---|
| Approximate N at interim monitoring* | Total # death* | Group A | Group B | Unadjusted RR** | LL of 99% CI for the unadjusted RR |
| 100 | 15 | 3 | 12 | 4.00 | 0.959 |
| 200 | 30 | 9 | 21 | 2.33 | 0.981 |
| 300 | 45 | 16 | 29 | 1.81 | 0.925 |
| 400 | 60 | 22 | 38 | 1.73 | 0.969 |
| 500 | 75 | 29 | 46 | 1.59 | 0.952 |
| 600 | 90 | 35 | 55 | 1.57 | 0.986 |
| 700 | 105 | 42 | 63 | 1.50 | 0.977 |
| 800 | 120 | 49 | 71 | 1.45 | 0.972 |

| | | | | | |
|---|---|---|---|---|---|
| 900 | 135 | 55 | 80 | 1.45 | 0.998 |
| 1000 | 150 | 62 | 88 | 1.42 | 0.994 |
| 1100 | 165 | 69 | 96 | 1.39 | 0.991 |

* Assumes 15% overall 30-day death rate.
** In practice, the RR will be estimated adjusting for thrombolysis treatment, age, and baseline NIHSS, and its LL of the 99% CI will be used for stopping criterion.

The above stopping guideline is based on the total number of deaths observed within 30 days. Hence, the first interim monitoring point occurs after a total of 15 deaths are observed, which should translate to when approximately 100 subjects have had the 30-day follow up, assuming a 15% overall 30-day death rate. The 3-to-12 split of the 15 deaths yields an unadjusted RR of 4.0 with the lower limit (LL) of its one-sided 99% CI at just below 1.0. The split that is more extreme (i.e., 2-to-13, 1-to-14, or 0-to-15) will yield an LL of greater than 1.0.

Subsequent interim monitoring point occurs after every additional 15 deaths (within 30 days) are observed.

Because thrombolysis treatment, age, and baseline NIHSS are strong prognostic indicators of death, in practice, we will estimate the RR adjusting for these covariates, and we would become alerted if the LL of its one-sided 99% CI begins to approach 1.0. The DSMB will evaluate this and other pertinent data to consider stopping the trial. It would be the DSMB's prerogative to consider all information made available to them in their decision to continue or stop the trial regardless of the above suggested statistical stopping guideline.

#### 10.7.3 Data and Safety Monitoring Board (DSMB) Reports

The DCU generates a monthly safety monitoring report as well as a comprehensive statistical report semi-annually to the DSMB. The monthly reports contain updated baseline and safety information (including statistical interim monitoring results as appropriate) by treatment-group code (A and B) but not the name of the actual treatment. If the DSMB wishes to completely unblind itself from the partially unblinded reports, the Chair of the DSMB may request the NINDS Liaison to the DSMB to open the sealed envelope that contains the treatment-code identification. This allows the DSMB to unblind itself immediately upon its decision to do so.

The semi-annual reports are distributed to the DSMB prior to their planned semi-annual meetings. The comprehensive report includes compiled data on enrollment (expected and actual), demographic and baseline characteristics, eligibility and protocol violations, safety data, concomitant medications and procedures, and data quality (e.g., timeliness of data entry, and number of data clarification requests generated and resolved). The comprehensive report that coincides in timing with the planned interim analysis also contains the results of the analysis for overwhelming efficacy and futility. The content of the reports is partially unblinded with treatment groups identified as “A” or “B”. If the DSMB wishes to be completely unblinded for these comprehensive reports, a sealed treatment identification envelope will be provided to the NINDS DSMB Liaison; this envelope can be opened at the discretion of the DSMB.

## 11. DATA COLLECTION AND MANAGEMENT OVERVIEW

Data management is handled by the DCU, which is housed in the Department of Biostatistics, Bioinformatics and Epidemiology at the Medical University of South Carolina (MUSC). All activities are conducted in coordination with the clinical sites, the University of Miami and University of Calgary.

Case Report Forms (CRFs) have been developed by the DCU with input from the Executive Committee. An electronic copy of the CRFs is made available to the clinical sites prior to initiation of the study to be used as worksheets for capturing data for the Trial.

The clinical site staff is responsible for timely entry of required data into the database via the WebDCU™ System. The WebDCU™ is a user-friendly menu-driven system with built-in warnings and rules to facilitate the data collection process and ensure sufficient quality control. Upon enrollment of an eligible subject, his/her demographics and randomization code must be entered into the database **within 8 hours** of unsealing of the subject's study medication kit (see Section 4.3.3). When a SAE is discovered, the adverse event information along with all of the data currently collected for that particular subject must be entered into the database **within 24 hours** of the discovery (see Section 8.5). All other study data must be entered **within 5 days** of each subject's completion of each phase of the study (see Section 6.2). The details of the data entry requirements for the clinical site staff are provided in the MoP.

The study database, WebDCU™ System, has been developed in Microsoft SQL Server based on the approved CRFs. This system allows for a web-based data entry and management. The data are captured and entered (single keyed) at the clinical sites via the web interface. The data are managed (including data queries) by the DCU using a secured ALIAS Trial website. During the design of the database, automated consistency checks and data validation rules were programmed to check for potential data errors, including missing required data, data out of pre-specified range, data conflicts and disparities within and among the CRFs. All validation rules are outlined in the Data Management Plan generated by the DCU.

The validation procedure is implemented in two stages. First, automated data-checks flag the items that fail pre-programmed logic checks. The Study Coordinator sees on the data entry screen a validation error and he/she is requested to address it. His/her choices are to: (1) correct the entry immediately; (2) correct the entry at a later time; or (3) if the entered data are correct, dismiss the rule violation. This last option is not allowed for gross logic discrepancies such as a violation of a skip pattern. Any changes made on the website have a full audit trail. Secondly, for some checks that are more complicated, such as inter-CRF data-checks, additional validation takes place using the web interface. This process involves the running of the consistency-check (validation rules) program that was prepared during the development of the database. All data items that fail the programmed consistency checks are queried via the data clarification request (DCR) process initiated by the DCU. The DCRs are generated, communicated between DCU and the clinical sites, and resolved on the secured study website.

In addition to the study database, the DCU provides the clinical site staff access (via password) to a standard set of web-enabled tools, including subject visit calendar, subject accrual reports, CRF completion status, and outstanding DCR status pertaining to their respective sites. These tools allow the staff to receive regular updates on overall study status, new external information relevant to the Trial, Committee meetings calendar, etc.

Backup tapes of data collected on the WebDCU™ System are generated on a daily basis. The backup software (logical) security policy has three main components: (1) antiviral protection with

McAfee Enterprise Edition to protect all servers and workstations from infection with virus definitions, updated on a daily basis; (2) restricted access with password policies; and (3) three levels of firewall protection – MUSC dual-layered firewall (hardware) protection between the University and outside computer systems and the firewall (software) on web servers with limited access. Furthermore, the WebDCU™ System uses SSL encryption methods that allow a secured Internet transfer.

## 12. HUMAN SUBJECTS

### 12.1 Institutional Review Board (IRB)/Research Ethics Board (REB) Review and Informed Consent

This protocol and the informed consent document and any subsequent modifications are reviewed and approved by the IRB/REB or ethics committee responsible for oversight of the study. A signed consent form must be obtained from the subject. For subjects who cannot consent for themselves, a legally authorized representative, or person with power of attorney, may sign the consent form. The consent form describes the purpose of the study, the procedures to be followed, and the risks and benefits of participation. A copy of the consent form must be given to the subject, the legally authorized representative, or the person with power of attorney; and this fact must be documented in the subject's record.

### 12.2 Subject Confidentiality

All laboratory specimens, evaluation forms, reports, video recordings, and other records that leave the site are identified only by the Randomization Code Number to maintain subject confidentiality. All records are kept in a locked file cabinet. All computer entry and networking programs are done using SIDs only. Clinical information is not released without written permission of the subject, except as necessary for monitoring by IRB/REB, the FDA, Health Canada, the NINDS, the OHRP, the sponsor, or the sponsor's designee.

All study investigators at the clinical sites must ensure that the confidentiality of personal identity and all personal medical information of study participants is maintained at all times. Additionally, the U.S. clinical sites must follow privacy obligations to study participants under the Health Insurance Portability and Accountability Act (HIPAA). Analogous federal legislation in Canada (Personal Information Protection and Electronic Documents Act – PIPEDA), and provincial legislation where applicable, must be followed. On the CRFs and other study documents or image materials submitted to the DCU, the subjects are identified only by study identification codes.

Personal medical information may be reviewed for the purpose of verifying data recorded in the CRF by the site monitors. Other properly authorized persons, such as the regulatory authorities, may also have access to these records. Personal medical information is always treated as confidential.

### 12.3 Study Modification/Discontinuation

The study may be modified or discontinued at any time by the NINDS, the sponsor, the OHRP, the FDA, Health Canada, or other government agencies as part of their duties to ensure that research subjects are protected. Additionally, the IRB/REB at any site may discontinue the trial at that site, but only at that site, should it be deemed necessary.

## 12.4 Site Monitoring

To ensure monitoring responsibilities are performed to the fullest extent possible, an elite team of regionally based, industry experienced independent contractor clinical research monitors perform on site data verification for the trial. A target of no less than 40% of the clinical data submitted to the ALIAS database are verified against source documents at the performance sites prior to finalization of the database. Complete source data verification took place on data submitted for all first subjects enrolled at each site in Part 1 of the ALIAS Trial. For Part 2 of the ALIAS Trial, the same verification will be done for the first subject enrolled at any new sites. For subsequent subjects, a checklist of key outcome and safety data variables for 100% source monitoring has been developed based on the trials safety and efficacy endpoints. The safety and efficacy variables on the monitoring checklist represent no less than 20% of data in the database. The remaining 20% of source monitored data include: 100% of deaths and 100% of serious adverse events and all DCU-requested source data reviews based on the per-subject evaluation of safety parameters defined in the protocol. All data monitored on site are verified for accuracy and thoroughness using the most appropriate source documents for all subjects. In addition, 100% of subjects enrolled are monitored for the presence of signed consent and HIPAA and PIPED documentation.

Additional on-site monitoring verification includes: ongoing evaluation of the adequacy of site facilities and staff, site recruitment, subject randomization, drug accountability, the presence of regulatory documents, and specific review of documents and data as requested by the DCU staff. The initial performance monitoring visit to a site takes place after the first subject is enrolled. Thereafter, it is expected that each site will be monitored at least twice a year. Sites are evaluated in an ongoing manner by site monitors and the DCU to determine if there is a need to monitor more frequently or more thoroughly.

During the monitoring visit, any omissions and corrections to data submitted to the database are noted and queries are generated by the monitor on site or within 48 hours via the WebDCU™ system.

In addition to completing a site questionnaire, each hospital facility and its research team may be visited by DCU personnel, or its designee, prior to study initiation. If this visit is made, it is to verify the likelihood of successful subject recruitment, appropriateness of the research team composition, and quality of the patient care facilities. Additionally, the DCU staff or monitor verifies that the site understands and is able to perform its responsibilities for maintaining all regulatory documents required for the trial.

The ALIAS Trial Part 1 initial Investigators' Meeting served as the initiation of all site investigators and staff able to attend. If a center joined the trial after the Investigators' Meeting has occurred, one or both of the AMMs, the on-site monitor, DCU staff, or some combination thereof, initiated the site after the site had been successfully screened by the Executive Committee. For the ALIAS Trial Part 2, there will be a re-training meeting to familiarize the clinical sites with the changes adopted for Part 2 of the trial. This meeting will include a re-training module consisting of instruction and a test. For clinical site staff unable to attend the re-training meeting, the re-initiation of the site may take the form of personal visits by any combination of those mentioned above, or through a webcast session conducted by one or both of the AMMs for the protocol, or participation in a web based re-training module (with accompanying test). At the time of a personal visit, those conducting the visit verify the presence and completeness of regulatory documentation at the performance site, and perform a review of the study protocol, electronic data entry worksheets and study drug accountability process with the appropriate clinical site personnel.

The close-out monitoring visit by a monitor takes place at the completion of subject enrollment at the performance site. At that visit, the monitor again reviews the presence of a regulatory file and verifies documents for currency and completion as directed by the DCU. A final accounting of all study drug and supplies takes place, and any remaining study drug and study drug kits are destroyed or returned as directed by the trial's sponsor. Sites are instructed in the record retention of all trial documents. Principal Investigators are directed to close the trial and issue a final report to the IRB / REB. Finally, any additional special consideration for the auditing of any additional safety issues are made during this final monitoring visit.

Clinical Research Monitor training took place just prior to the Investigators' Meeting for Part 1 of the ALIAS Trial, and the clinical research monitors will be included in the re-training meeting planned for Part 2 of the ALIAS Trial.. The DCU staff manages the assignment of monitors to performance sites, the coordination of monitoring visits, and provides support to monitors while they are in the field.

## 13. PUBLICATION AND PRESENTATION POLICY

The ALIAS Trial Publications Subcommittee of the Executive Committee, consisting of Drs. Ginsberg, Hill, Moy, and Palesch, coordinates and approves all writing and presentation activities of the Trial. The publication and presentation policy is detailed in the ALIAS Trial Publication and Presentation Policy document (included in the MoP).

Briefly, for manuscripts, the manuscript for the primary results of the Trial should be submitted for publication to an appropriate peer-reviewed journal within approximately 6 months of the database-freeze (i.e., complete and cleaned data) of the 3-month follow-up data of all Trial participants. The manuscript on the 1-year follow-up data should be submitted for publication within approximately 6 months of the completion of the Trial. The Publications Subcommittee establishes a writing group to be headed by the Study Chair (Dr. Ginsberg) who oversees the writing of these major manuscripts. Other major manuscripts (such as the one describing the study design) may also be generated in this manner.

For all other publications, a writing group for each manuscript is established. Any ALIAS Trial investigators (clinicians as well as study coordinators) wishing to write a manuscript must first submit to the Publications Subcommittee a brief description of the manuscript content (hereafter referred to as the manuscript proposal) according to the template provided in the ALIAS Trial Publication and Presentation Policies document. The Subcommittee reviews the proposal in a timely manner, and if the proposal is approved, it is disseminated to all Trial investigators to encourage those who have similar interests in the topic to participate in the Writing Group. The Publications Subcommittee appoints the Writing Group members. Each Writing Group must include at least one statistician from the DCU. Prior to submission to a journal, the Writing Group must submit to the Subcommittee the penultimate draft of the manuscript for input and approval. Generally, the Writing Group determines the authorship and its order, and the Publications Subcommittee provides the final approval. The Subcommittee has the right to arbitrate any disputes regarding authorship and its decision is binding.

All abstracts must be based on active manuscript proposals or submitted or published papers. For abstracts, the same procedure (including decision on authorship) as manuscripts applies. In addition, the final draft of the abstract must be submitted to the Publications Subcommittee at least 2 weeks prior to the abstract deadline.

## 14. ANCILLARY STUDIES POLICY

The Executive Committee follows NINDS policy on ancillary studies as described in http://www.ninds.nih.gov/funding/research/clinical_research/ancillary.htm. The details of the process to initiate and implement ancillary studies are provided in the ALIAS Trial Ancillary Studies Policy (included in the MoP).

Briefly, any ALIAS Trial investigator(s) wishing to conduct ancillary studies must submit in writing to the Executive Committee a proposal that includes an outline of the protocol. A meeting of the Executive Committee is convened to discuss the proposal, and each member of the Executive Committee votes to approve or disapprove the proposal. The key criteria for the evaluation are scientific merit, relevance to the major goals of the ALIAS Trial, and its impact on the conduct of the ALIAS Trial. If, and only if, the proposal is approved by the Executive Committee, it is forwarded to the DSMB members who also vote for approval or disapproval. Only upon approval by the Executive Committee and the DSMB may the investigator(s) submit the proposal to potential funding source(s), if necessary.

## 15. DATA-SHARING PLAN

The Executive Committee follows NIH policy on data-sharing, as described in http://grants2.nih.gov/grants/policy/data_sharing/data_sharing_guidance.htm. Upon completion of the ALIAS Trial, the public use database is prepared by stripping any and all personal identifiers. The public use database, consisting of several data files, should contain: (1) baseline and demographic characteristics; (2) outcomes assessments; (3) CT/MRI data; (4) concomitant medications and procedures; and (5) adverse events. Each data file is made available as a formatted SAS dataset or other electronic format. The data files are distributed along with the data dictionary and a brief instruction ("Readme") file. These data files will be made available to the public only after all major manuscripts (including secondary analysis papers) of the Trial are accepted for publication in peer-reviewed journals.

Anyone wishing to access the data may do so by completing a data-sharing agreement and data request form and submitting it to an external data archiving unit chosen by the NINDS. The detailed plan is provided in the ALIAS Trial Data Sharing Policy and Procedures.

## 16. REFERENCES

1. EuroQol--a new facility for the measurement of health-related quality of life. The EuroQol Group. Health Policy 1990; 16:199-208.

2. Combined intravenous and intra-arterial recanalization for acute ischemic stroke: the Interventional Management of Stroke Study. Stroke 2004; 35:904-911.

3. Aichner FT, Fazekas F, Brainin M, Polz W, Mamoli B, Zeiler K. Hypervolemic hemodilution in acute ischemic stroke: the Multicenter Austrian Hemodilution Stroke Trial (MAHST). Stroke 1998; 29:743-749.

4. Albers GW, Zivin JA, Choi DW. Ethical standards in phase I trials of neuroprotective agents for stroke therapy. Stroke 1998; 29:1493-1494.

5. Alexandrov AV. Ultrasound identification and lysis of clots. Stroke 2004; 35 (Suppl 1): 2722-2725.

6. Alexandrov AV, Demchuk AM, Burgin WS, Robinson DJ, Grotta JC. Ultrasound-enhanced thrombolysis for acute ischemic stroke: phase I. Findings of the CLOTBUST trial. J Neuroimaging 2004; 14:113-117.

7. Alexandrov AV, Wojner AW, Grotta JC. CLOTBUST: design of a randomized trial of ultrasound-enhanced thrombolysis for acute ischemic stroke. J Neuroimaging 2004; 14:108-112.

8. Aronowski J, Strong R, Grotta JC. Combined neuroprotection and reperfusion therapy for stroke. Effect of lubeluzole and diaspirin cross-linked hemoglobin in experimental focal ischemia. Stroke 1996; 27:1571-1576.

9. Asplund K. Haemodilution for acute ischaemic stroke. Cochrane Database Syst Rev 2002;CD000103.

10. Bar-Or D, Rael LT, Lau EP, Rao NK, Thomas GW, Winkler JV, Yukl RL, Kingston RG, Curtis CG. An analog of the human albumin N-terminus (Asp-Ala-His-Lys) prevents formation of copper-induced reactive oxygen species. Biochem Biophys Res Commun 2001; 284:856-862.

11. Belayev L, Alonso OF, Huh PW, Zhao W, Busto R, Ginsberg MD. Posttreatment with high-dose albumin reduces histopathological damage and improves neurological deficit following fluid percussion brain injury in rats. J Neurotrauma 1999; 16:445-453.

12. Belayev L, Busto R, Zhao W, Clemens JA, Ginsberg MD. Effect of delayed albumin hemodilution on infarction volume and brain edema after transient middle cerebral artery occlusion in rats. J Neurosurg 1997; 87:595-601.

13. Belayev L, Liu Y, Zhao W, Busto R, Ginsberg MD. Human albumin therapy of acute ischemic stroke : marked neuroprotective efficacy at moderate doses and with a broad therapeutic window. Stroke 2001; 32:553-560.

14. Belayev L, Pinard E, Nallet H, Seylaz J, Liu Y, Riyamongkol P, Zhao W, Busto R, Ginsberg MD. Albumin therapy of transient focal cerebral ischemia: in vivo analysis of dynamic microvascular responses. Stroke 2002; 33:1077-1084.

15. Belayev L, Saul I, Busto R, Danielyan K, Vigdorchik A, Khoutorova L, Ginsberg MD. Albumin treatment reduces neurological deficit and protects blood-brain barrier integrity following acute intracortical hematoma in the rat. Stroke 2005; 36:326-331.

16. Belayev L, Saul I, Huh PW, Finotti N, Zhao W, Busto R, Ginsberg MD. Neuroprotective effect of high-dose albumin therapy against global ischemic brain injury in rats. Brain Res 1999; 845:107-111.

17. Belayev L, Zhao W, Pattany PM, Weaver RG, Huh PW, Lin B, Busto R, Ginsberg MD. Diffusion-weighted magnetic resonance imaging confirms marked neuroprotective efficacy of albumin therapy in focal cerebral ischemia. Stroke 1998; 29:2587-2599.

18. Bogaert YE, Rosenthal RE, Fiskum G. Postischemic inhibition of cerebral cortex pyruvate dehydrogenase. Free Radic Biol Med 1994; 16:811-820.

19. Busto R, Belayev L, Saul I, Curbelo K, Khoutorova L, Belayev A, Zhang Y, Ginsberg MD. A double-injection model of intracerebral hematoma in the rat: effect of albumin treatment. Soc Neurosci Abstr 2002;#390.8.

20. Chan PH. Reactive oxygen radicals in signaling and damage in the ischemic brain. J Cereb Blood Flow Metab 2001; 21:2-14.

21. Ciccone A, Sterzi R, Crespi V, Defanti CA, Pasetti C. Thrombolysis for acute ischemic stroke: the patient's point of view. Cerebrovasc Dis 2001; 12:335-340.

22. Cole DJ, Drummond JC, Osborne TN, Matsumura J. Hypertension and hemodilution during cerebral ischemia reduce brain injury and edema. Am J Physiol 1990; 259:H211-H217.

23. Cole DJ, Drummond JC, Patel PM, Nary JC, Applegate RL. Effect of oncotic pressure of diaspirin cross-linked hemoglobin (DCLHb) on brain injury after temporary focal cerebral ischemia in rats. Anesth Analg 1996; 83:342-347.

24. Cole DJ, Schell RM, Drummond JC, Pryzbelski RJ, Marcantonio S. Focal cerebral ischemia in rats: effect of hemodilution with alpha- alpha cross-linked hemoglobin on brain injury and edema. Can J Neurol Sci 1993; 20:30-36.

25. Cole DJ, Schell RM, Drummond JC, Reynolds L. Focal cerebral ischemia in rats. Effect of hypervolemic hemodilution with diaspirin cross-linked hemoglobin versus albumin on brain injury and edema. Anesthesiology 1993; 78:335-342.

26. Curry S, Mandelkow H, Brick P, Franks N. Crystal structure of human serum albumin complexed with fatty acid reveals an asymmetric distribution of binding sites. Nat Struct Biol 1998; 5:827-835.

27. Demchuk AM, Morgenstern LB, Krieger DW, Linda CT, Hu W, Wein TH, Hardy RJ, Grotta JC, Buchan AM. Serum glucose level and diabetes predict tissue plasminogen activator-related intracerebral hemorrhage in acute ischemic stroke. Stroke 1999; 30:34-39.

28. Dirnagl U, Simon RP, Hallenbeck JM. Ischemic tolerance and endogenous neuroprotection. Trends Neurosci 2003; 26:248-254.

29. Emerson TE. Unique features of albumin: a brief review. Crit Care Med 1989; 17:690-694.

30. Endres M, Dirnagl U. Ischemia and stroke. Adv Exp Med Biol 2002; 513:455-73.:455-473.

31. European Ad Hoc Consensus Group. Neuroprotection as initial therapy in acute stroke. Third Report of an Ad Hoc Consensus Group Meeting. Cerebrovasc Dis 1998; 8:59-72.

32. Friedman LM, Furberg CD, DeMets DL. Fundamentals of Clinical Trials. New York: Springer-Verlag; 1998.

33. Galis Z, Ghitescu L, Simionescu M. Fatty acids binding to albumin increases its uptake and transcytosis by the lung capillary endothelium. Eur J Cell Biol 1988; 47:358-365.

34. Ghitescu L, Fixman A, Simionescu M, Simionescu N. Specific binding sites for albumin restricted to plasmalemmal vesicles of continuous capillary endothelium: receptor-mediated transcytosis. J Cell Biol 1986; 102:1304-1311.

35. Ginsberg MD. Neuroprotection in brain ischemia -- an update -- Parts I and II. Neuroscientist 1995; 1:95-103-164-175.

36. Ginsberg MD, Belayev L, Zhao W, Liu Y, Busto R. The neuroprotective efficacy of human serum albumin therapy in focal and global cerebral ischemia and traumatic brain injury. In Krieglstein J (ed). Pharmacology of Cerebral Ischemia 2000. Stuttgart: Medpharm Scientific Publishers; 2000.

37. Gladstone DJ, Black SE, Hakim AM. Toward wisdom from failure: lessons from neuroprotective stroke trials and new therapeutic directions. Stroke 2002; 33:2123-2136.

38. Goslinga H, Eijzenbach V, Heuvelmans JH, van der Laan de Vries, Melis VM, Schmid-Schonbein H, Bezemer PD. Custom-tailored hemodilution with albumin and crystalloids in acute ischemic stroke. Stroke 1992; 23:181-188.

39. Grotta J. Neuroprotection is unlikely to be effective in humans using current trial designs. Stroke 2002; 33:306-307.

40. Grotta JC, Pettigrew LC, Allen S, Tonnesen A, Yatsu FM, Gray J, Spydell J. Baseline hemodynamic state and response to hemodilution in patients with acute cerebral ischemia. Stroke 1985; 16:790-795.

41. Gum ET, Swanson RA, Alano C, Liu J, Hong S, Weinstein PR, Panter SS. Human serum albumin and its N-terminal tetrapeptide (DAHK) block oxidant-induced neuronal death. Stroke 2004; 35:590-595.

42. Hacke W, Donnan G, Fieschi C, Kaste M, von Kummer R, Broderick JP, Brott T, Frankel M, Grotta JC, Haley EC, Jr., Kwiatkowski T, Levine SR, Lewandowski C, Lu M, Lyden P, Marler JR, Patel S, Tilley BC, Albers G, Bluhmki E, Wilhelm M, Hamilton S. Association of outcome with early stroke treatment: pooled analysis of ATLANTIS, ECASS, and NINDS rt-PA stroke trials. Lancet 2004; 363:768-774.

43. Halliwell B. Albumin--an important extracellular antioxidant? Biochem Pharmacol 1988; 37:569-571.

44. Halliwell B, Gutteridge JM. The antioxidants of human extracellular fluids. Arch Biochem Biophys 1990; 280:1-8.

45. He P, Curry FE. Albumin modulation of capillary permeability: role of endothelial cell [$Ca^{2+}$]i. Am J Physiol 1993; 265:H74-H82.

46. Hemodilution in Stroke Study Group. Hypervolemic hemodilution treatment of acute stroke. Results of a randomized multicenter trial using pentastarch. The Hemodilution in Stroke Study Group. Stroke 1989; 20:317-323.

47. Heros RC, Korosue K. Hemodilution for cerebral ischemia. Stroke 1989; 20:423-427.

48. Hou ST, MacManus JP. Molecular mechanisms of cerebral ischemia-induced neuronal death. Int Rev Cytol 2002; 221:93-148.:93-148.

49. Hu ML, Louie S, Cross CE, Motchnik P, Halliwell B. Antioxidant protection against hypochlorous acid in human plasma. J Lab Clin Med 1993; 121:257-262.

50. Huh PW, Belayev L, Zhao W, Busto R, Saul I, Ginsberg MD. The effect of high-dose albumin therapy on local cerebral perfusion after transient focal cerebral ischemia in rats. Brain Res 1998; 804:105-113.

51. Italian Acute Stroke Study Group. Haemodilution in acute stroke: results of the Italian haemodilution trial. Lancet 1988; 1:318-321.

52. Jones WJ, Williams LS, Meschia JF. Validating the Questionnaire for Verifying Stroke-Free Status (QVSFS) by neurological history and examination. Stroke 2001; 32:2232-2236.

53. Kagansky N, Levy S, Knobler H. The role of hyperglycemia in acute stroke. Arch Neurol 2001; 58:1209-1212.

54. Keaney JFJ, Simon DI, Stamler JS, Jaraki O, Scharfstein J, Vita JA, Loscalzo J. NO forms an adduct with serum albumin that has endothelium-derived relaxing factor-like properties. J Clin Invest 1993; 91:1582-1589.

55. Koch-Weser J, Sellers EM. Binding of drugs to serum albumin (first of two parts). N Engl J Med 1976; 294:311-316.

56. Koch-Weser J, Sellers EM. Drug therapy. Binding of drugs to serum albumin (second of two parts). N Engl J Med 1976; 294:526-531.

57. Koroshetz WJ, Moskowitz MA. Emerging treatments for stroke in humans. Trends Pharmacol Sci 1996; 17:227-233.

58. Korosue K, Heros RC, Ogilvy CS, Hyodo A, Tu YK, Graichen R. Comparison of crystalloids and colloids for hemodilution in a model of focal cerebral ischemia. J Neurosurg 1990; 73:576-584.

59. Lan KKG, DeMets DI. Design and analysis of group sequential tests based on type-I error spending rate function. Biometrika 1987; 74:149-154.

60. Lang JD, Jr., Figueroa M, Chumley P, Aslan M, Hurt J, Tarpey MM, Alvarez B, Radi R, Freeman BA. Albumin and hydroxyethyl starch modulate oxidative inflammatory injury to vascular endothelium. Anesthesiology 2004; 100:51-58.

61. Lee SH, Heros RC, Mullan JC, Korosue K. Optimum degree of hemodilution for brain protection in a canine model of focal cerebral ischemia. J Neurosurg 1994; 80:469-475.

62. Lennihan L, Mayer SA, Fink ME, Beckford A, Paik MC, Zhang H, Wu Y-C, Klebanoff LM, Raps EC, Solomon RA. Effect of hypervolemic therapy on cerebral blood flow after subarachnoid hemorrhage. A randomized controlled trial. Stroke 2000; 31:383-391.

63. Levy ML, Rabb CH, Zelman V, Giannotta SL. Cardiac performance enhancement from dobutamine in patients refractory to hypervolemic therapy for cerebral vasospasm. J Neurosurg 1993; 79:494-499.

64. Little JR, Slugg RM, Latchaw JPJ, Lesser RP. Treatment of acute focal cerebral ischemia with concentrated albumin. Neurosurgery 1981; 9:552-558.

65. Liu Y, Belayev L, Zhao W, Busto R, Belayev A, Ginsberg MD. Neuroprotective effect of treatment with human albumin in permanent focal cerebral ischemia: histopathology and cortical perfusion studies. Eur J Pharmacol 2001; 428:193-201.

66. Marler JR, Walker MD. Progress in acute stroke research. Stroke 1998; 29:1491-1492.

67. Matsui T, Sinyama H, Asano T. Beneficial effect of prolonged administration of albumin on ischemic cerebral edema and infarction after occlusion of middle cerebral artery in rats. Neurosurgery 1993; 33:293-300.

68. Mori K, Arai H, Nakajima K, Tajima A, Maeda M. Hemorheological and hemodynamic analysis of hypervolemic hemodilution therapy for cerebral vasospasm after aneurysmal subarachnoid hemorrhage. Stroke 1995; 26:1620-1626.

69. Nadal A, Fuentes E, Pastor J, McNaughton PA. Plasma albumin is a potent trigger of calcium signals and DNA synthesis in astrocytes. Proc Natl Acad Sci U S A 1995; 92:1426-1430.

70. Nadal A, Fuentes E, Pastor J, McNaughton PA. Plasma albumin induces calcium waves in rat cortical astrocytes. Glia 1997; 19:343-351.

71. National Institute of Neurological Disorders and Stroke rt-PA Stroke Study Group. Tissue plasminogen activator for acute ischemic stroke. N Engl J Med 1995; 333:1581-1587.

72. O'Brien PC, Fleming TR. A multiple testing procedure for clinical trials. Biometrics 1979; 35:549-556.

73. Ohtaki M, Tranmer BI. Role of hypervolemic hemodilution in focal cerebral ischemia of rats. Surg Neurol 1993; 40:196-206.

74. Pampallona S, Tsiatis AA, Kim KM. Interim monitoring of group sequential trials using spending functions for the Type I and Type II error probabilities. Drug Information Journal 2001; 35:1113-1121.

75. Perez-Trepichio AD, Furlan AJ, Little JR, Jones SC. Hydroxyethyl starch 200/0.5 reduces infarct volume after embolic stroke in rats. Stroke 1992; 23:1782-1790.

76. Peters TJr. All About Albumin. Biochemistry, Genetics, and Medical Applications. San Diego: Academic Press; 1996.

77. Pittas AG, Siegel RD, Lau J. Insulin Therapy for Critically Ill Hospitalized Patients: A Meta-analysis of Randomized Controlled Trials. Arch Intern Med 2004; 164:2005-2011.

78. Powers KA, Kapus A, Khadaroo RG, He R, Marshall JC, Lindsay TF, Rotstein OD. Twenty-five percent albumin prevents lung injury following shock/resuscitation. Crit Care Med 2003; 31:2355-2363.

79. Reinhart WH, Nagy C. Albumin affects erythrocyte aggregation and sedimentation. Eur J Clin Invest 1995; 25:523-528.

80. Rodeheffer RJ. Measuring plasma B-type natriuretic peptide in heart failure: good to go in 2004? J Am Coll Cardiol 2004; 44:740-749.

81. Rodriguez de Turco EB, Belayev L, Liu Y, Busto R, Parkins N, Bazan NG, Ginsberg MD. Systemic fatty acid responses to transient focal cerebral ischemia: influence of neuroprotectant therapy with human albumin. J Neurochem 2002; 83:515-524.

82. Sacco RL. Risk factors and outcomes for ischemic stroke. Neurology 1995; 45:S10-S14.

83. Samsa GP, Matchar DB, Goldstein L, Bonito A, Duncan PW, Lipscomb J, Enarson C, Witter D, Venus P, Paul JE, Weinberger M. Utilities for major stroke: results from a survey of preferences among persons at increased risk for stroke. Am Heart J 1998; 136:703-713.

84. Scandinavian Stroke Study Group. Multicenter trial of hemodilution in acute ischemic stroke. I. Results in the total patient population. Stroke 1987; 18:691-699.

85. Scandinavian Stroke Study Group. Multicenter trial of hemodilution in acute ischemic stroke. Results of subgroup analyses. Stroke 1988; 19:464-471.

86. Schaller B, Graf R. Cerebral ischemia and reperfusion: the pathophysiologic concept as a basis for clinical therapy. J Cereb Blood Flow Metab 2004; 24:351-371.

87. Schnitzer JE. gp60 is an albumin-binding glycoprotein expressed by continuous endothelium involved in albumin transcytosis. Am J Physiol 1992; 262:H246-H254.

88. Schnitzer JE, Oh P. Antibodies to SPARC inhibit albumin binding to SPARC, gp60, and microvascular endothelium. Am J Physiol 1992; 263:H1872-H1879.

89. Schnitzer JE, Sung A, Horvat R, Bravo J. Preferential interaction of albumin-binding proteins, gp30 and gp18, with conformationally modified albumins. Presence in many cells and tissues with a possible role in catabolism. J Biol Chem 1992; 267:24544-24553.

90. Schurr A, Miller JJ, Payne RS, Rigor BM. An increase in lactate output by brain tissue serves to meet the energy needs of glutamate-activated neurons. J Neurosci 1999; 19:34-39.

91. Schurr A, Payne RS, Miller JJ, Rigor BM. Brain lactate is an obligatory aerobic energy substrate for functional recovery after hypoxia: further in vitro validation. J Neurochem 1997; 69:423-426.

92. Schurr A, Payne RS, Miller JJ, Rigor BM. Brain lactate, not glucose, fuels the recovery of synaptic function from hypoxia upon reoxygenation: an in vitro study. Brain Res 1997; 744:105-111.

93. Schurr A, Payne RS, Miller JJ, Rigor BM. Glia are the main source of lactate utilized by neurons for recovery of function posthypoxia. Brain Res 1997; 774:221-224.

94. Siesjo BK. Review. Cell damage in the brain: A speculative synthesis. J Cereb Blood Flow Metab 1981; 1:155-185.

95. Slyter H. Ethical challenges in stroke research. Stroke 1998; 29:1725-1729.

96. Smith C, Halliwell B, Aruoma OI. Protection by albumin against the pro-oxidant actions of phenolic dietary components. Food Chem Toxicol 1992; 30:483-489.

97. Solomon NA, Glick HA, Russo CJ, Lee J, Schulman KA. Patient preferences for stroke outcomes. Stroke 1994; 25:1721-1725.

98. Sort P, Navasa M, Arroyo V, Aldeguer X, Planas R, Ruiz-del-Arbol L, Castells L, Vargas V, Soriano G, Guevara M, Gines P, Rodes J. Effect of intravenous albumin on renal impairment and mortality in patients with cirrhosis and spontaneous bacterial peritonitis. N Engl J Med 1999; 341:403-409.

99. Strand T. Evaluation of long-term outcome and safety after hemodilution therapy in acute ischemic stroke. Stroke 1992; 23:657-662.

100. Suarez JI, Shannon L, Zaidat OO, Suri MF, Singh G, Lynch G, Selman WR. Effect of human albumin administration on clinical outcome and hospital cost in patients with subarachnoid hemorrhage. J Neurosurg 2004; 100:585-590.

101. Sudlow CL, Warlow CP. Comparable studies of the incidence of stroke and its pathological types: results from an international collaboration. International Stroke Incidence Collaboration. Stroke 1997; 28:491-499.

102. Sundt TMJ, Waltz AG, Sayre GP. Experimental cerebral infarction: modification by treatment with hemodiluting, hemoconcentrating, and dehydrating agents. J Neurosurg 1967; 26:46-56.

103. Tabernero A, Medina A, Sanchez-Abarca LI, Lavado E, Medina JM. The effect of albumin on astrocyte energy metabolism is not brought about through the control of cytosolic $Ca^{2+}$ concentrations but by free-fatty acid sequestration. Glia 1999;25:1-9.

104. Tanne D, Kasner SE, Demchuk AM, Koren-Morag N, Hanson S, Grond M, Levine SR. Markers of increased risk of intracerebral hemorrhage after intravenous recombinant tissue plasminogen activator therapy for acute ischemic stroke in clinical practice: the Multicenter rt-PA Stroke Survey. Circulation 2002; 105:1679-1685.

105. Tomita H, Ito U, Tone O, Masaoka H, Tominaga B. High colloid oncotic therapy for contusional brain edema. Acta Neurochir Suppl (Wien ) 1994; 60:547-9.

106. Tone O, Ito U, Tomita H, Masaoka H, Tominaga B. High colloid oncotic therapy for brain edema with cerebral hemorrhage. Acta Neurochir Suppl (Wien ) 1994; 60:568-70.

107. Trigatti BL, Gerber GE. A direct role for serum albumin in the cellular uptake of long-chain fatty acids. Biochem J 1995; 308:155-159.

108. Tsacopoulos M, Magistretti PJ. Metabolic coupling between glia and neurons. J Neurosci 1996; 16:877-885.

109. Tu YK, Heros RC, Karacostas D, Liszczak T, Hyodo A, Candia G, Zervas NT, Lagree K. Isovolemic hemodilution in experimental focal cerebral ischemia. Part 2: Effects on regional cerebral blood flow and size of infarction. J Neurosurg 1988; 69:82-91.

110. Wasil M, Halliwell B, Hutchison DC, Baum H. The antioxidant action of human extracellular fluids. Effect of human serum and its protein components on the inactivation of alpha 1- antiproteinase by hypochlorous acid and by hydrogen peroxide. Biochem J 1987; 243:219-223.

111. Wayner DD, Burton GW, Ingold KU, Locke S. Quantitative measurement of the total, peroxyl radical-trapping antioxidant capability of human blood plasma by controlled peroxidation. The important contribution made by plasma proteins. FEBS Lett 1985; 187:33-37.

112. Williams GR. Incidence and Characteristics of Total Stroke in the United States. BMC Neurol 2001; 1:2.

113. Williams LS, Weinberger M, Harris LE, Biller J. Measuring quality of life in a way that is meaningful to stroke patients. Neurology 1999; 53:1839-1843.

114. Williams LS, Weinberger M, Harris LE, Clark DO, Biller J. Development of a stroke-specific quality of life scale. Stroke 1999; 30:1362-1369.

115. Wood JH, Simeone FA, Fink EA, Golden MA. Hypervolemic hemodilution in experimental focal cerebral ischemia. Elevation of cardiac output, regional cortical blood flow, and ICP after intravascular volume expansion with low molecular weight dextran. J Neurosurg 1983; 59:500-509.

116. Yanaka K, Camarata PJ, Spellman SR, McDonald DE, Heros RC. Optimal timing of hemodilution for brain protection in a canine model of focal cerebral ischemia. Stroke 1996; 27:906-912.

117. Yenari MA. Heat shock proteins and neuroprotection. Adv Exp Med Biol 2002; 513:281-99.:281-299.

118. Zhao Y, Marcel YL. Serum albumin is a significant intermediate in cholesterol transfer between cells and lipoproteins. Biochemistry 1996; 35:7174-7180.

119. Zoellner H, Hofler M, Beckmann R, Hufnagl P, Vanyek E, Bielek E, Wojta J, Fabry A, Lockie S, Binder BR. Serum albumin is a specific inhibitor of apoptosis in human endothelial cells. J Cell Sci 1996; 109:2571-2580.

SUBSEQUENT ADDITIONS TO LITERATURE CITATIONS

120. Zimmerman J, Fromm R, Meyer D, Boudreaux A, Wun CC, Smalling R, Davis B, Habib G, Roberts R. Diagnostic marker cooperative study for the diagnosis of myocardial infarction. *Circulation.* 1999;99:1671-7.

121. Braunwald E, Antman EM, Beasley JW, Califf RM, Cheitlin MD, Hochman JS, Jones RH, Kereiakes D, Kupersmith J, Levin TN, Pepine CJ, Schaeffer JW, Smith EE, 3rd, Steward DE, Theroux, P, Gibbons RJ, Alpert JS, Eagle KA, Faxon DP, Fuster V, Gardner TJ, Gregoratos G, Russell RO, Smith SC, Jr. ACC/AHA guidelines for the management of patients with unstable angina and non-ST-segment elevation myocardial infarction: executive summary and recommendations. A report of the American College of Cardiology/American Heart Association task force on practice guidelines (committee on the management of patients with unstable angina). *Circulation*. 2000;102:1193-209.

122. Kuller LH. Sudden death--definition and epidemiologic considerations. *Prog Cardiovasc Dis.*1980;23:1-12.

123. Hill MD, Buchan AM for the CASES Investigators. The Canadian Alteplase for Stroke Effectiveness Study (CASES). CMAJ 2005; 172: 1307-1312.

124. Pessin MS, Teal PA, Caplan LR.Hemorrhagic infarction: guilt by association? AJNR Am J Neuroradiol. 1991 Nov-Dec;12(6):1123-6. No abstract available. PMID: 1763738 [PubMed - indexed for MEDLINE]

125. Gaudino EA, Geisler MW, Squires NK. Construct validity in the Trailmaking Test: what makes Part B harder? J Clin Exp Neuropsychol.1995;17(4):529-35.

126. Radford LM, Chaney EF, O'Leary MR, O'Leary DE. Screening for cognitive impairment among inpatients. J Clin Psychiatry. 1978;39(9):712-5.

127. Rossini ED, Karl MA. The Trailmaking Test A and B: a technical note on structural nonequivalence. Percept Mot Skills. 1994;78(2):625-6.

128. Ginsberg MD, Hill MD, Palesch YY, Ryckborst KJ, Tamariz D: The ALIAS Pilot Trial: a dose-escalation and safety study of albumin therapy for acute ischemic stroke. I. Physiological responses and safety results. *Stroke*. 2006;37:2100-2106.

129. Palesch YY, Hill MD, Ryckborst KJ, Tamariz D, Ginsberg MD: The ALIAS Pilot Trial: a dose-escalation and safety study of albumin therapy for acute ischemic stroke. II. Neurological outcome and efficacy-analysis. *Stroke*. 2006;37:2107-2114.

130. Spiegelman, Hertzmark. Easy SAS calculations for risk or prevalence ratios and differences. Am J Epiemiol 2005; 162:199-200.
