# Supplementary material for: Albumin Administration in Acute Ischemic Stroke: Safety Analysis of the ALIAS Part 2 Multicenter Trial
Source: PLoS One. 2015 Sep 1;10(9):e0131390. doi: 10.1371/journal.pone.0131390 (PMC4556660; doi:10.1371/journal.pone.0131390)
Supplement: S1 Table — (DOCX) [file pone.0131390.s004.docx]

Supplemental Table I – Adverse Events among patients with sICH vs. non-ICH patients

|  | sICH patients | Patients without sICH | P* |
| --- | --- | --- | --- |
|  | N=41 | N=789 |  |
| Pulmonary edema/CHF ANY | 17.0% (7) | 9.1% (72) | 0.081 |
| Pulmonary edema/CHF within 48 hours | 12.2% (5) | 6.8% (54) | 0.204 |
| Acute coronary syndromes ANY | 2.4% (1) | 3.2% (25) | 1.000 |
| Acute coronary syndromes Within 48 hours | 2.4% (1) | 1.6% (13) | 0.485 |
| Troponin leak ANY^1^ | 19.5% (8) | 8.0% (63) | 0.018 |
| Troponin leak Within 48 hours | 17.1% (7) | 7.6% (60) | 0.040 |
| Atrial fibrillation ANY | 22.0% (9) | 9.6% (76) | 0.029 |
| Atrial fibrillation within 48 hours | 17.1% (7) | 5.6% (44) | 0.010 |
| Pneumonia ANY | 24.4% (10) | 8.5% (67) | 0.003 |
| Pneumonia within 7 days | 19.5% (8) | 5.7% (45) | 0.003 |
| Pulmonary embolus ANY | 7.3% (3) | 1.1% (9) | 0.019 |
| Pulmonary embolus within 7 days | 2.4% (1) | 0.1% (1) | 0.096 |

##### *Fisher exact test

sICH = symptomatic intracranial hemorrhage; CHF = congestive heart failure
