# Supplementary material for: Albumin Administration in Acute Ischemic Stroke: Safety Analysis of the ALIAS Part 2 Multicenter Trial
Source: PLoS One. 2015 Sep 1;10(9):e0131390. doi: 10.1371/journal.pone.0131390 (PMC4556660; doi:10.1371/journal.pone.0131390)
Supplement: S2 Table — (DOCX) [file pone.0131390.s005.docx]

Supplemental Table 2 – VISTA data population

|  | | CHF (n=52) | No CHF (n=4484) | P |
| --- | --- | --- | --- | --- |
| Demographics | |  |  |  |
|  | Age (n=4478) | 76.7 (8.5) | 70.2 (12.2) | 0.0001 |
|  | Female gender (n=4478) | 53.9% (28/52) | 47.4% (2125/4484) | 0.403 |
| Treatment | |  |  |  |
|  | Saline placebo (n=4484) | 90.4% (47/52) | 62.0% (2799/4484) | <0.0001 |
| Historical Risk Factors | |  |  |  |
|  | Hypertension (n=4356) | 87.5% (32/48) | 65.3% (2815/4308) | 0.001 |
|  | Atrial fibrillation (n=2774) | 81.8% (36/44) | 38.7% (1057/2730) | <0.0001 |
|  | Diabetes mellitus (n=4021) | 44.1% (15/34) | 22.7% (906/3987) | 0.006 |
|  | Current smoker (n=3636) | 20.0% (7/35) | 14.0% (503/3601) | 0.324 |
|  | High cholesterol (n=1690) | 0% (0/5) | 23.7% (399/1685) | 0.597 |
|  | Prior stroke (n=1946) | 20.0% (6/30) | 19.5% (374/1916) | 1.000 |
|  | Prior TIA** (n=1549) | 18.6% (8/43) | 14.5% (218/1506) | 0.508 |
|  | Alcohol (n=1690)* | 0% (0/5) | 9.4% (159/1685) | 1.0 |
|  | Past MI** (n=2683) | 33.3% (9/27) | 23.0% (612/2656) | 0.249 |
| Baseline Characteristics | |  |  |  |
|  | bNIHSS** (n=2353),median (iqr) | 20 (17-22) (n=31) | 14 (9-19) (n=2322) | <0.0001 |
|  | SBP** (n=4517), mean(sd) | 153 (29) (n=52) | 158 (27) (n=4465) | 0.157 |
|  | DBP** (n=4517), mean(sd) | 78 (14) (n=52) | 84 (17) (n=4465) | 0.011 |
|  | MAP** (n=4517), mean(sd) | 103 (16) (n=52) | 109 (18) (n=4465) | 0.022 |

* alcohol = > 2 drinks per day

**TIA = transient ischemic attack; MI = myocardial infarction; NIHSS = National Institutes of Health Stroke Scale; SBP – systolic blood pressure (in mmHg); DBP = diastolic blood pressure (in mmHg); MAP = mean arterial pressure (in mmHg)

In a secondary analysis examining only those 26 patients in whom the exact timing of CHF was known, the use of saline control (OR 6.5, CI_95_ 1.5-28.0), age and hypertension, but not baseline NIHSS scale score, were predictors of CHF within the first week. In a logistic regression model adjusted for age and sex, an episode CHF was associated with a reduced odds of independent outcome at 90 days (OR 0.40, CI_95_ 0.16-0.99, p=0.046).
